# Progression to type 1 diabetes in the DPT-1 and TN07 clinical trials is critically associated with specific residues in HLA-DQA1-B1 heterodimers

**DOI:** 10.1007/s00125-024-06274-6

**Published:** 2024-10-01

**Authors:** Lue Ping Zhao, George K. Papadopoulos, Jay S. Skyler, Alberto Pugliese, Hemang M. Parikh, William W. Kwok, Terry P. Lybrand, George P. Bondinas, Antonis K. Moustakas, Ruihan Wang, Chul-Woo Pyo, Wyatt C. Nelson, Daniel E. Geraghty, Åke Lernmark

**Affiliations:** 1 Public Health Sciences Division, Fred Hutchinson Cancer Research Center, Seattle, WA USA; 2 School of Public Health, University of Washington, Seattle, WA USA; 3 Laboratory of Biophysics, Biochemistry, Biomaterials and Bioprocessing, Faculty of Agricultural Technology, Technological Educational Institute (TEI) of Epirus, Arta, Greece; 4 Diabetes Research Institute and Division of Endocrinology, Diabetes & Metabolism, University of Miami Miler School of Medicine, Miami, FL USA; 5 Department of Diabetes Immunology, City of Hope, South Pasadena, CA USA; 6 Health Informatics Institute, Morsani College of Medicine, University of South Florida, Tampa, FL USA; 7 Benaroya Research Institute, Seattle, WA USA; 8 Department of Chemistry, Vanderbilt University, Nashville, TN USA; 9 Department of Food Science and Technology, Faculty of Environmental Sciences, Ionian University, Argostoli, Cephalonia Greece; 10 Clinical Research Division, Fred Hutchinson Cancer Research Center, Seattle, WA USA; 11 Department of Clinical Sciences, Lund University CRC, Skåne University Hospital, Malmö, Sweden

**Keywords:** Amino acids, HLA, Immunogenetics, Islet autoimmunity, Progression, Seroconversion, Type 1 diabetes

## Abstract

**Aims/hypothesis:**

The aim of this work was to explore molecular amino acids (AAs) and related structures of *HLA-DQA1-DQB1* that underlie its contribution to the progression from stages 1 or 2 to stage 3 type 1 diabetes.

**Methods:**

Using high-resolution *DQA1* and *DQB1* genotypes from 1216 participants in the Diabetes Prevention Trial-Type 1 and the Diabetes Prevention Trial, we applied hierarchically organised haplotype association analysis (HOH) to decipher which AAs contributed to the associations of DQ with disease and their structural properties. HOH relied on the Cox regression to quantify the association of DQ with time-to-onset of type 1 diabetes.

**Results:**

By numerating all possible DQ heterodimers of α- and β-chains, we showed that the heterodimerisation increases genetic diversity at the cellular level from 43 empirically observed haplotypes to 186 possible heterodimers. Heterodimerisation turned several neutral haplotypes (*DQ2.2*, *DQ2.3* and *DQ4.4*) to risk haplotypes (*DQ2.2/2.3-DQ4.4* and *DQ4.4-DQ2.2*). HOH uncovered eight AAs on the α-chain (−16α, −13α, −6α, α22, α23, α44, α72, α157) and six AAs on the β-chain (−18β, β9, β13, β26, β57, β135) that contributed to the association of DQ with progression of type 1 diabetes. The specific AAs concerned the signal peptide (minus sign, possible linkage to expression levels), pockets 1, 4 and 9 in the antigen-binding groove of the α1β1 domain, and the putative homodimerisation of the αβ heterodimers.

**Conclusions/interpretation:**

These results unveil the contribution made by DQ to type 1 diabetes progression at individual residues and related protein structures, shedding light on its immunological mechanisms and providing new leads for developing treatment strategies.

**Data availability:**

Clinical trial data and biospecimen samples are available through the National Institute of Diabetes and Digestive and Kidney Diseases Central Repository portal (https://repository.niddk.nih.gov/studies).

**Graphical Abstract:**

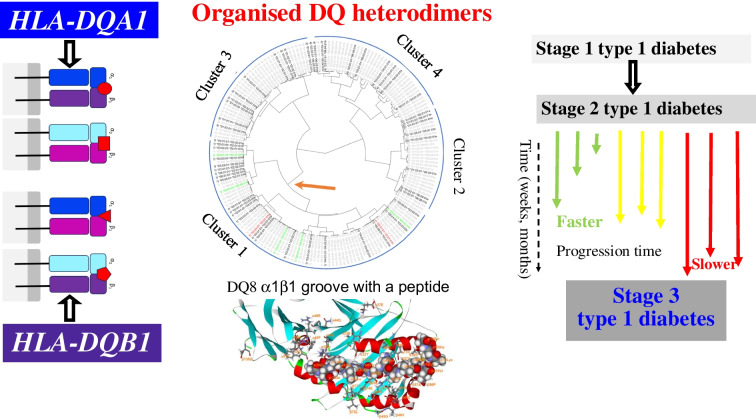

**Supplementary Information:**

The online version of this article (10.1007/s00125-024-06274-6) contains peer-reviewed but unedited supplementary material.


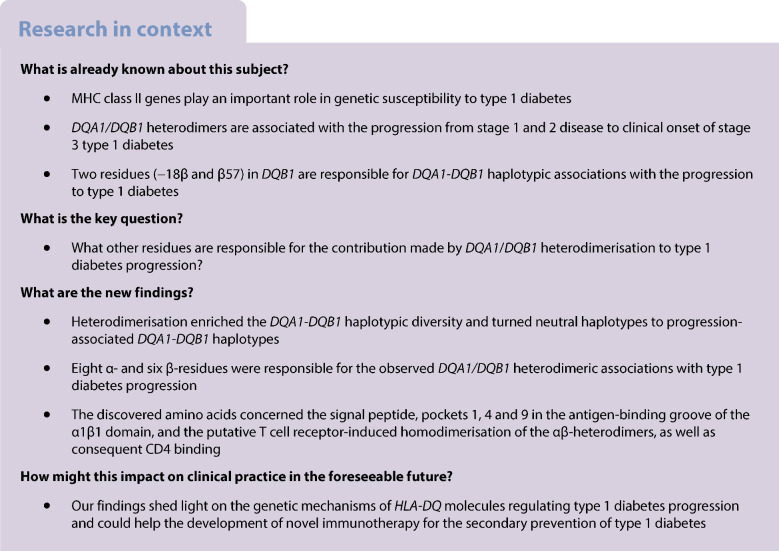


## Introduction

Type 1 diabetes is a life-long chronic autoimmune disease with an annual incidence among young people (under 20 years) of around 130,000 worldwide [1]. Its development typically runs from seroconversion (stage 1) to hyperglycaemia (stage 2), to onset of symptomatic type 1 diabetes (stage 3) [2]. The recent development of biochemically defined autoantibody markers allows us to screen for individuals at stage 1 or stage 2 disease (henceforward shortened to stage 1/2), facilitating secondary prevention [3] to interrupt the progression phase from stage 1/2 to stage 3 type 1 diabetes. This is the focus of the current investigation. Earlier, Butty and colleagues used archived clinical and genetic data from the Diabetes Prevention Trial-Type 1 (DPT-1) to study genetic determinants in the disease progression [4]. Their study uncovered the role of *DQB1* alleles in disease progression; carriers of the allele DQB1*03:02 were faster progressors, while DQB1*03:01 carriers were slower progressors [4]. To gain molecular insights into the role of specific amino acids (AAs) and related protein structures, we sequenced *DQA1* and *DQB1* genes in DNA samples from participants in the DPT-1 and uncovered two AAs (−18β, β57) that associated with the disease progression [5]. Recently, we acquired DNA samples from participants in the Oral Insulin Prevention Trial (TrialNet07 [TN07]) for sequencing and combined both DPT-1 and TN07 to investigate *DQ* associations; the results indicated a new association of *DQ2.5* with disease progression [6].

This new finding and the larger sample size provide an impetus to investigate further the molecular structure of *HLA-DQ*. The HLA system is critical to host immunity [7] through binding antigenic peptide fragments and presenting to T lymphocytes in the thymus (self-presentation) to avoid obvious autoimmune reactivity. Unfortunately, immune tolerance to certain autoantigenic peptides is incomplete and may, under certain circumstances and stimuli, lead to self-destruction of otherwise normal cells (i.e. autoimmune diseases). The vast majority of autoimmune diseases are associated with alleles from the HLA class II (HLAII) molecules that are each composed of an α-chain and a β-chain. The structure of these molecules is essentially preserved with minor but important differences: the so-called MHC fold (α1β1) consists of two anti-parallel α-helices on top of a β-sheet platform that shape an antigen-binding groove, open at both ends [8, 9]. The groove has five depressions, called pockets, positioned at relative positions 1, 4, 6, 7 and 9. The polymorphic residues of the various HLAII allelic molecules mainly concern several of the residues occupying these pockets. The second extracellular domain, α2β2, of HLAII molecules participates in accessory functions, such as binding to co-receptor molecule CD4 and presumed cognate T cell receptor (TCR)-mediated homodimerisation [10]. Identifying specific AAs within α- and β-chains, encoded by *DQA1/DQB1*, could shed new insights into genetic mechanisms that may be useful to understand the DQ binding process of autoantigen-derived peptides and hence to develop secondary prevention strategies. In this investigation, we integrated DPT-1 and TN07 cohorts into a single cohort known as iCohort, and studied the molecular structure of *DQA1-DQB1* in the context of the disease progression from stage 1/2 to stage 3 type 1 diabetes.

## Methods

### Diabetes prevention trials (DPT-1 and TN07)

DPT-1 and TN07 were randomised clinical trials to assess efficacy of oral/parenteral insulin for preventing type 1 diabetes or slowing disease progression among participants at moderate/high risk and have been detailed elsewhere [4, 6, 11]. Briefly, the integrated iCohort includes 408 type 1 diabetes onset events among 1216 participants with an accumulated 3916 person-years of observation. Demographically, most participants were white and there were 200 more male participants than female participants (see electronic supplementary material [ESM] Table 1). Most participants were under 20 years of age, while the rest were spread across the age range 20–46 years (ESM Fig. 1). Associations of risk factors with diabetes progression were assessed (ESM Table 1) and associated risk factors were treated as potential confounding variables.

Clinical trial data and biospecimen samples are available through the National Institute of Diabetes and Digestive and Kidney Diseases (NIDDK) Central Repository portal (https://repository.niddk.nih.gov/studies).

### Genotyping *HLA* using next-generation targeted sequencing technology

HLA typing was carried out using the Scisco HLA v6 typing kit (Scisco Genetics, Seattle, WA, USA) following the kit protocol. Briefly, the method employs an amplicon-based two-stage PCR, followed by sample pooling and sequencing using a MiSeq v2 PE500 (Illumina, San Diego, CA, USA). The protocol yielded three-field coverage of HLAII genes (*DRB1*, *DRB3*, *DRB4*, *DRB5*, *DQA1*, *DQB1*, *DPA1* and *DPB1*). The phase within each gene was determined in part by bridging amplicons and, when not available, by database lookup [12].

*DQA1* and *DQB1* are in strong linkage disequilibrium (LD), which is used to infer their haplotypes, referred to as *DQA1-DQB1*. Of the eight cases with ambiguously inferred haplotypes (six with posterior probability of 0.96 and two with less than 0.95 posterior probabilities), the haplotypes with the highest posterior probabilities were chosen for further analysis.

To maintain structural equivalence in DQ residues, we adopted the numbering system of Fremont et al for *H2-A* [13], as modified for DQ molecules [14]. In this manner, essentially all residues that occupied identical or near identical positions in the respective structures had the same residue numbers in the sequence, thus taking into account all possible insertions and deletions in certain alleles. AA abbreviations are presented mostly in the one-letter convention and occasionally in the three-letter convention.

Within the iCohort, the observed 43 *DQA1-DQB1* haplotypes were referred to a *cis*- or empirical haplotypes, in contrast to potentially new *trans*-haplotypes that are generated by ‘heterodimeric recombination’ and referred to as heterodimers. Computationally, we enumerated all possible *cis*- and *trans*-pairing of two *DQA1* alleles with two *DQB1* alleles, resulting in four heterodimers. For doubly heterozygous *DQA1* and *DQB1*, heterodimeric recombination could create two new *trans*-haplotypes, referred to as ‘heterodimeric recombinants’ (ESM Fig. 2). Some of heterodimeric recombinants were observed not to have any surface expressions, probably due to structural incompatibility of corresponding α-helices and β-sheet [15, 16], and many ‘forbidden’ haplotypes were found to have limited functional roles in hematopoietic cell transplantation [17].

We encourage all interested and qualified researchers to contact us for independent or collaborative research, with sequenced HLA genotype data, together with clinical data from NIDDK Central Repository.

### Molecular rendering and depiction

We used the coordinates of the *HLA-DQ8* hybrid peptide (HP) insulin C-peptide–islet amyloid polypeptide (IAPP) [18] in order to demonstrate the various AA residues shown by hierarchically organised haplotype association analysis (HOH) to be linked to progression towards type 1 diabetes or protection therefrom, among the DQ molecules found in the iCohort participants [6].

### Statistical analysis

All analyses relied on statistical functions and packages in R (https://www.r-project.org/). For haplotyping *DQA1* and *DQB1*, we used the ‘haplo.em’ function from the R package ‘haplo.stats’. For assessing genetic associations with the time-to-onset of type 1 diabetes, we used the ‘coxph’ function. There are three specific analytical approaches that are worth mentioning. First, analysis generally centres on associations of an individual allele (or haplotype) vs all others, to avoid the need for choosing a reference allele. Second, HOH consists of three analytical steps: (1) phylogenic analysis of AA sequences to hierarchically organise *DQ* alleles/haplotypes and to identify one or more relevant clusters (RCs) that are informative for identifying type 1 diabetes-associated AAs; (2) identifying polymorphic AAs within RCs and assessing AA-specific association analysis; and (3) selecting associated AAs to form motifs, evaluating the motif associations and interpreting associations in the context of DQ molecular structures. Finally, the current investigation, even though relying on RCTs, should be considered as an exploratory study, given multiple analyses concerning multiple alleles/haplotypes, multiple AAs in genes and multiple adjustments of covariates. In this analysis, we used the threshold of *p≤*0.05 to indicate a ‘nominal significance’ of corresponding statistic, to highlight risk, protective or neutral association without arbitrary correction of multiple comparisons.

## Results

### Replication of (−18β, β57) associations with type 1 diabetes progression

An earlier study from the DPT-1 uncovered two AAs (−18β and β57) in *DQB1* that associated with type 1 diabetes progression [5] (see DPT-1 [discovery] in ESM Table 2). Available TN07 data permitted an independent replication study of these two AAs. The AA β57A had a positive association with the onset of type 1 diabetes (HR 1.56, *p*=2.52×10^−4^) (ESM Table 2), a positive replication. Interestingly, β57D negatively associated with the type 1 diabetes onset (HR 0.50, *p*=9.83×10^−3^), in support of the marginal protective association observed in the discovery cohort (HR 0.70, *p*=0.074). When pooling the DPT-1 and TN07 data, β57A and β57D demonstrated associations with greater significance (HR 1.41 and 0.62, *p*=1.35×10^−5^ and 2.15×10^−3^, respectively) (ESM Table 2). On the other hand, the associations of −18βA and −18βV were not replicated in TN07 (HR 1.28 and 1.08, *p*=0.05 and 0.53, respectively). In fact, the association for −18βA was in the opposite direction from the discovery association (HR 0.82, *p*=0.06). Despite lacking this replication, the genetic association with −18βV in the pooled DPT-1 and TN07 sample remained significant (HR 1.30, *p*=0.013) (see Discussion for a possible explanation).

### Role of AA ‒18β in type 1 diabetes progression

The association with type 1 diabetes progression of AA ‒18βV in the discovery cohort (HR 1.47, *p*=2.81×10^−4^) was not replicated in TN07 (HR 1.08, *p*=0.53) (ESM Table 2), even though its association in the iCohort was positive (HR 1.30, *p*=0.0013) (Table 2, ESM Tables 2, 5). To decipher this peculiar phenomenon, we performed a stratified analysis in two groups of participants who had lower and higher risk (ESM Table 10) and found that the regulatory association of ‒18β was present only among high-risk individuals with both risk and resistance associations with ‒18βV and ‒18βA (HR 1.65 and 0.75, *p*=2.22×10^−4^ and 0.033, respectively). While this association needs to be independently validated, it is of importance to investigate the biological significance of ‒18β [19].

### Haplotypic associations of *HLA-DQ* with type 1 diabetes progression

Leveraging LD between *DQA1* and *DQB1*, we inferred their empirical haplotypes and assessed their association with the onset of type 1 diabetes and found that DQA1*03:01:01-DQB1*03:02:01 (*DQ8.1*) and DQA1*05:01:01-DQB1*02:01:01 (*DQ2.5*) had significant associations (HR 1.25 and 1.19, *p*=3.47×10^−3^ and 0.044, respectively), while DQA1*03:03:01-DQB1*03:01:01 had the opposite association (HR 0.55, *p*=1.19×10^−3^) (ESM Table 3).

Based on AA sequences specific to each *DQ* haplotype, HOH hierarchically organised 43 empirical haplotypes, including 17 common haplotypes (ten or more observed copies) and 26 rare haplotypes (with fewer than ten copies), and represented organised haplotypes in a fan-based tree. This tree is shown in ESM Fig. 3 in which *DQ* haplotypes were highlighted as resistance and risk haplotypes if *p*<0.05; haplotypes with insignificant associations (*p*>0.05) and all rare haplotypes are also shown. A single cluster, created by a phylogenic tree cutting threshold (indicated by the arrow in ESM Fig. 3), included two risk, one protective, six neutral and five rare haplotypes (ESM Table 4). Eliminating monomorphic AAs among nine common haplotypes (excluding five rare haplotypes) within this cluster led to seven polymorphic AAs in the α-chain (−6α, α22, α23, α31, α44 and α157) and 11 polymorphic AAs in the β-chain (−18β, −10β, β9, β13, β23, β26, β55, β57, β66, β71 and β135) (ESM Table 4; AAs in *DQ8.1* were considered as reference). Three AAs (α76, β9 and β23) were polymorphic, with only rarely occurring AAs in these positions among rare haplotypes. Additionally, β26 was in absolute LD with β13, and its association was represented by β13. Similarly β55, β66 and β71 were in absolute LD with −10β, and their associations were also represented by −10β. For every polymorphic AA, HOH directly assessed their associations with the onset of type 1 diabetes among those who carried those potentially risk haplotypes (ESM Table 5). AAs −18βV, β13G, β57A and β135D on the β-chain were found to positively associate with type 1 diabetes onset (HR 1.30, 1.39, 1.41 and 1.29, *p*=1.29×10^−3^, 3.09×10^−5^, 1.35×10^−5^ and 1.70×10^−3^, respectively), and so were AAs −6αM, α22Y, α31Q, α44C and α157A on the α-chain (HR 1.28, 1.31, 1.19, 1.19 and 1.30, *p*=3.83×10^−3^, 1.05×10^−3^, 0.044, 0.044 and 2.83×10^−4^, respectively). On the other hand, AAs β13A and β57D negatively associated with type 1 diabetes onset (HR 0.62 and 0.62, *p*=4.30×10^−3^ and 2.15×10^−3^, respectively).

### Heterodimeric associations of DQ with type 1 diabetes progression

As noted above, DQ heterodimers (ESM Fig. 2) consist of 186 unique haplotypes (ESM Table 6), which is more diverse than the empirically observed 43 *DQ* haplotypes (ESM Table 3). As shown in ESM Table 6, we created an ID number for each haplotype with a prefix of ‘e’ or ‘h’, corresponding to an empirical haplotype or a heterodimeric recombinant. Besides the formal nomenclature, we abbreviated these haplotypes in ESM Table 6 by eliminating ‘*DQA1*’ and ‘*DQB1*’ but adding ‘e’ or ‘h’ as a suffix to indicate empirical haplotype or heterodimeric recombinant, respectively. We also determined whether the corresponding heterodimeric recombinant was structurally forbidden (designated as ‘F’, or otherwise as ‘N’) [14–16]. In total, there were 74 ‘forbidden’ heterodimeric recombinants, 55 of which were considered rare, and 19 heterodimeric recombinants were observed 10–52 times in the iCohort. Note that a *DQ* haplotype from a heterodimerisation is the same as one of the empirical haplotypes and is still identified as an empirical haplotype. Without prejudicing against ‘forbidden’ DQ heterodimeric recombinants, we included these in further analyses.

Out of 186 haplotypes, there were 57 common haplotypes and 129 rare haplotypes that accounted for 321 observed heterodimers (ESM Table 7). Among common haplotypes, five of those listed in Table 1 (e1 [*03:01:01-*03:02:01], h3 [*05:01:01-*03:02:01], h4 [*03:01:01-*02:01:01], h46 [*02:01:01-*04:02:01] and h57 [*04:01:01-*02:02:01]) were positively associated with the type 1 diabetes (HR 1.11, 1.35, 1.32, 2.41 and 2.94, *p*=0.013, 0.0046, 0.012, 0.033 and 0.017, respectively). On the other hand, two *DQ* haplotypes (e6 [*03:03:01-*03:01:01] and h19 [*05:01:01-*03:01:01]) were negatively associated with the onset of type 1 diabetes (HR 0.66 and 0.42, *p*=0.0043 and 0.036, respectively).

**Table 1:** Assessment of the associations of heterodimeric *DQ* haplotypes with progression to type 1 diabetes

ID	Heterodimeric haplotype	Serotype	*n*	Coefficient	HR	SE	*z* score	*p* value
e1	*03:01:01-*03:02:01e	DQ8.1	1059	0.10	1.11	0.04	2.49	0.0129
e2	*05:01:01-*02:01:01e	DQ2.5	665	0.10	1.10	0.05	1.78	0.0754
h4	*03:01:01-*02:01:01h	DQ8.1-DQ2.5	267	0.28	1.32	0.11	2.50	0.0124
h3	*05:01:01-*03:02:01h	DQ2.5-DQ8.1	317	0.30	1.35	0.11	2.83	0.00462
e9	*02:01:01-*02:02:01e	DQ2.2	120	−0.13	0.88	0.17	−0.76	0.445
e45	*02:01:01-*03:03:02e	DQ2.3	12	−0.86	0.42	0.71	−1.21	0.227
e18	*04:01:01-*04:02:01e	DQ4.4	48	−0.07	0.93	0.27	−0.27	0.790
h46	*02:01:01-*04:02:01h	DQ2.2/2.3-DQ4.4	12	0.88	2.41	0.41	2.13	0.0331
h57	*04:01:01-*02:02:01h	DQ4.4-DQ2.2	10	1.08	2.94	0.45	2.38	0.0171
e6	*03:03:01-*03:01:01e	DQ7.3	178	−0.41	0.66	0.14	−2.85	0.00433
e8	*05:05:01-*03:01:01e	DQ7.5	123	−0.24	0.78	0.15	−1.63	0.102
h19	*05:01:01-*03:01:01h	DQ2.5-DQ7.3/7.5	47	−0.87	0.42	0.41	−2.09	0.0362

Frequency, regression coefficient, HR, SE, *z* score and *p* value corresponding to empirically observable haplotypes and heterodimeric recombinants, together with the corresponding serotype, are shown (data extracted from ESM Table 7; both tables share the same ID numbers)

Note that all association analyses are adjusted for age, risk level and study site

To gain insights into type 1 diabetes associations with both empirical haplotypes and heterodimeric recombinants, *DQ* haplotypes were extracted from ESM Table 7 and organised into three groups (Table 1). The first group comprised two empirical haplotypes (e1 [*03:01:01-*03:02:01e] and e2 [*05:01:01-*02:01:01e], commonly known as *DQ8.1* and *DQ2.5*, respectively) and two heterodimeric recombinants (*DQ8.1* and *DQ2.5*: h4 [*03:01:01-*02:01:01h] and h3 [*05:01:01-*03:02:01h], denoted as *DQ8.1-DQ.2.5* and *DQ.2.5-DQ8.1*, respectively). Both empirical haplotypes (*DQ8.1* and *DQ2.5*) retained positive associations with type 1 diabetes onset (HR 1.11 and 1.10, *p*=0.013 and 0.075, respectively), largely consistent with haplotypic associations (ESM Table 3). Not necessarily unexpectedly, the two heterodimeric recombinants, h4 (*03:01:01-*02:01:01h) and h3 (*05:01:01-*03:02:01h), exhibited stronger associations with type 1 diabetes onset (HR 1.32 and 1.35, *p*=0.012 and *p*=0.0046, respectively).

The second group shown in Table 1 (e9, e45, e18, h46 and h57) included three empirical haplotypes (*02:01:01-*02:02:01e, *02:01:01-*03:03:02e and *04:01:01-*04:02:01e) and two heterodimeric recombinants (*02:01:01-*04:02:01h and *04:01:01-*02:02:01h). A surprising pattern of haplotypic associations emerged, as while the three empirical haplotypes had no significant associations with the onset of type 1 diabetes (HR 0.88, 0.42 and 0.93, *p*=0.445, 0.23 and 0.79, respectively), the two heterodimeric recombinants had strong positive associations with the type 1 diabetes onset (HR 2.41 and 2.94, *p*=0.031 and 0.017, respectively).

In the third group shown in Table 1 (e6, e8 and h19), *DQ7.3/DQ7.5* haplotypes and their heterodimeric recombinants with *DQ2.5* appeared to retain their negative associations with type 1 diabetes onset (HR 0.66, 0.78 and 0.42, *p*=0.004, 0.10 and 0.036, respectively).

### *DQ* haplotype clusters and related AAs

**Fig. 1:**
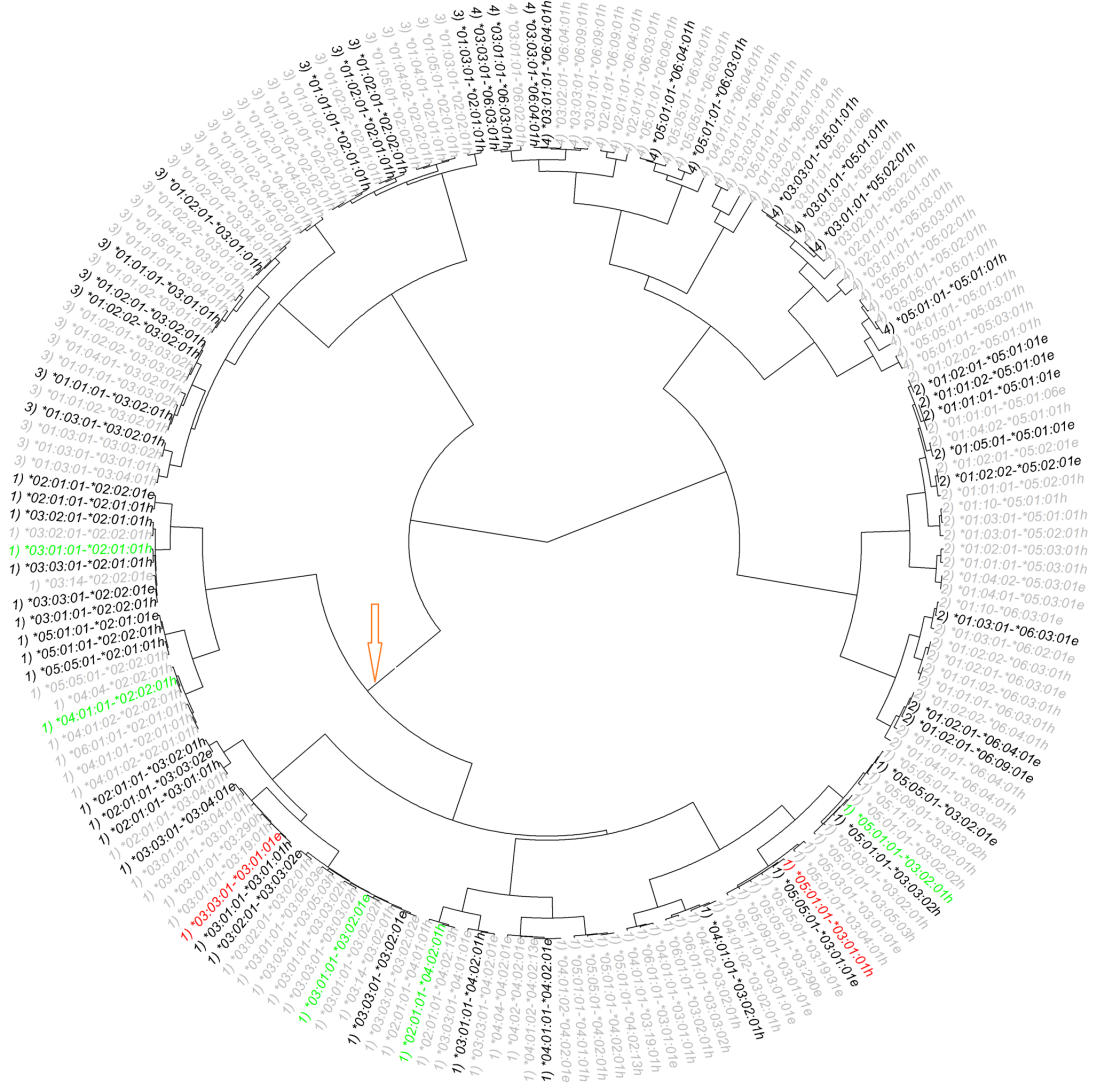
Fan-representation from phylogenic analysis of all heterodimeric *HLA-DQ* haplotypes based on similarity measurements between AA sequences of all individual haplotypes. A *DQ* haplotype is highlighted green or red if corresponding *p* values are <0.05 and HRs are ≥1, with red representing a risk haplotype and green representing a protective haplotype; a *DQ* haplotype is highlighted black if *p* values are ≥0.05 and the remaining *DQ* haplotypes are highlighted grey if they have fewer than ten copies. The brown arrow indicates the threshold for identifying a sub-phylogenic tree, resulting in clusters of heterodimeric *DQ* haplotypes. Note that all possible heterodimeric haplotypes are included, even though some may not be permissible

With diverse DQ heterodimers and emerging associations (Table 1 and ESM Table 7), it is expected that additional AAs contribute to their associations with type 1 diabetes. Here we applied HOH directly to hierarchically organise 186 DQ heterodimers (ESM Table 6) and display them in a fan-like hierarchical tree (Fig. 1) in which risk and protective haplotypes (*p* values <0.05), neutral haplotypes (*p* values ≥0.05) and rare haplotypes (fewer than ten copies) were highlighted. By choosing a threshold (brown arrow in Fig. 1), one has netted four clusters of *DQ* haplotypes labelled as clusters 1–4, and the cluster ID was placed as a prefix for each haplotype in the tree. From Fig. 1, it is clear that cluster 1 included all associated DQ heterodimers (both risk and protective haplotypes) and was naturally designated as an RC. This cluster included 29 common *DQ* haplotypes, in which 19 AAs (nine in the α-chain and ten in the β-chain) were polymorphic among these common haplotypes (ESM Table 8), excluding AAs pertaining to rare *DQ* haplotypes. Note that identified AAs of DQ heterodimers (ESM Table 8) are slightly different from those with *DQ* empirical haplotypes (ESM Table 4).

### AAs involved in heterodimeric associations with stage 3 type 1 diabetes onset

**Fig. 2:**
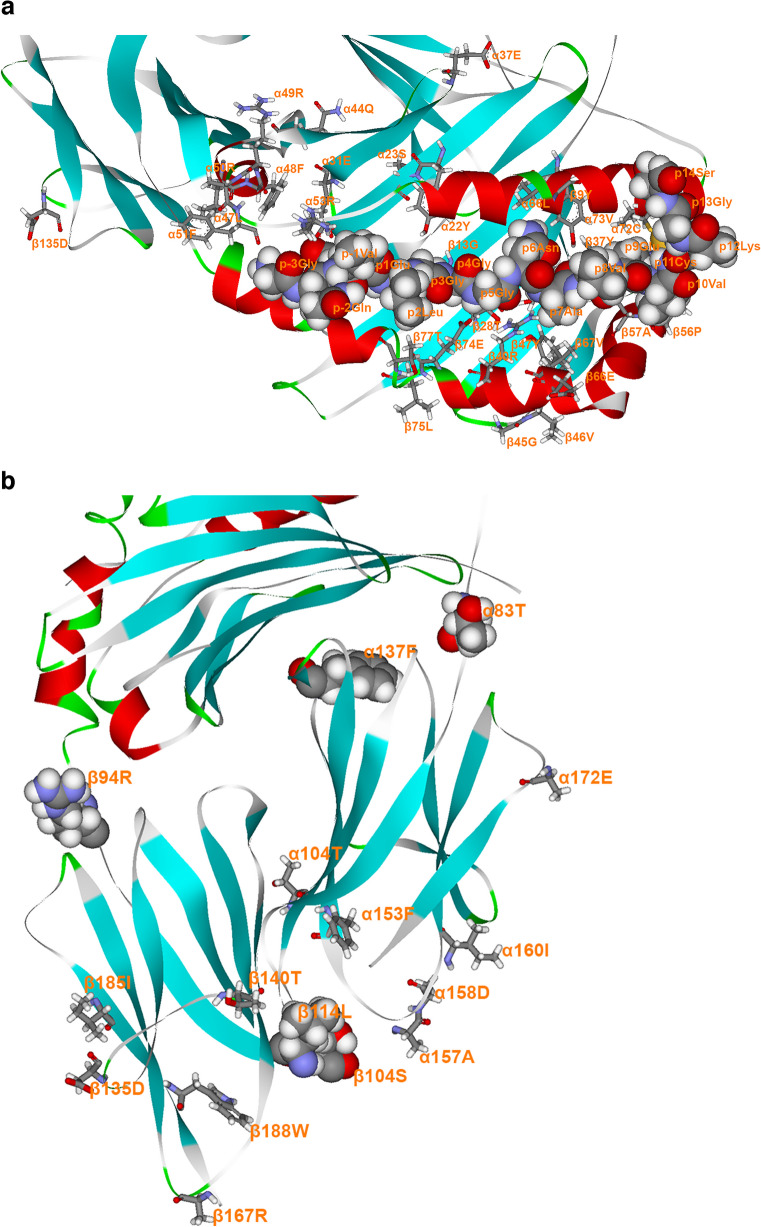
(**a**) Detailed view of the α1β1 domain of the HLA-DQ8-hybrid peptide insulin C-peptide−*IAPP* GQV**E**LG**G**G***NA****V****E****V*C*K* (anchors in bold, p11C mutated to form a disulphide bond with mutated DQα72C) complex (from https://www.rcsb.org/structure/6XCP, accessed 29 March 2024), showing all residues involved in type 1 diabetes progression or prevention thereof, among participants of the DPT-1 and TN07 trials, as revealed by HOH. Included are all residues in absolute LD to the involved residues among all the HLA-DQs. Residue β135D is added for orientation purposes. Depiction convention: protein backbone is shown in flat ribbons (α-helix, red; β-sheet, turquoise; β-turn, green; random coil, grey); antigenic peptide is shown in space-filling mode, and DQ residues are shown in stick mode (carbon, grey; oxygen, red; nitrogen, purple; hydrogen, white; sulphur, yellow). It is quite significant, in our view, that most of the depicted DQ8α1β1 residues line up pockets 4, 6, 7 and 9, or potentially can make contact with cognate TCR residues. Residues β26L, β30Y, β38A, β52P, β55P and β71T are covered mostly by the antigenic peptide or other DQ residues, in this orientation, hence cannot be seen. (**b**) Detailed view of α2β2 domain of HLA-DQ8–insulin C-peptide−*IAPP* hybrid peptide GQV**E**LG**G**G***NA****V****E****V*C*K* (anchors in bold) complex (from same source as above), in an orientation where the presumed interaction with CD4 would be such that the latter molecule has its long symmetry axis in a near vertical position. Residues α83T, α137F and β94R are in space-filling mode and shown only for orientation purposes; otherwise, the molecular depiction conventions are as in (**a**). The loop β105–113 is missing in the crystal structure, so the two immediately preceding residues, β104S and β114L, are shown in space-filling mode. Of the residues shown by HOH to be important for progression to type 1 diabetes or protection therefrom, plus those in absolute LD with the former, we note that α157 is involved in the putative cognate TCR-induced homodimerisation of MHC II molecules that promotes the binding of homodimer CD4 to such a complex, leading to T cell activation [10, 21]. In the respective β2 domain, residues β135 and β140 are part of the β134–148 stretch involved in CD4 binding, while residue β167R is part of the RGD loop found in all the known structures of HLA-DQ molecules possessing this tripeptide sequence. It is likely that residues involved in CD4 binding in the α2β2 domain, or others very close to them, interact with the inhibitory CD4 T cell membrane molecule LAG-3, which is inhibitory to the action of MHC II molecules [24]. Note that residues α172E and β167R have only partially determined crystallographic structure. Both figures drawn via the DSViewer Pro v.6 of Accelrys

To tease out which AAs were involved in the type 1 diabetes-associated DQ heterodimers, we performed AA associations among carriers of those haplotypes within the type 1 diabetes-associated cluster (Table 2). Other than AA α31 and ‒10β, β55, β66 and β71, the remaining eight AAs in the α-chain (‒16α, ‒13α, ‒6α, α22, α23, α44, α72 and α157) and six AAs in the β-chain (‒18β, β9, β13, β26, β57 and β135) were found to associate with type 1 diabetes onset (*p*<0.05) (Table 2). Note that two or more AAs of HLA-DQ are in absolute LD with each other within the RC, and only one is selected to represent them (ESM Table 9, ESM Fig. 4). To gain insights into these LD groups of AAs, we placed them on a protein structure of HLA-DQ8 in complex with an HP autoantigen (insulin C-peptide fragment plus IAPP fragment) (Fig. 2a, b). Quite a number of the residues in the α1β1 domain of the involved HLAII molecules are directly involved in antigenic peptide binding via any one of the five residue-anchoring pockets, or are TCR contact residues, or participate in the β49–56 homodimerisation stretch (Fig. 2a and ESM Table 9) [8, 10, 14, 20]. In the α2β2 domain of the HLA-DQ molecules under examination, we notice significant linkage to residues involved in binding to CD4 (β135 and β140), as well as to α157, a residue involved in the putative homodimerisation of HLA-DQ, and to β167 a part of the β167–69RGD loop whose function is still unknown [8, 10, 14, 20].

**Table 2:** Association results from assessing genetic associations of polymorphic AAs with the progression to type 1 diabetes

α-Chain									β-Chain								
Seq	Pos	AA	*n*	Coefficient	HR	SE	*z* score	*p* value	Seq	Pos	AA	*n*	Coefficient	HR	SE	*z* score	*p* value
1	−16α	L	160	−0.07	0.93	0.26	−0.27	0.787	1	‒18β	A	1998	0.00	1.00	0.07	−0.04	0.970
		M	4445	0.12	1.13	0.06	2.18	0.0289			V	2607	0.20	1.23	0.07	2.90	0.00378
2	‒13α	A	4268	0.14	1.15	0.06	2.43	0.0149	2	‒10β	A	3212	0.08	1.09	0.06	1.31	0.191
		T	337	−0.16	0.85	0.18	−0.89	0.372			S	1393	0.13	1.14	0.08	1.61	0.107
3	‒6α	M	4554	0.12	1.13	0.06	2.14	0.0326	3	β9	F	167	0.04	1.04	0.24	0.18	0.857
		T	51	−0.04	0.96	0.38	−0.11	0.910			Y	4438	0.12	1.13	0.06	2.09	0.0369
4	α22	F	246	−0.34	0.71	0.19	−1.75	0.0808	4	β13	A	605	−0.27	0.76	0.13	−2.16	0.0308
		Y	4359	0.16	1.17	0.06	2.73	0.00638			G	4000	0.19	1.20	0.06	3.23	0.00124
5	α23	S	2337	0.15	1.16	0.07	2.14	0.0326	5	β26	G	173	−0.01	0.99	0.24	−0.04	0.966
		T	2268	0.05	1.05	0.08	0.69	0.492			L	3827	0.19	1.21	0.06	3.28	0.00105
6	α31	E	2571	0.09	1.10	0.07	1.40	0.160			Y	605	−0.27	0.76	0.13	−2.16	0.0308
		Q	2034	0.12	1.13	0.08	1.49	0.135	6	β55	L	1393	0.13	1.14	0.08	1.61	0.107
7	α44	C	2034	0.12	1.13	0.08	1.49	0.135			P	3045	0.08	1.09	0.07	1.29	0.198
		K	234	−0.30	0.74	0.19	−1.56	0.119			R	167	0.04	1.04	0.24	0.18	0.857
		Q	2337	0.15	1.16	0.07	2.14	0.0326	7	β57	A	3773	0.21	1.23	0.06	3.61	0.000305
8	α72	I	2731	0.08	1.09	0.06	1.29	0.197			D	832	−0.29	0.75	0.11	−2.54	0.0111
		S	1874	0.13	1.14	0.08	1.60	0.109	8	β66	D	1560	0.13	1.14	0.08	1.62	0.106
9	α157	A	3899	0.14	1.15	0.06	2.51	0.0122			E	3045	0.08	1.09	0.07	1.29	0.198
		D	694	−0.08	0.92	0.12	−0.70	0.486	9	β71	D	167	0.04	1.04	0.24	0.18	0.8.57
		S	12	0.44	1.56	1.00	0.44	0.660			K	1393	0.13	1.14	0.08	1.61	0.107
											T	3045	0.08	1.09	0.07	1.29	0.1.98
									10	β135	D	4338	0.14	1.15	0.06	2.44	0.0148
											G	267	−0.16	0.85	0.18	−0.88	0.379

### Heterodimeric motif associations with type 1 diabetes onset

Based on eight α-chain and six β-chain AAs, HOH extracted motifs and assessed their associations with the onset of stage 3 type 1 diabetes (Table 3). There were 31 common motifs out of 53 motifs, and they were organised hierarchically and represented in a fan-like tree, in which tree leaves are denoted by one or more DQ heterodimer IDs sharing the same motif (ESM Fig. 5). By the hierarchical organisation, HOH suggested three clusters of heterodimers that included risk or protective haplotypes. Cluster c1 included two risk motifs (MAMYSQIAVYGLAD [e1, h101] and MAMYSQIAAYGLAD [h4], HR 1.11 and 1.32, *p*=0.014 and 0.012, respectively). Cluster c2 included a single risk motif (MAMYTCSAVYGLAD HR 1.35, *p*=0.0052), corresponding to two haplotypes [h3, h117]. Cluster c3 included one risk motif (MAMYTCSAAYAYAD [h69], HR 2.44, 0.049) and two protective motifs (MAMYSQIDAYAYDD [e6] and MAMYTCSAAYAYDD [h19], HR 0.66 and 0.42, *p*=0.004 and 0.036, respectively). It is of great interest in this respect, that protective h19 (DQA1*05:01/B1*03:01) and susceptible h69 (DQA1*05:01/B1*03:02) molecules differ by only one residue in their entire sequence (signal peptide plus mature chain) (i.e. β57Asp vs Ala, respectively. This is a case where the role of β57Asp becomes paramount in protection from progression to type 1 diabetes. Indeed, all motifs associated with resistance against progression to type 1 diabetes are β57Asp^+^ while all those associated with susceptibility to progression in this study are β57Asp^−^.

**Table 3:** Results from assessing associations of DQ motifs, within four sub-phylogenic trees, with the progression to type 1 diabetes

Seq	Motif	*n*	Coefficient	HR	SE	*z* score	*p* value	Haplotype set
1	MAMYSQIAVYGLAD	1204	0.10	1.11	0.04	2.46	0.0140	e1, h101
2	MAMYTCSAAYGLAD	710	0.10	1.10	0.05	1.78	0.0754	e2
3	MAMYTCSAVYGLAD	638	0.30	1.35	0.11	2.80	0.00517	h3, h117
4	MAMYSQIDVYGLAD	273	0.07	1.08	0.11	0.64	0.524	e5, e88, h169
5	MAMYSQIAAYGLAD	267	0.28	1.32	0.11	2.50	0.0124	h4
6	MAMYSQIDAYAYDD	195	−0.41	0.66	0.14	−2.85	0.00433	e6
7	MTMYTCSAAYAYDD	172	−0.21	0.81	0.14	−1.50	0.134	e8, e67, e178, e181, e183
8	MTMYTCSAVYGLAD	133	0.28	1.32	0.20	1.38	0.169	e14, h184
9	MAMFTKIAAYGLAG	123	−0.13	0.88	0.17	−0.76	0.445	e9
10	LAMYTCIAVFGGDD	100	−0.04	0.96	0.24	−0.17	0.865	e18, e111, e112, e114, e116
11	MAMYSQIAAYAYDD	93	0.03	1.03	0.20	0.16	0.875	h12, h85, h160
12	MAMYSQIDAYGLAD	93	0.09	1.10	0.19	0.48	0.632	h11
13	MAMYTCSAAYAYDD	77	−0.87	0.42	0.41	−2.09	0.0362	h19
14	MAMYTCSAAYGLAG	44	0.10	1.11	0.38	0.27	0.788	h35
15	MAMFTKIAVYGLAD	43	−0.31	0.73	0.27	−1.15	0.251	h21
16	MAMYSQIDAYGLAG	41	−0.31	0.73	0.33	−0.93	0.350	e22, e168
17	MAMYSQIAAYGLAG	38	−0.28	0.76	0.27	−1.01	0.311	h23
18	MAMYTCSAVYGLDD	34	−0.57	0.56	0.58	−0.99	0.323	h39
19	MATYSQIDVYGLDD	30	0.04	1.04	0.34	0.12	0.906	e34
20	LAMYTCIAVYGLAD	29	−0.06	0.94	0.36	−0.17	0.868	h33, h110, h113
21	MAMYSQIAVFGGDD	28	0.04	1.04	0.36	0.12	0.905	h29
22	MAMFTKIAAYGLAD	26	−0.41	0.66	0.41	−1.00	0.316	h30
23	MAMYSQIDAYAYAD	22	0.22	1.24	0.29	0.75	0.454	e38
24	MTMYTCSAAYGLAD	16	−0.65	0.52	0.58	−1.12	0.263	h40
25	MAMFTKIAAYAYDD	15	−0.88	0.42	0.71	−1.24	0.216	h41
26	MAMFTKIAVFGGDD	14	0.53	1.70	0.41	1.29	0.199	h46, h156, h157
27	LAMYTCIAAYGLAG	13	0.68	1.97	0.41	1.64	0.101	h57, h115, h173
28	MAMYSQIDVFGGDD	13	−0.15	0.86	0.54	−0.28	0.778	e62, e107
29	MAMFTKIAVYGLDD	12	−0.86	0.42	0.71	−1.21	0.227	e45
30	MAMYTCSAAYAYAD	12	0.89	2.44	0.45	1.97	0.0489	h69
31	MATYSQIDAYGLAD	10	0.21	1.23	0.58	0.36	0.722	h56

## Discussion

Using AA sequences of DQ heterodimers in the iCohort, we have carried out an exploratory analysis on genetic associations of residues on the α-chain and β-chain, and identified AAs that provide new insights into their genetic mechanisms. In addition, it is of interest to note that four AAs (−16α, −13α, −6α and −18β) concern residues in the respective signal peptides that could be involved in the efficiency of translation of the respective polypeptide chain. This is a subject that has not been adequately studied for HLAII [19]. The list of identified residues, plus residues in absolute LD to them in the alleles at hand, essentially contains residues involved in all known functions of HLAII molecules: antigen binding in the α1β1 domain groove; TCR contact; putative TCR-induced (αβ)_2_ homodimerisation; CD4 co-receptor binding (and perhaps lymphocyte-activation gene 3 binding); and cholesterol-raft clustering (see ESM Table 9) [8–10, 14, 20–25].

### HOH with and without inclusion of structurally forbidden heterodimers

As noted, the list of 186 DQ heterodimers in iCohort included 74 ‘forbidden’ heterodimeric recombinants, although 19 heterodimers were common, with the rest being rare heterodimers (ESM Table 6). Rare heterodimers make a limited contribution to type 1 diabetes. For those common forbidden DQ heterodimers, none of them were significantly associated with type 1 diabetes (*p*>0.05, ESM Table 7). Collectively, all ‘forbidden’ heterodimers made no contribution to type 1 diabetes progression, an observation consistent with that made in haematopoietic stem cell transplantation [17]. Further, we repeated HOH with D heterodimers, excluding 74 ‘forbidden’ heterodimers, and obtained a list of AAs the same as those presented in Table 2.

### Structural properties of identified AAs

It comes as no surprise, once again, that the HLA-DQ molecules constituting the main gene product leading to type 1 diabetes, are essential in people at risk: alleles predisposing towards type 1 diabetes to begin with, are predisposing in people at risk (with the exception of DQA1*05:01-DQB1*02:01), while alleles conferring protection do so in people at risk (see Conclusions below for explanation of lower HR values for progression/protection). It is remarkable that most AAs shown to be important by HOH for progression to type 1 diabetes or protection therefrom (ESM Table 9), as well as those in absolute LD in the alleles at hand, are involved in one or more of the known functions of HLAII molecules. In particular, the most highly statistically significant correlations involve four residues: at α22 (Tyr for susceptibility, Phe for resistance alleles, but without significance); at β13 (Gly for susceptibility, Ala for protective alleles); at β26 (Leu for susceptibility and Tyr for protective alleles); and β57 (Ala for susceptibility and Asp for protective alleles) (Table 2). These four residues participate in the formation of pockets 1 (α22, formation of a water-mediated hydrogen bond, in the case of Tyr, with the antigenic peptide backbone near pocket 1), 4 (β13 and β26) and 9 (β57), with sharply distinct anchor preferences. Specifically, β13Gly/β26Leu in the susceptible DQ8/*cis* and *trans* molecules (DQA1*03:01-DQB1*03:02 and DQA1*05:01-DQB1*03:02, respectively, and perhaps other DQA1*03/05 molecules with one or two substitutions) allow for large aliphatic and aromatic anchors in pocket 4. By contrast, the susceptible molecules DQ2/*cis* and *trans* (DQA1*05:01-DQB1*02:01 and DQA1*03:01-DQB1*02:01, respectively, and likewise other DQA1*03/05 alleles with one or two substitutions), and also β13Gly/β26Leu, prefer primarily acidic residues at p4 because of the β70Arg/71Lys/74Ala combination in this pocket, and secondarily large aliphatic and aromatic anchors [26–29]. On the other hand, β13Ala/β26Tyr in the resistant DQ7/*cis* and *trans* (DQA1*03:01-DQB1*03:01 and DQA1*05:01-DQB1*03:01, and likewise for other DQA1*03/05 molecules with one or two substitutions) allow only small anchors, such as Gly, Ala, Ser and Cys at pocket 4, while peptides with larger p4 anchors cannot bind [26]. In the case of pocket 9, the β57Asp/Ala dimorphism seen, respectively, in the vast majority of type 1 diabetes resistant vs susceptible *HLA-DQB1* molecules is responsible for the anchor choice of small aliphatic vs acidic residues. Furthermore, the DQ2 *cis*/*trans* molecules show an even higher preference of aromatic residues at p9 (even Trp) because their respective p9 pocket is the largest for all known MHC II molecules, due to the presence of β30Ser/β37Ile instead of β30Tyr/β37Tyr in DQ8 *cis*/*trans* molecules [27, 28, 30]. In addition to these residues, shown to be associated with significant predispositions for progression to disease or protection therefrom, other such residues in the α2β2 domain as well as the intramembranous domain are associated with different HLA-DQ functions (ESM Table 9), that might also make a difference in type 1 diabetes progression.

### Limitations

This is a post hoc analysis of an integrated iCohort from two randomised clinical trials, with newly generated HLA genotypes from all participants. While the randomisation obviates biases in allocations of participants, the post hoc nature implies that association results from *HLA-DQ* need to be independently replicated.

### Conclusions

The current investigation of participants in the DPT-1 and TN07 who probably have ongoing autoimmunity suggests that autoimmune processes involving HLA-DQ molecules are necessary for islet beta cell destruction, together with other subsequent processes (such as CD8 T cell attacks), leading at-risk individuals to progress to clinical type 1 diabetes. Insights on DQ molecular structures could stimulate further immunological investigation on beta cell destruction and hence new immunotherapies to halt or revert the type 1 diabetes progression [31].

## Supplementary Information

Below is the link to the electronic supplementary material.

ESM (PDF 968 KB)

## Data Availability

Clinical trial data and biospecimen samples are available through the NIDDK Central Repository portal (https://repository.niddk.nih.gov/studies); clinical data from participants in DPT-1 and TN07 are available from NIDDK Central Repository (https://repository.niddk.nih.gov/home/). Interested researchers are encouraged to contact corresponding authors for collaborative research on *HLA-DQ* genotype data.

## Abbreviations

AA: Amino acid
DPT-1: Diabetes Prevention Trial-Type 1
HLAII: HLA class II genes (*DR*, *DQ* and *DP*)
HOH: Hierarchically organised haplotype association analysis
HP: Hybrid peptide
IAPP: Islet amyloid polypeptide
LD: Linkage disequilibrium
NIDDK: National Institute of Diabetes and Digestive and Kidney Diseases
RC: Relevant cluster
TCR: T cell receptor
TN07: TrialNet07 ‘Randomised Diabetes Prevention Trial with Oral Insulin
