# Supplementary material for: Progression to type 1 diabetes in the DPT-1 and TN07 clinical trials is critically associated with specific residues in HLA-DQA1-B1 heterodimers
Source: Diabetologia. 2024 Oct 1;67(11):2481–93. doi: 10.1007/s00125-024-06274-6 (PMC11519105; doi:10.1007/s00125-024-06274-6)
Supplement: Supplementary file 1 — ESM (PDF 968 KB) [file 125_2024_6274_MOESM1_ESM.pdf]

Online Supplementary Materials

ESM Table 1. Demographic and clinical risk factors in the iCohort, computed frequencies, and association results include coefficient, hazard ratio, standard error, Z-score and p-value, from univariate analysis with one covariate a time (left panel) and multivariate analysis with all covariates (right panel). P-values, if less than 0.05, are highlighted red and green, if corresponding HR is less than 1 or greater than 1, respectively.

			Univariate Analysis					Multivariate Analysis				
**Variable**	**Value**	**n**	**coef**	**HR**	**SE**	**Z**	**p**	**coef**	**HR**	**SE**	**Z**	**p**
**Race**	White	1097	0.00	1.00				0.00	1.00			
	Others	119	-0.03	0.97	0.18	-0.17	8.65E-01	0.07	1.07	0.18	0.37	7.09E-01
**Sex**	Male	708	0.00	1.00				0.00	1.00			
	Female	508	0.12	1.13	0.10	1.21	2.26E-01	0.14	1.16	0.10	1.44	1.50E-01
**Age**	in 10 years	1216	-0.36	0.70	0.01	-4.70	2.65E-06	-0.53	0.59	0.08	-6.33	2.44E-10
**Risk Level**	Low	900	0.00	1.00				0.00	1.00			
	High	316	0.61	1.83	0.08	5.82	5.92E-09	0.87	2.39	0.13	6.49	8.54E-11
**insulin**	Placebo	606	0.00	1.00				0.00	1.00			
	Tretment	610	-0.18	0.83	0.10	1.83	6.76E-02	0.17	1.19	0.10	1.76	7.90E-02
**Study**	DPT1	670	0.00	1.00				0.00	1.00			
	TN07	546	-0.18	0.83	0.10	-1.77	7.71E-02	0.00	1.00	0.13	-0.02	9.84E-01

ESM Table 2. Allelic associations of HLA-A with the disease progression: allelic frequency, estimated coefficient, hazard ratio, standard error, Z-score and p-value without (left panel) and with (right panel) adjustment of HLAII and other confounding variables.

seq	HLA-A	n	Unadjusted					Adjusted				
			coef	HR	SE	z	p	coef	HR	SE	z	p
1	A*01:01	420	-0.02	0.98	0.09	-0.22	8.27E-01	-0.05	0.95	0.09	-0.53	5.98E-01
2	A*02:01	842	-0.15	0.86	0.08	-1.97	4.83E-02	-0.13	0.88	0.08	-1.78	7.59E-02
3	A*02:05	40	-0.29	0.75	0.34	-0.86	3.89E-01	-0.36	0.70	0.34	-1.08	2.80E-01
4	A*02:06	17	-0.95	0.39	0.71	-1.34	1.80E-01	-0.83	0.44	0.71	-1.17	2.42E-01
5	A*03:01	292	0.06	1.06	0.11	0.55	5.83E-01	0.09	1.09	0.11	0.81	4.21E-01
6	A*11:01	96	0.17	1.19	0.17	1.02	3.06E-01	0.07	1.07	0.17	0.40	6.86E-01
7	A*23:01	31	0.16	1.17	0.32	0.48	6.28E-01	0.28	1.33	0.32	0.88	3.79E-01
8	A*24:02	180	0.24	1.27	0.13	1.86	6.27E-02	0.35	1.42	0.12	2.83	4.71E-03
9	A*25:01	44	0.20	1.22	0.24	0.82	4.12E-01	0.12	1.13	0.24	0.50	6.20E-01
10	A*26:01	61	-0.17	0.84	0.23	-0.74	4.59E-01	-0.27	0.76	0.23	-1.18	2.38E-01
11	A*29:02	66	0.38	1.46	0.20	1.85	6.49E-02	0.28	1.32	0.21	1.34	1.80E-01
12	A*30:01	18	-0.12	0.89	0.45	-0.27	7.87E-01	0.03	1.03	0.45	0.06	9.55E-01
13	A*30:02	41	0.14	1.15	0.26	0.56	5.75E-01	-0.05	0.95	0.26	-0.18	8.56E-01
14	A*31:01	42	0.10	1.10	0.25	0.39	6.95E-01	-0.13	0.88	0.25	-0.53	5.98E-01
15	A*32:01	48	-0.43	0.65	0.32	-1.34	1.80E-01	-0.11	0.90	0.32	-0.33	7.43E-01
16	A*33:01	13	0.49	1.63	0.38	1.28	2.00E-01	0.41	1.51	0.38	1.07	2.82E-01
17	A*68:01	70	-0.04	0.96	0.20	-0.20	8.41E-01	0.10	1.11	0.20	0.51	6.12E-01
18	A*68:02	26	-0.12	0.89	0.36	-0.33	7.44E-01	0.05	1.05	0.36	0.14	8.85E-01
	rare (<10)	81	0.16	1.17	0.18	0.88	3.79E-01	0.07	1.07	0.18	0.39	6.99E-01

ESM Table 3. Allelic associations of HLA-B with the disease progression: allelic frequency, estimated coefficient, hazard ratio, standard error, Z-score and p-value without (left panel) and with (right panel) adjustment of HLAII and other confounding variables.

			Unadjusted					Adjusted				
seq	HLA-B	n	coef	HR	SE	z	p	coef	HR	SE	z	p
1	B*07:02	158	-0.08	0.93	0.15	-0.52	6.04E-01	-0.14	0.87	0.15	-0.94	3.45E-01
2	B*08:01	437	0.10	1.10	0.09	1.07	2.85E-01	0.06	1.06	0.09	0.62	5.33E-01
3	B*13:02	30	-0.24	0.78	0.36	-0.69	4.93E-01	0.44	1.55	0.36	1.22	2.23E-01
4	B*14:01	16	-0.09	0.92	0.45	-0.19	8.46E-01	-0.32	0.73	0.45	-0.71	4.81E-01
5	B*14:02	51	-0.01	0.99	0.26	-0.02	9.81E-01	-0.11	0.90	0.26	-0.42	6.77E-01
6	B*15:01	328	-0.02	0.98	0.10	-0.23	8.22E-01	-0.14	0.87	0.10	-1.37	1.71E-01
7	B*15:18	12	0.45	1.56	0.50	0.89	3.74E-01	0.77	2.17	0.50	1.54	1.24E-01
8	B*18:01	153	0.42	1.52	0.13	3.27	1.07E-03	0.19	1.21	0.13	1.50	1.35E-01
9	B*27:05	72	-0.15	0.86	0.20	-0.75	4.51E-01	-0.11	0.89	0.20	-0.56	5.76E-01
10	B*35:01	85	-0.05	0.95	0.18	-0.27	7.88E-01	0.07	1.07	0.18	0.39	6.94E-01
11	B*35:03	32	0.11	1.11	0.26	0.40	6.89E-01	0.04	1.04	0.27	0.16	8.76E-01
12	B*35:08	11	0.27	1.31	0.45	0.60	5.47E-01	0.51	1.67	0.45	1.14	2.56E-01
13	B*37:01	22	-1.01	0.36	0.58	-1.74	8.18E-02	-1.25	0.29	0.58	-2.15	3.12E-02
14	B*38:01	44	0.16	1.17	0.21	0.75	4.51E-01	-0.01	0.99	0.22	-0.05	9.57E-01
15	B*39:01	28	-0.05	0.95	0.34	-0.15	8.81E-01	0.51	1.66	0.34	1.50	1.35E-01
16	B*39:06	34	0.12	1.13	0.27	0.44	6.63E-01	0.22	1.25	0.27	0.81	4.17E-01
17	B*40:01	187	0.04	1.04	0.12	0.29	7.72E-01	0.09	1.10	0.12	0.76	4.50E-01
18	B*40:02	28	-0.78	0.46	0.50	-1.55	1.20E-01	-0.07	0.93	0.51	-0.15	8.83E-01
19	B*41:01	11	-0.10	0.90	0.58	-0.18	8.59E-01	0.04	1.04	0.58	0.07	9.42E-01
20	B*44:02	201	-0.09	0.91	0.13	-0.71	4.80E-01	0.09	1.10	0.13	0.69	4.93E-01
21	B*44:03	57	0.08	1.09	0.23	0.36	7.20E-01	0.13	1.14	0.23	0.56	5.72E-01
22	B*45:01	17	-0.14	0.87	0.50	-0.27	7.87E-01	-0.46	0.63	0.50	-0.91	3.64E-01
23	B*49:01	27	0.32	1.37	0.36	0.89	3.75E-01	0.21	1.23	0.36	0.58	5.65E-01
24	B*50:01	31	0.24	1.27	0.31	0.78	4.34E-01	0.03	1.03	0.31	0.10	9.24E-01
25	B*51:01	101	-0.36	0.70	0.21	-1.74	8.20E-02	-0.31	0.73	0.21	-1.50	1.33E-01
26	B*52:01	12	-0.50	0.61	0.71	-0.70	4.83E-01	0.12	1.13	0.71	0.17	8.66E-01
27	B*53:01	12	0.31	1.36	0.50	0.61	5.39E-01	0.26	1.29	0.50	0.51	6.10E-01

28	B*55:01	41	-0.02	0.98	0.28	-0.07	9.44E-01	0.03	1.03	0.28	0.11	9.09E-01
29	B*56:01	17	-0.15	0.86	0.50	-0.30	7.61E-01	0.55	1.74	0.50	1.09	2.74E-01
30	B*57:01	21	-0.11	0.89	0.41	-0.27	7.86E-01	0.05	1.05	0.41	0.13	8.98E-01
31	B*58:01	12	-0.02	0.98	0.44	-0.04	9.65E-01	-0.06	0.94	0.43	-0.14	8.92E-01
	rare (<10)	140	-0.16	0.85	0.16	-1.03	3.02E-01	-0.14	0.87	0.16	-0.86	3.88E-01

ESM Table 4. Allelic associations of HLA-C with the disease progression: allelic frequency, estimated coefficient, hazard ratio, standard error, Z-score and p-value without (left panel) and with (right panel) adjustment of HLAII and other confounding variables.

			Unadjusted					Adjusted				
**seq**	**HLA-C**	**n**	**coef**	**HR**	**SE**	**z**	**p**	**coef**	**HR**	**SE**	**z**	**p**
1	C*01:02	75	-0.24	0.78	0.21	-1.14	2.53E-01	0.00	1.00	0.22	0.00	9.97E-01
2	C*02:02	82	-0.45	0.64	0.24	-1.91	5.58E-02	-0.36	0.70	0.24	-1.49	1.37E-01
3	C*03:03	161	0.04	1.04	0.14	0.28	7.76E-01	-0.03	0.97	0.14	-0.22	8.24E-01
4	C*03:04	398	-0.01	0.99	0.09	-0.10	9.24E-01	-0.05	0.95	0.09	-0.55	5.81E-01
5	C*04:01	146	-0.17	0.84	0.14	-1.22	2.21E-01	-0.09	0.91	0.15	-0.61	5.40E-01
6	C*05:01	255	0.06	1.06	0.11	0.54	5.87E-01	-0.02	0.98	0.11	-0.22	8.27E-01
7	C*06:02	131	-0.08	0.93	0.17	-0.46	6.43E-01	-0.12	0.89	0.17	-0.71	4.80E-01
8	C*07:01	517	0.14	1.15	0.09	1.60	1.11E-01	0.12	1.13	0.09	1.38	1.67E-01
9	C*07:02	220	-0.07	0.93	0.12	-0.59	5.57E-01	0.01	1.01	0.13	0.08	9.39E-01
10	C*07:04	36	-0.09	0.92	0.31	-0.29	7.75E-01	0.28	1.32	0.31	0.91	3.64E-01
11	C*08:02	64	-0.01	0.99	0.23	-0.03	9.80E-01	-0.13	0.88	0.23	-0.58	5.62E-01
12	C*12:02	11	-0.39	0.68	0.71	-0.54	5.86E-01	0.16	1.17	0.71	0.22	8.24E-01
13	C*12:03	116	0.32	1.37	0.14	2.30	2.16E-02	0.22	1.24	0.14	1.52	1.29E-01
14	C*14:02	26	0.14	1.15	0.34	0.41	6.82E-01	0.17	1.18	0.34	0.49	6.25E-01
15	C*15:02	48	-0.46	0.63	0.28	-1.65	9.83E-02	-0.25	0.78	0.28	-0.89	3.72E-01
16	C*15:05	13	-0.27	0.77	0.50	-0.53	5.96E-01	-0.69	0.50	0.50	-1.37	1.70E-01
17	C*16:01	55	0.01	1.01	0.25	0.05	9.58E-01	0.04	1.04	0.25	0.17	8.61E-01
18	C*16:02	11	0.61	1.83	0.41	1.47	1.41E-01	0.59	1.81	0.41	1.44	1.50E-01
	rare (<10)	63	0.11	1.12	0.21	0.52	6.03E-01	0.11	1.12	0.21	0.54	5.87E-01

ESM Table 5. Ligand associations ofKIR genes with the disease progression: ligand numbers and their frequency, estimated coefficient, hazard ratio, standard error, Z-score and p-value without (left panel) and with (right panel) adjustment of HLAII and other confounding variables

seq	KIR	receptor number|frequency	Unadjusted					Adjusted				
			coef	HR	SE	z	p	coef	HR	SE	z	p
1	KIR2DL1	0|30, 1|365, 2|800, 3|13	-0.06	0.94	0.09	-0.69	4.93E-01	-0.03	0.97	0.09	-0.30	7.66E-01
2	KIR2DL2	0|607, 1|495, 2|106	0.03	1.03	0.08	0.39	7.00E-01	0.02	1.02	0.08	0.25	8.03E-01
3	KIR2DL3	0|108, 1|503, 2|597	-0.04	0.96	0.08	-0.53	5.98E-01	-0.03	0.97	0.08	-0.43	6.66E-01
4	KIR2DL4	1|38, 2|1132, 3|38	-0.24	0.79	0.20	-1.18	2.38E-01	0.03	1.04	0.19	0.18	8.59E-01
5	KIR2DL5	0|615, 1|406, 2|150, 3|35, 4|2	0.05	1.06	0.06	0.89	3.75E-01	0.06	1.06	0.06	0.90	3.68E-01
6	KIR2DS1	0|748, 1|404, 2|56	0.11	1.12	0.08	1.33	1.84E-01	0.11	1.11	0.08	1.25	2.13E-01
7	KIR2DS2			1.04	0.08	0.57	5.67E-01	0.04	1.04	0.08	0.56	5.73E-01
8	KIR2DS3	0|890, 1|244, 2|70, 3|2, 4|2	0.02	1.02	0.08	0.25	8.04E-01	0.02	1.02	0.08	0.21	8.36E-01
9	KIR2DS4	0|57, 1|408, 2|743	-0.10	0.90	0.08	-1.22	2.23E-01	-0.10	0.91	0.09	-1.14	2.53E-01
10	KIR2DS5	0|827, 1|343, 2|37, 3|1	0.11	1.11	0.09	1.14	2.54E-01	0.10	1.11	0.09	1.12	2.63E-01
11	KIR3DL1	0|56, 1|404, 2|748	-0.11	0.90	0.08	-1.26	2.06E-01	-0.10	0.90	0.09	-1.17	2.41E-01
12	KIR3DL2	1|14, 2|1193, 3|1	0.28	1.32	0.55	0.50	6.16E-01	0.30	1.35	0.54	0.56	5.77E-01
13	KIR3DS1	0|763, 1|377, 2|67, 3|1	0.06	1.07	0.08	0.77	4.42E-01	0.11	1.11	0.08	1.25	2.10E-01
14	KIR2DP1	0|28, 1|358, 2|822	-0.06	0.94	0.09	-0.67	5.05E-01	-0.04	0.96	0.09	-0.38	7.03E-01
15	KIR3DP1	1|39, 2|1132, 3|37	-0.21	0.81	0.20	-1.06	2.91E-01	0.06	1.06	0.19	0.32	7.48E-01

ESM Table 6. Interation of HLA-A with KIR ligand numbers with the disease progression without (left panel) and with (right panel) adjustment for confounding effects: allelic frequency (left panel) or interactive frequency (right panel), estimated coefficient, hazard ratio, standard error, Z-score and p-value.

seq	A-KIR-model	Eff. Size		Unadjusted					Adjusted				
		n0	n1	coef	HR	SE	z	p	coef	HR	SE	z	p
1	A*01:01-KIR2DL1-m2	251	121	-0.01	0.99	0.11	-0.11	9.15E-01	-0.06	0.94	0.11	-0.55	5.79E-01
2	A*02:01-KIR2DL1-m2	459	224	-0.17	0.84	0.10	-1.73	8.34E-02	-0.16	0.85	0.10	-1.64	1.01E-01
3	A*02:05-KIR2DL1-m2	29	8	-0.34	0.71	0.36	-0.94	3.46E-01	-0.43	0.65	0.36	-1.19	2.34E-01
4	A*02:06-KIR2DL1-m2	14	2	-0.93	0.40	0.71	-1.31	1.91E-01	-0.82	0.44	0.71	-1.16	2.48E-01
5	A*03:01-KIR2DL1-m2	171	95	0.07	1.07	0.12	0.60	5.50E-01	0.07	1.07	0.12	0.57	5.71E-01
6	A*11:01-KIR2DL1-m2	57	36	0.26	1.29	0.18	1.46	1.43E-01	0.13	1.14	0.18	0.74	4.56E-01
7	A*23:01-KIR2DL1-m2	20	10	0.18	1.19	0.32	0.55	5.80E-01	0.31	1.36	0.32	0.96	3.35E-01
8	A*24:02-KIR2DL1-m2	108	64	0.23	1.25	0.14	1.66	9.64E-02	0.38	1.47	0.14	2.80	5.18E-03
9	A*25:01-KIR2DL1-m2	26	17	0.17	1.19	0.25	0.70	4.84E-01	0.14	1.15	0.25	0.56	5.77E-01
10	A*26:01-KIR2DL1-m2	41	16	-0.26	0.77	0.26	-1.00	3.16E-01	-0.29	0.75	0.26	-1.13	2.58E-01
11	A*29:02-KIR2DL1-m2	37	20	0.34	1.41	0.23	1.48	1.39E-01	0.27	1.31	0.23	1.18	2.38E-01
12	A*30:01-KIR2DL1-m2	13	5	-0.12	0.89	0.45	-0.27	7.87E-01	0.03	1.03	0.45	0.07	9.46E-01
13	A*30:02-KIR2DL1-m2	25	15	0.11	1.11	0.26	0.40	6.87E-01	-0.07	0.93	0.26	-0.27	7.87E-01
14	A*31:01-KIR2DL1-m2	25	17	0.10	1.10	0.25	0.39	6.99E-01	-0.13	0.88	0.25	-0.53	5.99E-01
15	A*32:01-KIR2DL1-m2	37	10	-0.41	0.66	0.32	-1.28	1.99E-01	-0.10	0.91	0.32	-0.30	7.63E-01
16	A*33:01-KIR2DL1-m2	4	7	0.72	2.06	0.38	1.90	5.77E-02	0.58	1.79	0.38	1.53	1.26E-01
17	A*68:01-KIR2DL1-m2	42	25	0.01	1.01	0.21	0.06	9.54E-01	0.13	1.13	0.21	0.61	5.44E-01
18	A*68:02-KIR2DL1-m2	18	8	-0.12	0.89	0.36	-0.33	7.42E-01	0.05	1.05	0.36	0.14	8.88E-01
19	R10-KIR2DL1-m1	46	27	-0.75	0.47	1.07	-0.70	4.86E-01	-1.15	0.32	1.07	-1.07	2.83E-01
20	A*01:01-KIR2DL2-m2	126	62	0.00	1.00	0.14	0.03	9.80E-01	-0.06	0.94	0.14	-0.46	6.46E-01
21	A*02:01-KIR2DL2-m2	230	113	-0.10	0.91	0.11	-0.89	3.74E-01	-0.09	0.91	0.11	-0.85	3.93E-01
22	A*02:05-KIR2DL2-m2	17	4	-0.38	0.68	0.50	-0.76	4.47E-01	-0.52	0.60	0.50	-1.03	3.04E-01
23	A*02:06-KIR2DL2-m2	10	2	-0.40	0.67	0.71	-0.56	5.77E-01	-0.15	0.86	0.71	-0.21	8.36E-01
24	A*03:01-KIR2DL2-m1	95	48	-0.19	0.83	0.23	-0.81	4.19E-01	-0.15	0.86	0.23	-0.63	5.29E-01
25	A*11:01-KIR2DL2-m2	32	20	0.32	1.38	0.23	1.40	1.61E-01	0.14	1.15	0.23	0.59	5.56E-01

26	A*23:01-KIR2DL2-m2	13	6	0.12	1.13	0.41	0.29	7.70E-01	0.37	1.45	0.41	0.90	3.66E-01
27	A*24:02-KIR2DL2-m1	54	29	-0.28	0.75	0.27	-1.03	3.04E-01	-0.33	0.72	0.27	-1.22	2.22E-01
28	A*25:01-KIR2DL2-m1	14	9	-0.16	0.85	0.48	-0.34	7.35E-01	-0.13	0.88	0.48	-0.27	7.88E-01
29	A*26:01-KIR2DL2-m1	24	11	0.17	1.18	0.48	0.35	7.24E-01	0.28	1.32	0.48	0.58	5.61E-01
30	A*29:02-KIR2DL2-m2	20	12	0.46	1.59	0.29	1.58	1.13E-01	0.18	1.20	0.29	0.63	5.31E-01
31	A*30:01-KIR2DL2-m1	4	1	0.39	1.47	1.12	0.34	7.31E-01	1.48	4.38	1.13	1.31	1.90E-01
32	A*30:02-KIR2DL2-m1	9	10	0.42	1.53	0.53	0.81	4.20E-01	0.11	1.12	0.53	0.22	8.29E-01
33	A*31:01-KIR2DL2-m1	9	9	0.59	1.81	0.50	1.20	2.31E-01	0.66	1.93	0.50	1.32	1.86E-01
34	A*32:01-KIR2DL2-m1	18	6	0.48	1.61	0.65	0.73	4.66E-01	0.30	1.35	0.65	0.46	6.46E-01
35	A*33:01-KIR2DL2-m2	2	2	0.84	2.32	0.71	1.19	2.35E-01	0.79	2.19	0.71	1.11	2.68E-01
36	A*68:01-KIR2DL2-m1	23	10	-0.43	0.65	0.42	-1.03	3.04E-01	-0.20	0.82	0.42	-0.46	6.43E-01
37	A*68:02-KIR2DL2-m2	8	5	0.12	1.12	0.45	0.26	7.95E-01	0.08	1.09	0.45	0.18	8.56E-01
38	R10-KIR2DL2-m2	25	18	0.24	1.27	0.24	1.00	3.16E-01	0.07	1.08	0.24	0.30	7.61E-01
39	A*01:01-KIR2DL3-m2	238	113	-0.03	0.97	0.11	-0.27	7.84E-01	-0.08	0.93	0.11	-0.70	4.84E-01
40	A*02:01-KIR2DL3-m2	430	210	-0.15	0.86	0.10	-1.50	1.34E-01	-0.14	0.87	0.10	-1.40	1.60E-01
41	A*02:05-KIR2DL3-m2	26	7	-0.32	0.73	0.38	-0.84	4.04E-01	-0.52	0.59	0.38	-1.36	1.73E-01
42	A*02:06-KIR2DL3-m1	13	1	-1.72	0.18	1.42	-1.21	2.26E-01	-2.07	0.13	1.42	-1.45	1.46E-01
43	A*03:01-KIR2DL3-m1	163	87	-0.52	0.59	0.39	-1.35	1.76E-01	-0.24	0.79	0.39	-0.62	5.36E-01
44	A*11:01-KIR2DL3-m1	50	35	1.51	4.55	1.03	1.47	1.41E-01	1.36	3.90	1.03	1.32	1.86E-01
45	A*23:01-KIR2DL3-m1	18	8	-0.44	0.64	0.81	-0.55	5.84E-01	-0.65	0.52	0.81	-0.80	4.22E-01
46	A*24:02-KIR2DL3-m2	103	59	0.20	1.22	0.14	1.40	1.60E-01	0.35	1.42	0.14	2.47	1.36E-02
47	A*25:01-KIR2DL3-m1	24	15	-0.84	0.43	0.66	-1.28	2.00E-01	-0.39	0.67	0.66	-0.60	5.49E-01
48	A*26:01-KIR2DL3-m1	35	15	0.13	1.13	0.59	0.21	8.31E-01	0.50	1.65	0.59	0.84	3.98E-01
49	A*29:02-KIR2DL3-m2	36	19	0.37	1.45	0.24	1.59	1.13E-01	0.30	1.35	0.24	1.26	2.09E-01
50	A*30:01-KIR2DL3-m2	12	4	-0.31	0.74	0.50	-0.61	5.42E-01	-0.19	0.83	0.50	-0.38	7.07E-01
51	A*30:02-KIR2DL3-m1	24	14	-0.39	0.68	0.78	-0.50	6.15E-01	-1.02	0.36	0.78	-1.32	1.87E-01
52	A*31:01-KIR2DL3-m2	25	15	0.02	1.02	0.26	0.07	9.44E-01	-0.22	0.80	0.26	-0.82	4.11E-01
53	A*32:01-KIR2DL3-m1	34	9	0.01	1.01	1.07	0.01	9.93E-01	-0.57	0.57	1.07	-0.53	5.94E-01
54	A*33:01-KIR2DL3-m2	4	6	0.75	2.11	0.41	1.81	7.04E-02	0.55	1.73	0.41	1.33	1.83E-01
55	A*68:01-KIR2DL3-m2	38	23	0.05	1.05	0.21	0.23	8.19E-01	0.11	1.12	0.21	0.53	5.93E-01
56	A*68:02-KIR2DL3-m2	16	8	-0.03	0.97	0.36	-0.07	9.41E-01	0.14	1.15	0.36	0.39	6.99E-01
57	R10-KIR2DL3-m2	42	27	0.23	1.25	0.20	1.13	2.59E-01	0.09	1.09	0.20	0.45	6.54E-01

58	A*01:01-KIR2DL4-m2	258	125	-0.02	0.98	0.11	-0.21	8.37E-01	-0.05	0.95	0.11	-0.51	6.10E-01
59	A*02:01-KIR2DL4-m2	470	228	-0.18	0.83	0.10	-1.82	6.90E-02	-0.16	0.85	0.10	-1.63	1.03E-01
60	A*02:05-KIR2DL4-m2	31	8	-0.41	0.67	0.36	-1.14	2.54E-01	-0.48	0.62	0.36	-1.34	1.80E-01
61	A*02:06-KIR2DL4-m2	15	2	-0.95	0.39	0.71	-1.34	1.80E-01	-0.83	0.44	0.71	-1.17	2.41E-01
62	A*03:01-KIR2DL4-m2	174	97	0.08	1.09	0.12	0.70	4.84E-01	0.07	1.08	0.12	0.64	5.24E-01
63	A*11:01-KIR2DL4-m2	58	36	0.23	1.26	0.18	1.31	1.90E-01	0.11	1.12	0.18	0.63	5.32E-01
64	A*23:01-KIR2DL4-m2	21	10	0.16	1.17	0.32	0.49	6.27E-01	0.28	1.32	0.32	0.88	3.81E-01
65	A*24:02-KIR2DL4-m2	111	64	0.21	1.23	0.14	1.52	1.28E-01	0.38	1.46	0.14	2.77	5.56E-03
66	A*25:01-KIR2DL4-m2	26	18	0.20	1.22	0.24	0.82	4.14E-01	0.12	1.13	0.24	0.50	6.17E-01
67	A*26:01-KIR2DL4-m2	41	19	-0.15	0.86	0.24	-0.65	5.14E-01	-0.25	0.78	0.24	-1.05	2.93E-01
68	A*29:02-KIR2DL4-m2	41	22	0.33	1.40	0.22	1.52	1.30E-01	0.24	1.27	0.22	1.08	2.80E-01
69	A*30:01-KIR2DL4-m2	13	5	-0.12	0.89	0.45	-0.27	7.87E-01	0.03	1.03	0.45	0.07	9.46E-01
70	A*30:02-KIR2DL4-m2	25	16	0.14	1.15	0.26	0.56	5.79E-01	-0.05	0.96	0.26	-0.18	8.57E-01
71	A*31:01-KIR2DL4-m2	25	17	0.10	1.10	0.25	0.39	6.99E-01	-0.13	0.88	0.25	-0.53	5.99E-01
72	A*32:01-KIR2DL4-m2	38	10	-0.43	0.65	0.32	-1.34	1.79E-01	-0.11	0.90	0.32	-0.34	7.35E-01
73	A*33:01-KIR2DL4-m2	5	7	0.67	1.95	0.38	1.75	8.08E-02	0.56	1.75	0.38	1.46	1.45E-01
74	A*68:01-KIR2DL4-m2	43	25	0.00	1.00	0.21	0.02	9.85E-01	0.12	1.12	0.21	0.56	5.77E-01
75	A*68:02-KIR2DL4-m2	18	8	-0.12	0.89	0.36	-0.33	7.42E-01	0.05	1.05	0.36	0.14	8.88E-01
76	R10-KIR2DL4-m2	48	28	0.19	1.21	0.20	0.98	3.28E-01	0.10	1.11	0.20	0.53	5.93E-01
77	A*01:01-KIR2DL5-m1	128	63	0.05	1.06	0.22	0.25	8.02E-01	0.14	1.15	0.22	0.64	5.20E-01
78	A*02:01-KIR2DL5-m1	224	111	-0.10	0.91	0.20	-0.48	6.29E-01	-0.23	0.80	0.20	-1.13	2.57E-01
79	A*02:05-KIR2DL5-m1	14	6	0.99	2.70	0.82	1.21	2.27E-01	0.95	2.58	0.82	1.15	2.50E-01
80	A*02:06-KIR2DL5-m2	8	2	-0.38	0.69	0.71	-0.53	5.94E-01	-0.12	0.88	0.71	-0.17	8.61E-01
81	A*03:01-KIR2DL5-m2	79	49	0.18	1.20	0.15	1.21	2.26E-01	0.20	1.23	0.15	1.34	1.82E-01
82	A*11:01-KIR2DL5-m2	31	18	0.15	1.16	0.24	0.63	5.27E-01	0.09	1.09	0.24	0.37	7.09E-01
83	A*23:01-KIR2DL5-m1	13	5	-0.24	0.79	0.64	-0.38	7.07E-01	-0.55	0.58	0.64	-0.86	3.90E-01
84	A*24:02-KIR2DL5-m2	52	39	0.34	1.40	0.17	1.99	4.62E-02	0.50	1.65	0.17	2.97	3.00E-03
85	A*25:01-KIR2DL5-m2	13	8	0.25	1.28	0.36	0.70	4.84E-01	0.13	1.14	0.36	0.37	7.15E-01
86	A*26:01-KIR2DL5-m2	21	10	-0.16	0.85	0.32	-0.51	6.09E-01	-0.32	0.72	0.32	-1.01	3.14E-01
87	A*29:02-KIR2DL5-m2	17	8	0.16	1.18	0.36	0.46	6.46E-01	0.17	1.18	0.36	0.46	6.42E-01
88	A*30:01-KIR2DL5-m1	6	1	-1.00	0.37	1.12	-0.89	3.73E-01	-0.22	0.80	1.13	-0.20	8.42E-01
89	A*30:02-KIR2DL5-m1	11	9	-0.02	0.98	0.51	-0.03	9.76E-01	0.32	1.37	0.51	0.62	5.38E-01

90	A*31:01-KIR2DL5-m1	13	12	0.44	1.56	0.54	0.81	4.15E-01	0.19	1.21	0.54	0.36	7.22E-01
91	A*32:01-KIR2DL5-m1	16	7	0.79	2.20	0.70	1.13	2.58E-01	0.81	2.26	0.70	1.17	2.43E-01
92	A*33:01-KIR2DL5-m1	4	3	-0.61	0.54	0.77	-0.79	4.28E-01	-0.51	0.60	0.77	-0.67	5.06E-01
93	A*68:01-KIR2DL5-m1	24	11	-0.57	0.56	0.42	-1.37	1.70E-01	-0.12	0.89	0.42	-0.29	7.75E-01
94	A*68:02-KIR2DL5-m2	4	4	0.37	1.45	0.50	0.73	4.63E-01	0.41	1.51	0.50	0.81	4.16E-01
95	R10-KIR2DL5-m1	28	16	-0.40	0.67	0.40	-1.00	3.16E-01	-0.33	0.72	0.40	-0.84	4.03E-01
96	A*01:01-KIR2DS1-m1	103	52	0.01	1.01	0.22	0.06	9.51E-01	0.21	1.24	0.22	0.96	3.35E-01
97	A*02:01-KIR2DS1-m1	165	88	0.07	1.07	0.20	0.34	7.32E-01	-0.07	0.93	0.20	-0.34	7.36E-01
98	A*02:05-KIR2DS1-m1	12	5	0.42	1.52	0.74	0.56	5.72E-01	0.43	1.54	0.74	0.59	5.58E-01
99	A*02:06-KIR2DS1-m2	6	1	-0.75	0.47	1.00	-0.75	4.56E-01	-0.55	0.58	1.00	-0.55	5.84E-01
100	A*03:01-KIR2DS1-m2	60	37	0.17	1.18	0.17	0.97	3.31E-01	0.22	1.24	0.17	1.25	2.10E-01
101	A*11:01-KIR2DS1-m1	23	13	-0.37	0.69	0.36	-1.01	3.14E-01	-0.28	0.76	0.36	-0.77	4.41E-01
102	A*23:01-KIR2DS1-m2	8	3	0.34	1.41	0.58	0.59	5.54E-01	0.25	1.28	0.58	0.42	6.73E-01
103	A*24:02-KIR2DS1-m2	40	32	0.33	1.39	0.18	1.78	7.45E-02	0.46	1.58	0.18	2.49	1.29E-02
104	A*25:01-KIR2DS1-m2	7	8	0.64	1.90	0.36	1.80	7.26E-02	0.39	1.48	0.36	1.09	2.75E-01
105	A*26:01-KIR2DS1-m2	18	10	-0.01	0.99	0.32	-0.03	9.76E-01	-0.31	0.73	0.32	-0.98	3.28E-01
106	A*29:02-KIR2DS1-m2	12	5	0.06	1.06	0.45	0.13	8.97E-01	0.53	1.70	0.45	1.18	2.39E-01
107	A*30:01-KIR2DS1-m2	4	0										
108	A*30:02-KIR2DS1-m1	8	9	0.32	1.37	0.51	0.61	5.39E-01	0.51	1.67	0.52	0.99	3.21E-01
109	A*31:01-KIR2DS1-m2	11	11	0.26	1.30	0.31	0.86	3.88E-01	-0.09	0.91	0.31	-0.29	7.71E-01
110	A*32:01-KIR2DS1-m1	13	7	1.07	2.93	0.70	1.54	1.24E-01	1.05	2.86	0.70	1.51	1.32E-01
111	A*33:01-KIR2DS1-m2	4	4	0.63	1.89	0.50	1.26	2.07E-01	0.56	1.75	0.50	1.11	2.66E-01
112	A*68:01-KIR2DS1-m1	19	6	-0.95	0.39	0.48	-1.98	4.72E-02	-0.75	0.47	0.48	-1.56	1.18E-01
113	A*68:02-KIR2DS1-m2	2	3	0.52	1.68	0.58	0.89	3.71E-01	0.21	1.24	0.58	0.37	7.14E-01
114	R10-KIR2DS1-m2	20	13	0.27	1.31	0.28	0.96	3.39E-01	0.14	1.15	0.28	0.48	6.31E-01
115	A*01:01-KIR2DS2-m2	128	64	0.03	1.03	0.14	0.22	8.25E-01	-0.03	0.97	0.14	-0.24	8.14E-01
116	A*02:01-KIR2DS2-m2	231	115	-0.08	0.92	0.11	-0.77	4.41E-01	-0.08	0.93	0.11	-0.68	4.94E-01
117	A*02:05-KIR2DS2-m2	17	4	-0.38	0.68	0.50	-0.76	4.47E-01	-0.52	0.60	0.50	-1.03	3.04E-01
118	A*02:06-KIR2DS2-m2	9	2	-0.32	0.72	0.71	-0.46	6.48E-01	-0.09	0.92	0.71	-0.12	9.03E-01
119	A*03:01-KIR2DS2-m1	95	48	-0.22	0.80	0.23	-0.94	3.50E-01	-0.19	0.83	0.23	-0.80	4.26E-01
120	A*11:01-KIR2DS2-m2	33	21	0.34	1.40	0.22	1.50	1.34E-01	0.14	1.15	0.22	0.63	5.29E-01
121	A*23:01-KIR2DS2-m2	14	6	0.02	1.02	0.41	0.05	9.57E-01	0.26	1.30	0.41	0.64	5.25E-01

122	A*24:02-KIR2DS2-m1	54	29	-0.30	0.74	0.27	-1.10	2.69E-01	-0.34	0.71	0.27	-1.25	2.12E-01
123	A*25:01-KIR2DS2-m2	16	10	0.17	1.19	0.32	0.54	5.91E-01	0.11	1.11	0.32	0.33	7.39E-01
124	A*26:01-KIR2DS2-m2	25	11	-0.09	0.91	0.31	-0.30	7.68E-01	-0.14	0.87	0.31	-0.47	6.41E-01
125	A*29:02-KIR2DS2-m2	20	12	0.46	1.59	0.29	1.58	1.13E-01	0.18	1.20	0.29	0.63	5.31E-01
126	A*30:01-KIR2DS2-m2	4	1	0.22	1.25	1.00	0.22	8.25E-01	1.31	3.72	1.00	1.31	1.91E-01
127	A*30:02-KIR2DS2-m1	9	10	0.40	1.49	0.53	0.76	4.46E-01	0.08	1.09	0.53	0.16	8.74E-01
128	A*31:01-KIR2DS2-m1	9	9	0.45	1.56	0.50	0.90	3.67E-01	0.62	1.87	0.50	1.25	2.10E-01
129	A*32:01-KIR2DS2-m1	18	6	0.45	1.57	0.65	0.69	4.87E-01	0.27	1.31	0.65	0.41	6.80E-01
130	A*33:01-KIR2DS2-m2	2	2	0.84	2.32	0.71	1.19	2.35E-01	0.79	2.19	0.71	1.11	2.68E-01
131	A*68:01-KIR2DS2-m1	23	10	-0.46	0.63	0.42	-1.08	2.79E-01	-0.23	0.80	0.42	-0.54	5.90E-01
132	A*68:02-KIR2DS2-m2	8	5	0.12	1.12	0.45	0.26	7.95E-01	0.08	1.09	0.45	0.18	8.56E-01
133	R10-KIR2DS2-m2	25	19	0.27	1.31	0.24	1.16	2.47E-01	0.11	1.12	0.24	0.47	6.40E-01
134	A*01:01-KIR2DS3-m1	67	34	0.03	1.03	0.24	0.13	8.96E-01	-0.10	0.90	0.24	-0.42	6.74E-01
135	A*02:01-KIR2DS3-m1	123	59	-0.30	0.74	0.22	-1.36	1.75E-01	-0.39	0.68	0.22	-1.75	7.99E-02
136	A*02:05-KIR2DS3-m1	7	2	0.34	1.41	0.82	0.42	6.76E-01	0.96	2.61	0.83	1.16	2.45E-01
137	A*02:06-KIR2DS3-m2	3	2	0.36	1.43	0.71	0.50	6.15E-01	0.88	2.42	0.71	1.24	2.14E-01
138	A*03:01-KIR2DS3-m2	42	30	0.24	1.27	0.19	1.27	2.03E-01	0.33	1.39	0.19	1.72	8.46E-02
139	A*11:01-KIR2DS3-m2	18	10	0.24	1.27	0.32	0.76	4.49E-01	0.32	1.38	0.32	1.01	3.14E-01
140	A*23:01-KIR2DS3-m2	8	4	0.25	1.29	0.50	0.50	6.17E-01	0.24	1.27	0.50	0.48	6.34E-01
141	A*24:02-KIR2DS3-m2	26	22	0.25	1.28	0.22	1.13	2.57E-01	0.33	1.39	0.22	1.51	1.30E-01
142	A*25:01-KIR2DS3-m1	9	5	0.05	1.05	0.54	0.09	9.31E-01	-0.37	0.69	0.54	-0.68	4.98E-01
143	A*26:01-KIR2DS3-m2	15	4	-0.48	0.62	0.50	-0.95	3.44E-01	-0.71	0.49	0.50	-1.41	1.59E-01
144	A*29:02-KIR2DS3-m1	11	6	-0.13	0.88	0.49	-0.26	7.97E-01	-0.30	0.74	0.49	-0.62	5.38E-01
145	A*30:01-KIR2DS3-m2	3	1	0.81	2.25	1.00	0.81	4.19E-01	1.95	7.03	1.01	1.94	5.25E-02
146	A*30:02-KIR2DS3-m1	2	6	0.53	1.70	0.53	1.00	3.16E-01	0.46	1.59	0.53	0.87	3.82E-01
147	A*31:01-KIR2DS3-m1	3	6	0.57	1.76	0.52	1.09	2.75E-01	0.83	2.29	0.52	1.59	1.11E-01
148	A*32:01-KIR2DS3-m1	9	5	0.82	2.26	0.64	1.27	2.04E-01	0.68	1.97	0.64	1.06	2.91E-01
149	A*33:01-KIR2DS3-m2	1	2	1.11	3.04	0.71	1.57	1.17E-01	0.91	2.49	0.71	1.29	1.98E-01
150	A*68:01-KIR2DS3-m2	14	6	-0.39	0.67	0.41	-0.96	3.38E-01	0.15	1.16	0.41	0.36	7.18E-01
151	A*68:02-KIR2DS3-m2	4	1	-0.56	0.57	1.00	-0.56	5.77E-01	0.11	1.11	1.00	0.11	9.16E-01
152	R10-KIR2DS3-m1	12	4	-1.07	0.34	0.55	-1.93	5.38E-02	-0.75	0.47	0.55	-1.36	1.73E-01
153	A*01:01-KIR2DS4-m2	245	120	-0.01	0.99	0.11	-0.13	8.98E-01	-0.06	0.94	0.11	-0.58	5.65E-01

154	A*02:01-KIR2DS4-m2	447	218	-0.17	0.84	0.10	-1.74	8.19E-02	-0.16	0.85	0.10	-1.60	1.09E-01
155	A*02:05-KIR2DS4-m2	29	7	-0.43	0.65	0.38	-1.12	2.65E-01	-0.54	0.58	0.38	-1.41	1.58E-01
156	A*02:06-KIR2DS4-m2	14	2	-0.91	0.40	0.71	-1.28	2.00E-01	-0.83	0.44	0.71	-1.17	2.43E-01
157	A*03:01-KIR2DS4-m1	167	88	-1.07	0.34	0.47	-2.29	2.22E-02	-1.01	0.36	0.47	-2.16	3.06E-02
158	A*11:01-KIR2DS4-m2	53	34	0.23	1.26	0.18	1.30	1.94E-01	0.11	1.11	0.18	0.59	5.57E-01
159	A*23:01-KIR2DS4-m2	20	10	0.16	1.18	0.32	0.51	6.07E-01	0.30	1.35	0.32	0.94	3.48E-01
160	A*24:02-KIR2DS4-m2	107	62	0.21	1.23	0.14	1.50	1.32E-01	0.38	1.47	0.14	2.77	5.66E-03
161	A*25:01-KIR2DS4-m2	26	17	0.14	1.15	0.25	0.58	5.64E-01	0.11	1.12	0.25	0.46	6.46E-01
162	A*26:01-KIR2DS4-m2	40	19	-0.14	0.87	0.24	-0.61	5.43E-01	-0.25	0.78	0.24	-1.05	2.93E-01
163	A*29:02-KIR2DS4-m2	40	21	0.33	1.40	0.22	1.48	1.38E-01	0.21	1.24	0.23	0.94	3.49E-01
164	A*30:01-KIR2DS4-m2	13	5	-0.12	0.89	0.45	-0.27	7.87E-01	0.03	1.03	0.45	0.07	9.46E-01
165	A*30:02-KIR2DS4-m2	25	16	0.14	1.15	0.26	0.56	5.79E-01	-0.05	0.96	0.26	-0.18	8.57E-01
166	A*31:01-KIR2DS4-m2	25	16	0.05	1.05	0.26	0.19	8.52E-01	-0.17	0.84	0.26	-0.68	4.95E-01
167	A*32:01-KIR2DS4-m2	36	10	-0.39	0.68	0.32	-1.22	2.21E-01	-0.10	0.90	0.32	-0.31	7.54E-01
168	A*33:01-KIR2DS4-m2	5	7	0.67	1.95	0.38	1.75	8.08E-02	0.56	1.75	0.38	1.46	1.45E-01
169	A*68:01-KIR2DS4-m1	37	23	0.90	2.46	0.78	1.16	2.46E-01	1.13	3.11	0.78	1.46	1.44E-01
170	A*68:02-KIR2DS4-m2	17	8	-0.04	0.96	0.36	-0.10	9.20E-01	0.13	1.14	0.36	0.37	7.12E-01
171	R10-KIR2DS4-m2	46	27	0.18	1.20	0.20	0.92	3.60E-01	0.10	1.10	0.20	0.50	6.20E-01
172	A*01:01-KIR2DS5-m2	83	46	0.17	1.18	0.16	1.07	2.87E-01	0.26	1.30	0.16	1.66	9.74E-02
173	A*02:01-KIR2DS5-m2	139	70	0.05	1.05	0.13	0.38	7.03E-01	0.04	1.04	0.13	0.29	7.70E-01
174	A*02:05-KIR2DS5-m2	11	4	-0.26	0.77	0.50	-0.51	6.11E-01	-0.41	0.67	0.50	-0.81	4.21E-01
175	A*02:06-KIR2DS5-m2	7	1	-0.84	0.43	1.00	-0.84	4.02E-01	-0.62	0.54	1.00	-0.62	5.33E-01
176	A*03:01-KIR2DS5-m2	50	30	0.19	1.21	0.19	1.02	3.09E-01	0.21	1.23	0.19	1.09	2.74E-01
177	A*11:01-KIR2DS5-m1	17	11	-0.26	0.77	0.38	-0.69	4.92E-01	-0.30	0.74	0.38	-0.80	4.25E-01
178	A*23:01-KIR2DS5-m1	7	2	-0.50	0.60	0.80	-0.63	5.27E-01	-0.54	0.58	0.80	-0.67	5.01E-01
179	A*24:02-KIR2DS5-m2	33	22	0.36	1.43	0.22	1.64	1.01E-01	0.48	1.62	0.22	2.20	2.77E-02
180	A*25:01-KIR2DS5-m1	5	7	0.83	2.29	0.50	1.67	9.41E-02	0.99	2.70	0.50	2.00	4.54E-02
181	A*26:01-KIR2DS5-m2	13	9	0.12	1.13	0.34	0.37	7.12E-01	-0.10	0.91	0.34	-0.29	7.69E-01
182	A*29:02-KIR2DS5-m2	11	5	0.08	1.09	0.45	0.19	8.51E-01	0.50	1.65	0.45	1.11	2.66E-01
183	A*30:01-KIR2DS5-m2	4	0										
184	A*30:02-KIR2DS5-m1	9	4	-0.55	0.58	0.59	-0.94	3.48E-01	-0.09	0.91	0.59	-0.16	8.75E-01
185	A*31:01-KIR2DS5-m2	11	9	0.25	1.28	0.34	0.74	4.58E-01	-0.16	0.85	0.34	-0.47	6.35E-01

186	A*32:01-KIR2DS5-m1	9	5	0.85	2.35	0.64	1.33	1.84E-01	0.90	2.45	0.64	1.40	1.62E-01
187	A*33:01-KIR2DS5-m1	4	2	-1.22	0.30	0.84	-1.44	1.49E-01	-0.92	0.40	0.84	-1.09	2.76E-01
188	A*68:01-KIR2DS5-m1	16	5	-0.96	0.38	0.51	-1.87	6.09E-02	-0.73	0.48	0.51	-1.43	1.53E-01
189	A*68:02-KIR2DS5-m2	1	3	0.56	1.75	0.58	0.96	3.35E-01	0.24	1.28	0.58	0.42	6.73E-01
190	R10-KIR2DS5-m2	19	13	0.33	1.39	0.28	1.17	2.41E-01	0.20	1.22	0.28	0.71	4.79E-01
191	A*01:01-KIR3DL1-m2	245	120	-0.01	0.99	0.11	-0.13	8.98E-01	-0.06	0.94	0.11	-0.58	5.65E-01
192	A*02:01-KIR3DL1-m2	447	218	-0.17	0.84	0.10	-1.74	8.19E-02	-0.16	0.85	0.10	-1.60	1.09E-01
193	A*02:05-KIR3DL1-m2	29	7	-0.43	0.65	0.38	-1.12	2.65E-01	-0.54	0.58	0.38	-1.41	1.58E-01
194	A*02:06-KIR3DL1-m2	14	2	-0.91	0.40	0.71	-1.28	2.00E-01	-0.83	0.44	0.71	-1.17	2.43E-01
195	A*03:01-KIR3DL1-m1	167	88	-1.05	0.35	0.47	-2.26	2.40E-02	-1.00	0.37	0.47	-2.16	3.11E-02
196	A*11:01-KIR3DL1-m2	53	34	0.23	1.26	0.18	1.30	1.94E-01	0.11	1.11	0.18	0.59	5.57E-01
197	A*23:01-KIR3DL1-m2	20	10	0.16	1.18	0.32	0.51	6.07E-01	0.30	1.35	0.32	0.94	3.48E-01
198	A*24:02-KIR3DL1-m2	108	62	0.20	1.23	0.14	1.48	1.40E-01	0.38	1.47	0.14	2.76	5.77E-03
199	A*25:01-KIR3DL1-m2	26	17	0.14	1.15	0.25	0.58	5.64E-01	0.11	1.12	0.25	0.46	6.46E-01
200	A*26:01-KIR3DL1-m2	40	19	-0.14	0.87	0.24	-0.61	5.43E-01	-0.25	0.78	0.24	-1.05	2.93E-01
201	A*29:02-KIR3DL1-m2	40	21	0.33	1.40	0.22	1.48	1.38E-01	0.21	1.24	0.23	0.94	3.49E-01
202	A*30:01-KIR3DL1-m2	13	5	-0.12	0.89	0.45	-0.27	7.87E-01	0.03	1.03	0.45	0.07	9.46E-01
203	A*30:02-KIR3DL1-m2	25	16	0.14	1.15	0.26	0.56	5.79E-01	-0.05	0.96	0.26	-0.18	8.57E-01
204	A*31:01-KIR3DL1-m2	25	16	0.05	1.05	0.26	0.19	8.52E-01	-0.17	0.84	0.26	-0.68	4.95E-01
205	A*32:01-KIR3DL1-m2	36	10	-0.39	0.68	0.32	-1.22	2.21E-01	-0.10	0.90	0.32	-0.31	7.54E-01
206	A*33:01-KIR3DL1-m2	5	7	0.67	1.95	0.38	1.75	8.08E-02	0.56	1.75	0.38	1.46	1.45E-01
207	A*68:01-KIR3DL1-m1	38	23	0.84	2.31	0.78	1.08	2.81E-01	1.12	3.07	0.78	1.44	1.49E-01
208	A*68:02-KIR3DL1-m2	17	8	-0.04	0.96	0.36	-0.10	9.20E-01	0.13	1.14	0.36	0.37	7.12E-01
209	R10-KIR3DL1-m2	46	27	0.18	1.20	0.20	0.92	3.60E-01	0.10	1.10	0.20	0.50	6.20E-01
210	A*01:01-KIR3DL2-m2	258	125	-0.02	0.98	0.11	-0.21	8.37E-01	-0.05	0.95	0.11	-0.51	6.10E-01
211	A*02:01-KIR3DL2-m2	470	228	-0.18	0.83	0.10	-1.82	6.90E-02	-0.16	0.85	0.10	-1.63	1.03E-01
212	A*02:05-KIR3DL2-m2	31	8	-0.41	0.67	0.36	-1.14	2.54E-01	-0.48	0.62	0.36	-1.34	1.80E-01
213	A*02:06-KIR3DL2-m2	15	2	-0.95	0.39	0.71	-1.34	1.80E-01	-0.83	0.44	0.71	-1.17	2.41E-01
214	A*03:01-KIR3DL2-m2	174	97	0.08	1.09	0.12	0.70	4.84E-01	0.07	1.08	0.12	0.64	5.24E-01
215	A*11:01-KIR3DL2-m2	58	36	0.23	1.26	0.18	1.31	1.90E-01	0.11	1.12	0.18	0.63	5.32E-01
216	A*23:01-KIR3DL2-m2	21	10	0.16	1.17	0.32	0.49	6.27E-01	0.28	1.32	0.32	0.88	3.81E-01
217	A*24:02-KIR3DL2-m2	111	64	0.21	1.23	0.14	1.52	1.28E-01	0.38	1.46	0.14	2.77	5.56E-03

218	A*25:01-KIR3DL2-m2	26	18	0.20	1.22	0.24	0.82	4.14E-01	0.12	1.13	0.24	0.50	6.17E-01
219	A*26:01-KIR3DL2-m2	41	19	-0.15	0.86	0.24	-0.65	5.14E-01	-0.25	0.78	0.24	-1.05	2.93E-01
220	A*29:02-KIR3DL2-m2	41	22	0.33	1.40	0.22	1.52	1.30E-01	0.24	1.27	0.22	1.08	2.80E-01
221	A*30:01-KIR3DL2-m2	13	5	-0.12	0.89	0.45	-0.27	7.87E-01	0.03	1.03	0.45	0.07	9.46E-01
222	A*30:02-KIR3DL2-m2	25	16	0.14	1.15	0.26	0.56	5.79E-01	-0.05	0.96	0.26	-0.18	8.57E-01
223	A*31:01-KIR3DL2-m2	25	17	0.10	1.10	0.25	0.39	6.99E-01	-0.13	0.88	0.25	-0.53	5.99E-01
224	A*32:01-KIR3DL2-m2	38	10	-0.43	0.65	0.32	-1.34	1.79E-01	-0.11	0.90	0.32	-0.34	7.35E-01
225	A*33:01-KIR3DL2-m2	5	7	0.67	1.95	0.38	1.75	8.08E-02	0.56	1.75	0.38	1.46	1.45E-01
226	A*68:01-KIR3DL2-m2	43	25	0.00	1.00	0.21	0.02	9.85E-01	0.12	1.12	0.21	0.56	5.77E-01
227	A*68:02-KIR3DL2-m2	18	8	-0.12	0.89	0.36	-0.33	7.42E-01	0.05	1.05	0.36	0.14	8.88E-01
228	R10-KIR3DL2-m2	48	28	0.19	1.21	0.20	0.98	3.28E-01	0.10	1.11	0.20	0.53	5.93E-01
229	A*01:01-KIR3DS1-m2	102	49	0.02	1.02	0.15	0.15	8.84E-01	0.07	1.08	0.15	0.49	6.24E-01
230	A*02:01-KIR3DS1-m1	161	84	0.11	1.12	0.21	0.55	5.79E-01	-0.11	0.90	0.21	-0.51	6.10E-01
231	A*02:05-KIR3DS1-m1	12	3	-0.18	0.84	0.74	-0.24	8.09E-01	0.62	1.86	0.74	0.84	4.02E-01
232	A*02:06-KIR3DS1-m2	6	1	-0.78	0.46	1.00	-0.78	4.35E-01	-0.53	0.59	1.00	-0.53	5.98E-01
233	A*03:01-KIR3DS1-m2	60	38	0.16	1.17	0.17	0.93	3.52E-01	0.19	1.21	0.17	1.12	2.64E-01
234	A*11:01-KIR3DS1-m1	22	12	-0.35	0.71	0.37	-0.94	3.47E-01	-0.17	0.85	0.37	-0.45	6.55E-01
235	A*23:01-KIR3DS1-m2	8	3	0.34	1.41	0.58	0.59	5.54E-01	0.25	1.28	0.58	0.42	6.73E-01
236	A*24:02-KIR3DS1-m2	41	31	0.29	1.34	0.19	1.56	1.19E-01	0.46	1.59	0.19	2.48	1.32E-02
237	A*25:01-KIR3DS1-m2	6	8	0.81	2.26	0.36	2.28	2.26E-02	0.41	1.51	0.36	1.15	2.50E-01
238	A*26:01-KIR3DS1-m2	16	9	0.02	1.02	0.34	0.07	9.45E-01	-0.16	0.85	0.34	-0.47	6.36E-01
239	A*29:02-KIR3DS1-m1	13	4	-0.74	0.48	0.56	-1.32	1.87E-01	-0.21	0.81	0.57	-0.38	7.07E-01
240	A*30:01-KIR3DS1-m2	4	0										
241	A*30:02-KIR3DS1-m1	8	7	0.15	1.17	0.51	0.30	7.67E-01	0.39	1.48	0.52	0.76	4.45E-01
242	A*31:01-KIR3DS1-m2	12	10	0.18	1.20	0.32	0.58	5.65E-01	-0.11	0.89	0.32	-0.35	7.23E-01
243	A*32:01-KIR3DS1-m1	12	7	1.25	3.50	0.70	1.80	7.24E-02	1.11	3.03	0.70	1.59	1.12E-01
244	A*33:01-KIR3DS1-m1	4	3	-0.61	0.54	0.77	-0.79	4.27E-01	-0.54	0.58	0.77	-0.70	4.86E-01
245	A*68:01-KIR3DS1-m1	21	6	-0.98	0.38	0.48	-2.04	4.13E-02	-0.76	0.47	0.48	-1.58	1.14E-01
246	A*68:02-KIR3DS1-m1	2	2	0.48	1.62	0.82	0.59	5.57E-01	-0.14	0.87	0.82	-0.17	8.66E-01
247	R10-KIR3DS1-m2	18	10	0.13	1.14	0.32	0.40	6.89E-01	0.15	1.17	0.32	0.48	6.29E-01
248	A*01:01-KIR2DP1-m2	252	121	-0.02	0.98	0.11	-0.18	8.57E-01	-0.06	0.94	0.11	-0.56	5.76E-01
249	A*02:01-KIR2DP1-m2	461	224	-0.18	0.83	0.10	-1.82	6.92E-02	-0.17	0.85	0.10	-1.65	9.89E-02

250	A*02:05-KIR2DP1-m2	29	8	-0.34	0.71	0.36	-0.94	3.46E-01	-0.43	0.65	0.36	-1.19	2.34E-01
251	A*02:06-KIR2DP1-m2	14	2	-0.93	0.40	0.71	-1.31	1.91E-01	-0.82	0.44	0.71	-1.16	2.48E-01
252	A*03:01-KIR2DP1-m2	171	95	0.07	1.07	0.12	0.60	5.50E-01	0.07	1.07	0.12	0.57	5.71E-01
253	A*11:01-KIR2DP1-m2	57	36	0.26	1.29	0.18	1.46	1.43E-01	0.13	1.14	0.18	0.74	4.56E-01
254	A*23:01-KIR2DP1-m2	20	10	0.18	1.19	0.32	0.55	5.80E-01	0.31	1.36	0.32	0.96	3.35E-01
255	A*24:02-KIR2DP1-m2	108	64	0.23	1.25	0.14	1.66	9.64E-02	0.38	1.47	0.14	2.80	5.18E-03
256	A*25:01-KIR2DP1-m2	26	17	0.17	1.19	0.25	0.70	4.84E-01	0.14	1.15	0.25	0.56	5.77E-01
257	A*26:01-KIR2DP1-m2	41	16	-0.26	0.77	0.26	-1.00	3.16E-01	-0.29	0.75	0.26	-1.13	2.58E-01
258	A*29:02-KIR2DP1-m2	37	20	0.34	1.41	0.23	1.48	1.39E-01	0.27	1.31	0.23	1.18	2.38E-01
259	A*30:01-KIR2DP1-m2	13	5	-0.12	0.89	0.45	-0.27	7.87E-01	0.03	1.03	0.45	0.07	9.46E-01
260	A*30:02-KIR2DP1-m2	25	15	0.11	1.11	0.26	0.40	6.87E-01	-0.07	0.93	0.26	-0.27	7.87E-01
261	A*31:01-KIR2DP1-m2	25	17	0.10	1.10	0.25	0.39	6.99E-01	-0.13	0.88	0.25	-0.53	5.99E-01
262	A*32:01-KIR2DP1-m2	37	10	-0.41	0.66	0.32	-1.28	1.99E-01	-0.10	0.91	0.32	-0.30	7.63E-01
263	A*33:01-KIR2DP1-m2	4	7	0.72	2.06	0.38	1.90	5.77E-02	0.58	1.79	0.38	1.53	1.26E-01
264	A*68:01-KIR2DP1-m2	42	25	0.01	1.01	0.21	0.06	9.54E-01	0.13	1.13	0.21	0.61	5.44E-01
265	A*68:02-KIR2DP1-m2	18	8	-0.12	0.89	0.36	-0.33	7.42E-01	0.05	1.05	0.36	0.14	8.88E-01
266	R10-KIR2DP1-m1	46	27	-0.65	0.52	1.07	-0.61	5.43E-01	-1.14	0.32	1.07	-1.06	2.87E-01
267	A*01:01-KIR3DP1-m2	258	125	-0.02	0.98	0.11	-0.21	8.37E-01	-0.05	0.95	0.11	-0.51	6.10E-01
268	A*02:01-KIR3DP1-m2	470	228	-0.18	0.83	0.10	-1.82	6.90E-02	-0.16	0.85	0.10	-1.63	1.03E-01
269	A*02:05-KIR3DP1-m2	31	8	-0.41	0.67	0.36	-1.14	2.54E-01	-0.48	0.62	0.36	-1.34	1.80E-01
270	A*02:06-KIR3DP1-m2	15	2	-0.95	0.39	0.71	-1.34	1.80E-01	-0.83	0.44	0.71	-1.17	2.41E-01
271	A*03:01-KIR3DP1-m2	174	97	0.08	1.09	0.12	0.70	4.84E-01	0.07	1.08	0.12	0.64	5.24E-01
272	A*11:01-KIR3DP1-m2	58	36	0.23	1.26	0.18	1.31	1.90E-01	0.11	1.12	0.18	0.63	5.32E-01
273	A*23:01-KIR3DP1-m2	21	10	0.16	1.17	0.32	0.49	6.27E-01	0.28	1.32	0.32	0.88	3.81E-01
274	A*24:02-KIR3DP1-m2	111	64	0.21	1.23	0.14	1.52	1.28E-01	0.38	1.46	0.14	2.77	5.56E-03
275	A*25:01-KIR3DP1-m2	26	18	0.20	1.22	0.24	0.82	4.14E-01	0.12	1.13	0.24	0.50	6.17E-01
276	A*26:01-KIR3DP1-m2	41	19	-0.15	0.86	0.24	-0.65	5.14E-01	-0.25	0.78	0.24	-1.05	2.93E-01
277	A*29:02-KIR3DP1-m2	41	22	0.33	1.40	0.22	1.52	1.30E-01	0.24	1.27	0.22	1.08	2.80E-01
278	A*30:01-KIR3DP1-m2	13	5	-0.12	0.89	0.45	-0.27	7.87E-01	0.03	1.03	0.45	0.07	9.46E-01
279	A*30:02-KIR3DP1-m2	25	16	0.14	1.15	0.26	0.56	5.79E-01	-0.05	0.96	0.26	-0.18	8.57E-01
280	A*31:01-KIR3DP1-m2	25	17	0.10	1.10	0.25	0.39	6.99E-01	-0.13	0.88	0.25	-0.53	5.99E-01
281	A*32:01-KIR3DP1-m2	38	10	-0.43	0.65	0.32	-1.34	1.79E-01	-0.11	0.90	0.32	-0.34	7.35E-01

282	A*33:01-KIR3DP1-m2	5	7	0.67	1.95	0.38	1.75	8.08E-02	0.56	1.75	0.38	1.46	1.45E-01
283	A*68:01-KIR3DP1-m2	43	25	0.00	1.00	0.21	0.02	9.85E-01	0.12	1.12	0.21	0.56	5.77E-01
284	A*68:02-KIR3DP1-m2	18	8	-0.12	0.89	0.36	-0.33	7.42E-01	0.05	1.05	0.36	0.14	8.88E-01
285	R10-KIR3DP1-m2	48	28	0.19	1.21	0.20	0.98	3.28E-01	0.10	1.11	0.20	0.53	5.93E-01

ESM Table 7. Interation of HLA-B with KIR ligand numbers with the disease progression without (left panel) and with (right panel) adjustment for confounding effects: allelic frequency (left panel) or interactive frequency (right panel), estimated coefficient, hazard ratio, standard error, Z-score and p-value.

seq	B-KIR-model	Eff. Size		Unadjusted					Adjusted				
		n0	n1	coef	HR	SE	z	p	coef	HR	SE	z	p
1	B*07:02-KIR2DL1-m2	102	46	-0.13	0.87	0.16	-0.86	3.91E-01	-0.21	0.81	0.16	-1.35	1.77E-01
2	B*08:01-KIR2DL1-m1	257	135	-0.27	0.76	0.65	-0.41	6.78E-01	-0.61	0.54	0.65	-0.94	3.47E-01
3	B*13:02-KIR2DL1-m2	21	8	-0.23	0.79	0.36	-0.65	5.18E-01	0.46	1.58	0.36	1.27	2.03E-01
4	B*14:01-KIR2DL1-m2	11	5	-0.09	0.92	0.45	-0.20	8.45E-01	-0.32	0.73	0.45	-0.70	4.82E-01
5	B*14:02-KIR2DL1-m2	30	15	0.10	1.10	0.26	0.38	7.05E-01	0.00	1.00	0.26	0.01	9.94E-01
6	B*15:01-KIR2DL1-m2	199	100	-0.02	0.98	0.12	-0.18	8.56E-01	-0.15	0.86	0.12	-1.29	1.98E-01
7	B*15:18-KIR2DL1-m2	7	4	0.52	1.69	0.50	1.04	2.99E-01	0.89	2.44	0.50	1.77	7.64E-02
8	B*18:01-KIR2DL1-m2	82	62	0.39	1.48	0.14	2.84	4.54E-03	0.15	1.16	0.14	1.09	2.76E-01
9	B*27:05-KIR2DL1-m2	47	21	-0.18	0.83	0.22	-0.81	4.16E-01	-0.13	0.88	0.22	-0.58	5.63E-01
10	B*35:01-KIR2DL1-m2	47	30	-0.05	0.96	0.19	-0.24	8.10E-01	0.06	1.06	0.19	0.32	7.52E-01
11	B*35:03-KIR2DL1-m2	17	14	0.14	1.15	0.27	0.52	6.06E-01	0.07	1.07	0.27	0.25	8.06E-01
12	B*35:08-KIR2DL1-m2	6	5	0.27	1.31	0.45	0.60	5.47E-01	0.51	1.67	0.45	1.13	2.59E-01
13	B*37:01-KIR2DL1-m2	18	3	-0.94	0.39	0.58	-1.62	1.04E-01	-1.17	0.31	0.58	-2.02	4.36E-02
14	B*38:01-KIR2DL1-m1	22	17	-1.07	0.34	0.69	-1.55	1.22E-01	-0.25	0.78	0.69	-0.37	7.14E-01
15	B*39:01-KIR2DL1-m2	19	9	-0.05	0.95	0.34	-0.15	8.78E-01	0.50	1.65	0.34	1.48	1.39E-01
16	B*39:06-KIR2DL1-m2	19	13	0.11	1.12	0.28	0.40	6.91E-01	0.16	1.18	0.28	0.58	5.61E-01
17	B*40:01-KIR2DL1-m2	113	63	0.03	1.03	0.14	0.22	8.29E-01	0.09	1.10	0.14	0.68	4.98E-01
18	B*40:02-KIR2DL1-m2	24	4	-0.78	0.46	0.50	-1.56	1.20E-01	-0.08	0.92	0.51	-0.16	8.71E-01
19	B*41:01-KIR2DL1-m2	6	3	0.17	1.18	0.58	0.29	7.71E-01	0.30	1.35	0.58	0.52	6.05E-01
20	B*44:02-KIR2DL1-m2	130	61	-0.12	0.89	0.14	-0.84	4.03E-01	0.05	1.06	0.14	0.39	6.94E-01
21	B*44:03-KIR2DL1-m1	34	18	0.40	1.49	1.08	0.37	7.11E-01	0.82	2.27	1.08	0.76	4.50E-01
22	B*45:01-KIR2DL1-m2	12	3	-0.34	0.71	0.58	-0.59	5.56E-01	-0.65	0.52	0.58	-1.12	2.64E-01
23	B*49:01-KIR2DL1-m2	18	8	0.34	1.40	0.36	0.95	3.42E-01	0.22	1.24	0.36	0.61	5.44E-01
24	B*50:01-KIR2DL1-m2	19	10	0.24	1.26	0.32	0.73	4.63E-01	0.10	1.10	0.32	0.30	7.64E-01
25	B*51:01-KIR2DL1-m2	71	25	-0.34	0.71	0.21	-1.63	1.03E-01	-0.30	0.74	0.21	-1.47	1.42E-01

26	B*52:01-KIR2DL1-m2	9	2	-0.39	0.68	0.71	-0.54	5.86E-01	0.15	1.16	0.71	0.21	8.31E-01
27	B*53:01-KIR2DL1-m2	8	4	0.31	1.36	0.50	0.61	5.39E-01	0.27	1.30	0.50	0.53	5.99E-01
28	B*55:01-KIR2DL1-m2	27	13	0.04	1.05	0.28	0.16	8.75E-01	0.09	1.09	0.28	0.30	7.61E-01
29	B*56:01-KIR2DL1-m2	12	4	-0.04	0.96	0.50	-0.07	9.43E-01	0.76	2.14	0.50	1.51	1.32E-01
30	B*57:01-KIR2DL1-m2	15	6	-0.11	0.89	0.41	-0.28	7.83E-01	0.05	1.05	0.41	0.12	9.03E-01
31	B*58:01-KIR2DL1-m2	7	4	0.15	1.16	0.50	0.29	7.69E-01	0.16	1.18	0.50	0.32	7.48E-01
32	R10-KIR2DL1-m2	91	40	-0.14	0.87	0.17	-0.83	4.07E-01	-0.14	0.87	0.17	-0.82	4.12E-01
33	B*07:02-KIR2DL2-m2	45	23	-0.12	0.89	0.21	-0.56	5.72E-01	-0.24	0.78	0.22	-1.14	2.55E-01
34	B*08:01-KIR2DL2-m2	129	79	0.16	1.17	0.13	1.23	2.17E-01	0.07	1.07	0.13	0.53	5.97E-01
35	B*13:02-KIR2DL2-m1	9	2	-1.28	0.28	0.82	-1.56	1.19E-01	-1.17	0.31	0.82	-1.42	1.56E-01
36	B*14:01-KIR2DL2-m1	7	2	-1.78	0.17	0.92	-1.94	5.26E-02	-1.13	0.32	0.92	-1.23	2.19E-01
37	B*14:02-KIR2DL2-m1	17	4	-0.23	0.79	0.59	-0.39	6.94E-01	0.12	1.13	0.59	0.21	8.35E-01
38	B*15:01-KIR2DL2-m1	93	52	0.22	1.25	0.23	0.96	3.36E-01	0.28	1.32	0.23	1.20	2.31E-01
39	B*15:18-KIR2DL2-m1	7	1	-2.29	0.10	1.16	-1.97	4.87E-02	-1.55	0.21	1.16	-1.33	1.82E-01
40	B*18:01-KIR2DL2-m1	37	30	-0.01	0.99	0.27	-0.05	9.58E-01	-0.14	0.87	0.27	-0.52	6.05E-01
41	B*27:05-KIR2DL2-m1	28	9	-0.60	0.55	0.45	-1.34	1.81E-01	-0.59	0.55	0.45	-1.33	1.83E-01
42	B*35:01-KIR2DL2-m1	26	14	-0.37	0.69	0.38	-0.98	3.26E-01	-0.25	0.78	0.38	-0.65	5.19E-01
43	B*35:03-KIR2DL2-m2	8	10	0.41	1.51	0.32	1.29	1.97E-01	0.12	1.13	0.32	0.36	7.15E-01
44	B*35:08-KIR2DL2-m2	1	3	0.56	1.74	0.58	0.96	3.37E-01	0.56	1.76	0.58	0.97	3.30E-01
45	B*37:01-KIR2DL2-m2	11	1	-1.58	0.21	1.00	-1.58	1.14E-01	-1.62	0.20	1.00	-1.62	1.05E-01
46	B*38:01-KIR2DL2-m1	14	12	0.22	1.25	0.45	0.49	6.24E-01	0.24	1.27	0.46	0.52	6.04E-01
47	B*39:01-KIR2DL2-m2	10	6	0.34	1.40	0.41	0.82	4.10E-01	0.85	2.35	0.41	2.06	3.95E-02
48	B*39:06-KIR2DL2-m1	12	6	-0.53	0.59	0.55	-0.97	3.32E-01	-0.34	0.71	0.55	-0.61	5.41E-01
49	B*40:01-KIR2DL2-m2	56	27	-0.06	0.94	0.20	-0.29	7.73E-01	0.05	1.06	0.20	0.27	7.86E-01
50	B*40:02-KIR2DL2-m1	11	1	-0.79	0.45	1.16	-0.68	4.96E-01	-0.57	0.57	1.16	-0.49	6.23E-01
51	B*41:01-KIR2DL2-m1	6	1	-1.85	0.16	1.23	-1.50	1.33E-01	-1.19	0.30	1.23	-0.97	3.32E-01
52	B*44:02-KIR2DL2-m2	62	34	0.03	1.03	0.18	0.16	8.77E-01	0.07	1.08	0.18	0.42	6.78E-01
53	B*44:03-KIR2DL2-m1	18	12	0.86	2.37	0.49	1.77	7.69E-02	0.74	2.09	0.49	1.51	1.30E-01
54	B*45:01-KIR2DL2-m1	7	2	-0.38	0.68	1.01	-0.38	7.03E-01	-1.89	0.15	1.01	-1.88	6.03E-02
55	B*49:01-KIR2DL2-m2	8	3	0.72	2.06	0.58	1.24	2.15E-01	0.33	1.39	0.58	0.57	5.70E-01
56	B*50:01-KIR2DL2-m2	13	6	0.31	1.36	0.41	0.75	4.54E-01	0.05	1.05	0.41	0.11	9.09E-01
57	B*51:01-KIR2DL2-m2	35	13	-0.23	0.79	0.28	-0.83	4.07E-01	-0.22	0.80	0.28	-0.78	4.35E-01

58	B*52:01-KIR2DL2-m2	8	2	-0.33	0.72	0.71	-0.46	6.44E-01	0.42	1.53	0.71	0.59	5.53E-01
59	B*53:01-KIR2DL2-m2	5	3	0.34	1.41	0.58	0.59	5.54E-01	0.54	1.72	0.58	0.93	3.53E-01
60	B*55:01-KIR2DL2-m1	16	5	-0.81	0.44	0.58	-1.40	1.60E-01	-0.42	0.66	0.58	-0.73	4.68E-01
61	B*56:01-KIR2DL2-m2	5	1	-0.15	0.86	1.00	-0.15	8.82E-01	1.36	3.89	1.01	1.35	1.77E-01
62	B*57:01-KIR2DL2-m1	7	2	-0.74	0.48	0.87	-0.85	3.94E-01	-0.77	0.46	0.87	-0.88	3.77E-01
63	B*58:01-KIR2DL2-m1	6	2	-2.09	0.12	1.01	-2.07	3.81E-02	-3.76	0.02	1.02	-3.69	2.25E-04
64	R10-KIR2DL2-m2	47	20	-0.03	0.97	0.23	-0.15	8.81E-01	-0.14	0.87	0.23	-0.59	5.56E-01
65	B*07:02-KIR2DL3-m1	96	41	-0.53	0.59	0.51	-1.05	2.94E-01	-1.36	0.26	0.51	-2.66	7.76E-03
66	B*08:01-KIR2DL3-m1	243	128	-0.06	0.94	0.36	-0.17	8.65E-01	0.13	1.14	0.36	0.36	7.22E-01
67	B*13:02-KIR2DL3-m2	20	7	-0.33	0.72	0.38	-0.87	3.83E-01	0.35	1.42	0.38	0.91	3.65E-01
68	B*14:01-KIR2DL3-m2	11	5	-0.09	0.92	0.45	-0.20	8.45E-01	-0.32	0.73	0.45	-0.70	4.82E-01
69	B*14:02-KIR2DL3-m1	28	13	-0.17	0.85	0.78	-0.21	8.30E-01	-0.29	0.75	0.78	-0.37	7.14E-01
70	B*15:01-KIR2DL3-m1	193	91	-0.47	0.63	0.39	-1.22	2.24E-01	-0.69	0.50	0.39	-1.78	7.56E-02
71	B*15:18-KIR2DL3-m2	6	4	0.74	2.11	0.50	1.48	1.39E-01	1.03	2.80	0.50	2.05	4.07E-02
72	B*18:01-KIR2DL3-m1	77	56	-0.30	0.74	0.42	-0.71	4.80E-01	-0.59	0.56	0.42	-1.39	1.65E-01
73	B*27:05-KIR2DL3-m1	46	19	-1.15	0.32	0.65	-1.78	7.53E-02	-0.94	0.39	0.65	-1.46	1.45E-01
74	B*35:01-KIR2DL3-m2	45	30	-0.02	0.98	0.19	-0.09	9.31E-01	0.07	1.07	0.19	0.36	7.19E-01
75	B*35:03-KIR2DL3-m1	14	12	0.14	1.15	0.78	0.18	8.57E-01	0.59	1.80	0.79	0.75	4.53E-01
76	B*35:08-KIR2DL3-m2	6	5	0.27	1.31	0.45	0.60	5.47E-01	0.51	1.67	0.45	1.13	2.59E-01
77	B*37:01-KIR2DL3-m2	16	3	-0.76	0.47	0.58	-1.32	1.88E-01	-0.95	0.39	0.58	-1.63	1.03E-01
78	B*38:01-KIR2DL3-m1	18	15	-0.07	0.93	0.52	-0.13	8.93E-01	0.40	1.48	0.52	0.76	4.46E-01
79	B*39:01-KIR2DL3-m2	17	8	-0.07	0.94	0.36	-0.18	8.54E-01	0.48	1.62	0.36	1.33	1.83E-01
80	B*39:06-KIR2DL3-m1	18	11	-0.21	0.81	0.67	-0.31	7.57E-01	-0.54	0.58	0.68	-0.80	4.24E-01
81	B*40:01-KIR2DL3-m2	107	59	0.03	1.03	0.14	0.23	8.20E-01	0.10	1.10	0.14	0.70	4.82E-01
82	B*40:02-KIR2DL3-m2	21	4	-0.66	0.52	0.50	-1.31	1.90E-01	0.01	1.01	0.51	0.01	9.91E-01
83	B*41:01-KIR2DL3-m1	5	2	0.69	1.99	1.24	0.56	5.79E-01	0.06	1.06	1.24	0.05	9.61E-01
84	B*44:02-KIR2DL3-m1	121	59	0.92	2.51	0.62	1.49	1.36E-01	1.04	2.82	0.62	1.68	9.37E-02
85	B*44:03-KIR2DL3-m1	29	18	1.15	3.16	1.04	1.10	2.69E-01	1.29	3.64	1.04	1.24	2.15E-01
86	B*45:01-KIR2DL3-m2	12	3	-0.34	0.71	0.58	-0.59	5.56E-01	-0.65	0.52	0.58	-1.12	2.64E-01
87	B*49:01-KIR2DL3-m2	18	8	0.34	1.40	0.36	0.95	3.42E-01	0.22	1.24	0.36	0.61	5.44E-01
88	B*50:01-KIR2DL3-m1	18	9	0.23	1.25	0.80	0.28	7.79E-01	0.11	1.12	0.80	0.14	8.87E-01
89	B*51:01-KIR2DL3-m2	68	23	-0.36	0.70	0.21	-1.67	9.42E-02	-0.31	0.73	0.21	-1.44	1.49E-01

90	B*52:01-KIR2DL3-m2	9	1	-1.08	0.34	1.00	-1.08	2.82E-01	-0.51	0.60	1.00	-0.51	6.11E-01
91	B*53:01-KIR2DL3-m2	7	4	0.34	1.40	0.50	0.67	5.05E-01	0.28	1.32	0.50	0.55	5.79E-01
92	B*55:01-KIR2DL3-m2	24	13	0.14	1.15	0.28	0.49	6.21E-01	0.24	1.28	0.28	0.86	3.90E-01
93	B*56:01-KIR2DL3-m2	11	4	0.11	1.12	0.50	0.22	8.23E-01	0.79	2.21	0.50	1.57	1.16E-01
94	B*57:01-KIR2DL3-m2	14	6	-0.11	0.90	0.41	-0.26	7.91E-01	0.05	1.05	0.41	0.12	9.03E-01
95	B*58:01-KIR2DL3-m2	6	4	0.28	1.32	0.50	0.55	5.84E-01	0.20	1.22	0.51	0.39	6.94E-01
96	R10-KIR2DL3-m2	84	36	-0.16	0.85	0.17	-0.92	3.58E-01	-0.18	0.84	0.17	-1.03	3.03E-01
97	B*07:02-KIR2DL4-m2	103	46	-0.14	0.87	0.16	-0.87	3.85E-01	-0.21	0.81	0.16	-1.35	1.77E-01
98	B*08:01-KIR2DL4-m2	262	141	0.09	1.09	0.10	0.85	3.98E-01	0.05	1.05	0.10	0.46	6.43E-01
99	B*13:02-KIR2DL4-m2	21	8	-0.23	0.79	0.36	-0.65	5.18E-01	0.46	1.58	0.36	1.27	2.03E-01
100	B*14:01-KIR2DL4-m2	11	5	-0.09	0.92	0.45	-0.20	8.45E-01	-0.32	0.73	0.45	-0.70	4.82E-01
101	B*14:02-KIR2DL4-m2	34	15	0.06	1.06	0.26	0.21	8.31E-01	-0.04	0.96	0.26	-0.15	8.79E-01
102	B*15:01-KIR2DL4-m2	203	101	-0.02	0.98	0.11	-0.21	8.38E-01	-0.15	0.86	0.11	-1.28	2.02E-01
103	B*15:18-KIR2DL4-m2	8	4	0.45	1.57	0.50	0.89	3.73E-01	0.77	2.16	0.50	1.53	1.26E-01
104	B*18:01-KIR2DL4-m2	83	64	0.40	1.49	0.14	2.93	3.35E-03	0.16	1.18	0.14	1.20	2.30E-01
105	B*27:05-KIR2DL4-m2	47	22	-0.15	0.86	0.22	-0.68	4.99E-01	-0.10	0.91	0.22	-0.45	6.50E-01
106	B*35:01-KIR2DL4-m2	48	30	-0.06	0.94	0.19	-0.30	7.65E-01	0.04	1.04	0.19	0.22	8.25E-01
107	B*35:03-KIR2DL4-m2	17	14	0.14	1.15	0.27	0.52	6.06E-01	0.07	1.07	0.27	0.25	8.06E-01
108	B*35:08-KIR2DL4-m2	6	5	0.27	1.31	0.45	0.60	5.47E-01	0.51	1.67	0.45	1.13	2.59E-01
109	B*37:01-KIR2DL4-m2	19	3	-1.01	0.36	0.58	-1.74	8.13E-02	-1.25	0.29	0.58	-2.16	3.11E-02
110	B*38:01-KIR2DL4-m2	22	21	0.22	1.24	0.22	0.97	3.31E-01	0.00	1.00	0.23	0.00	9.98E-01
111	B*39:01-KIR2DL4-m2	19	9	-0.05	0.95	0.34	-0.15	8.78E-01	0.50	1.65	0.34	1.48	1.39E-01
112	B*39:06-KIR2DL4-m2	20	14	0.12	1.12	0.27	0.43	6.66E-01	0.22	1.25	0.27	0.81	4.20E-01
113	B*40:01-KIR2DL4-m2	114	63	0.02	1.02	0.14	0.12	9.05E-01	0.09	1.10	0.14	0.67	5.02E-01
114	B*40:02-KIR2DL4-m2	24	4	-0.78	0.46	0.50	-1.56	1.20E-01	-0.08	0.92	0.51	-0.16	8.71E-01
115	B*41:01-KIR2DL4-m2	8	3	-0.10	0.90	0.58	-0.18	8.58E-01	0.04	1.04	0.58	0.07	9.46E-01
116	B*44:02-KIR2DL4-m2	134	62	-0.12	0.89	0.14	-0.88	3.78E-01	0.06	1.06	0.14	0.42	6.77E-01
117	B*44:03-KIR2DL4-m2	37	19	0.03	1.03	0.24	0.13	8.98E-01	0.10	1.10	0.24	0.41	6.80E-01
118	B*45:01-KIR2DL4-m2	13	4	-0.14	0.87	0.50	-0.27	7.88E-01	-0.45	0.64	0.50	-0.90	3.68E-01
119	B*49:01-KIR2DL4-m2	19	8	0.32	1.38	0.36	0.89	3.73E-01	0.21	1.23	0.36	0.58	5.59E-01
120	B*50:01-KIR2DL4-m2	20	11	0.24	1.27	0.31	0.78	4.34E-01	0.03	1.03	0.31	0.11	9.12E-01
121	B*51:01-KIR2DL4-m2	75	25	-0.35	0.70	0.21	-1.72	8.59E-02	-0.31	0.73	0.21	-1.50	1.34E-01

122	B*52:01-KIR2DL4-m2	10	2	-0.50	0.61	0.71	-0.70	4.82E-01	0.11	1.12	0.71	0.16	8.73E-01
123	B*53:01-KIR2DL4-m2	8	4	0.31	1.36	0.50	0.61	5.39E-01	0.27	1.30	0.50	0.53	5.99E-01
124	B*55:01-KIR2DL4-m2	28	13	-0.02	0.98	0.28	-0.07	9.41E-01	0.03	1.03	0.28	0.11	9.16E-01
125	B*56:01-KIR2DL4-m2	12	4	-0.04	0.96	0.50	-0.07	9.43E-01	0.76	2.14	0.50	1.51	1.32E-01
126	B*57:01-KIR2DL4-m2	15	6	-0.11	0.89	0.41	-0.28	7.83E-01	0.05	1.05	0.41	0.12	9.03E-01
127	B*58:01-KIR2DL4-m2	7	4	0.15	1.16	0.50	0.29	7.69E-01	0.16	1.18	0.50	0.32	7.48E-01
128	R10-KIR2DL4-m2	92	40	-0.14	0.87	0.17	-0.84	4.02E-01	-0.14	0.87	0.17	-0.83	4.09E-01
129	B*07:02-KIR2DL5-m2	41	22	0.03	1.03	0.22	0.15	8.83E-01	-0.12	0.89	0.22	-0.53	5.96E-01
130	B*08:01-KIR2DL5-m2	125	76	0.21	1.23	0.13	1.63	1.02E-01	0.15	1.17	0.13	1.21	2.28E-01
131	B*13:02-KIR2DL5-m1	10	4	-0.39	0.68	0.71	-0.55	5.83E-01	-0.49	0.61	0.71	-0.69	4.91E-01
132	B*14:01-KIR2DL5-m1	6	2	-1.24	0.29	0.92	-1.35	1.77E-01	-1.00	0.37	0.92	-1.08	2.78E-01
133	B*14:02-KIR2DL5-m1	14	5	0.24	1.27	0.56	0.43	6.66E-01	0.28	1.33	0.56	0.51	6.11E-01
134	B*15:01-KIR2DL5-m2	85	53	0.18	1.20	0.15	1.25	2.10E-01	-0.06	0.95	0.15	-0.38	7.07E-01
135	B*15:18-KIR2DL5-m1	5	1	-1.68	0.19	1.16	-1.45	1.48E-01	-0.80	0.45	1.16	-0.69	4.89E-01
136	B*18:01-KIR2DL5-m2	38	31	0.41	1.51	0.19	2.20	2.80E-02	0.18	1.20	0.19	0.97	3.34E-01
137	B*27:05-KIR2DL5-m2	22	10	-0.18	0.83	0.32	-0.57	5.69E-01	-0.15	0.86	0.32	-0.47	6.41E-01
138	B*35:01-KIR2DL5-m1	23	18	0.12	1.13	0.39	0.31	7.57E-01	-0.12	0.88	0.39	-0.32	7.51E-01
139	B*35:03-KIR2DL5-m2	9	8	0.24	1.27	0.36	0.66	5.08E-01	0.23	1.26	0.36	0.65	5.13E-01
140	B*35:08-KIR2DL5-m1	4	1	-1.38	0.25	1.12	-1.23	2.19E-01	-0.60	0.55	1.12	-0.53	5.96E-01
141	B*37:01-KIR2DL5-m2	11	1	-1.53	0.22	1.00	-1.53	1.27E-01	-1.44	0.24	1.00	-1.44	1.51E-01
142	B*38:01-KIR2DL5-m1	12	9	-0.42	0.65	0.45	-0.94	3.50E-01	-0.58	0.56	0.45	-1.27	2.04E-01
143	B*39:01-KIR2DL5-m2	10	6	0.29	1.34	0.41	0.71	4.76E-01	0.72	2.06	0.41	1.75	8.04E-02
144	B*39:06-KIR2DL5-m1	10	7	-0.49	0.61	0.54	-0.90	3.69E-01	-0.45	0.64	0.55	-0.81	4.15E-01
145	B*40:01-KIR2DL5-m1	63	28	-0.29	0.75	0.28	-1.05	2.93E-01	-0.15	0.86	0.28	-0.56	5.77E-01
146	B*40:02-KIR2DL5-m1	9	3	0.99	2.68	1.16	0.85	3.94E-01	0.93	2.53	1.16	0.80	4.24E-01
147	B*41:01-KIR2DL5-m1	2	2	1.28	3.60	1.23	1.04	2.98E-01	1.28	3.61	1.23	1.04	2.97E-01
148	B*44:02-KIR2DL5-m1	70	28	-0.56	0.57	0.28	-2.00	4.57E-02	-0.26	0.77	0.28	-0.92	3.57E-01
149	B*44:03-KIR2DL5-m1	20	8	-0.55	0.58	0.48	-1.15	2.49E-01	-0.22	0.80	0.48	-0.46	6.42E-01
150	B*45:01-KIR2DL5-m2	6	1	-0.78	0.46	1.00	-0.78	4.35E-01	-1.21	0.30	1.00	-1.21	2.26E-01
151	B*49:01-KIR2DL5-m1	10	4	-0.25	0.78	0.71	-0.35	7.28E-01	-0.44	0.65	0.72	-0.61	5.42E-01
152	B*50:01-KIR2DL5-m2	9	6	0.58	1.78	0.41	1.40	1.61E-01	0.19	1.22	0.41	0.47	6.38E-01
153	B*51:01-KIR2DL5-m1	33	16	0.74	2.09	0.43	1.72	8.50E-02	0.68	1.97	0.43	1.57	1.15E-01

154	B*52:01-KIR2DL5-m2	7	2	-0.36	0.70	0.71	-0.50	6.15E-01	0.14	1.15	0.71	0.20	8.44E-01
155	B*53:01-KIR2DL5-m2	4	4	0.55	1.73	0.50	1.09	2.77E-01	0.66	1.93	0.50	1.31	1.91E-01
156	B*55:01-KIR2DL5-m1	17	5	-0.76	0.47	0.58	-1.31	1.90E-01	-0.95	0.39	0.58	-1.64	1.00E-01
157	B*56:01-KIR2DL5-m1	7	1	-1.11	0.33	1.16	-0.96	3.37E-01	-1.38	0.25	1.16	-1.19	2.36E-01
158	B*57:01-KIR2DL5-m1	8	2	-0.27	0.76	0.87	-0.31	7.53E-01	-0.37	0.69	0.87	-0.43	6.69E-01
159	B*58:01-KIR2DL5-m1	5	1	-0.90	0.41	1.16	-0.77	4.39E-01	-2.36	0.09	1.17	-2.02	4.33E-02
160	R10-KIR2DL5-m1	52	27	0.23	1.25	0.35	0.64	5.23E-01	0.44	1.55	0.35	1.24	2.17E-01
161	B*07:02-KIR2DS1-m1	29	17	0.39	1.47	0.32	1.19	2.33E-01	0.15	1.17	0.32	0.47	6.37E-01
162	B*08:01-KIR2DS1-m2	103	61	0.15	1.17	0.14	1.10	2.72E-01	0.11	1.12	0.14	0.81	4.17E-01
163	B*13:02-KIR2DS1-m2	7	3	-0.22	0.80	0.58	-0.38	7.02E-01	0.45	1.56	0.58	0.77	4.43E-01
164	B*14:01-KIR2DS1-m1	3	2	0.14	1.15	0.92	0.15	8.78E-01	0.97	2.64	0.92	1.05	2.93E-01
165	B*14:02-KIR2DS1-m1	10	5	0.44	1.56	0.56	0.79	4.27E-01	0.36	1.43	0.56	0.64	5.19E-01
166	B*15:01-KIR2DS1-m2	71	45	0.25	1.28	0.16	1.58	1.14E-01	-0.03	0.97	0.16	-0.20	8.44E-01
167	B*15:18-KIR2DS1-m2	3	1	-0.06	0.94	1.00	-0.06	9.51E-01	0.70	2.01	1.00	0.70	4.87E-01
168	B*18:01-KIR2DS1-m2	29	24	0.39	1.48	0.21	1.86	6.35E-02	0.16	1.17	0.21	0.74	4.62E-01
169	B*27:05-KIR2DS1-m1	15	9	0.21	1.23	0.45	0.47	6.37E-01	0.19	1.21	0.45	0.42	6.74E-01
170	B*35:01-KIR2DS1-m1	18	16	0.24	1.27	0.38	0.63	5.27E-01	-0.20	0.82	0.38	-0.53	5.95E-01
171	B*35:03-KIR2DS1-m1	8	5	-0.64	0.53	0.57	-1.12	2.63E-01	-0.35	0.70	0.57	-0.61	5.39E-01
172	B*35:08-KIR2DS1-m1	4	1	-1.44	0.24	1.12	-1.28	2.00E-01	-0.64	0.53	1.12	-0.57	5.71E-01
173	B*37:01-KIR2DS1-m2	9	1	-1.22	0.30	1.00	-1.22	2.24E-01	-0.85	0.43	1.00	-0.85	3.97E-01
174	B*38:01-KIR2DS1-m1	9	8	-0.37	0.69	0.46	-0.80	4.22E-01	-0.66	0.52	0.46	-1.42	1.55E-01
175	B*39:01-KIR2DS1-m2	4	4	1.05	2.86	0.50	2.09	3.68E-02	1.37	3.92	0.50	2.71	6.75E-03
176	B*39:06-KIR2DS1-m2	7	6	0.11	1.12	0.41	0.27	7.86E-01	0.42	1.52	0.41	1.01	3.13E-01
177	B*40:01-KIR2DS1-m2	43	23	0.09	1.10	0.21	0.43	6.70E-01	0.18	1.19	0.21	0.82	4.13E-01
178	B*40:02-KIR2DS1-m1	5	2	1.42	4.13	1.01	1.41	1.58E-01	1.09	2.97	1.01	1.08	2.79E-01
179	B*41:01-KIR2DS1-m2	1	1	1.02	2.79	1.00	1.02	3.06E-01	0.28	1.32	1.00	0.28	7.82E-01
180	B*44:02-KIR2DS1-m2	53	24	-0.19	0.83	0.21	-0.90	3.70E-01	0.21	1.24	0.21	1.00	3.16E-01
181	B*44:03-KIR2DS1-m1	16	6	-0.71	0.49	0.50	-1.40	1.62E-01	-0.22	0.81	0.51	-0.42	6.72E-01
182	B*45:01-KIR2DS1-m2	5	0										
183	B*49:01-KIR2DS1-m2	6	4	0.39	1.48	0.50	0.77	4.39E-01	0.34	1.40	0.50	0.67	5.05E-01
184	B*50:01-KIR2DS1-m2	10	6	0.54	1.71	0.41	1.30	1.94E-01	0.18	1.20	0.41	0.44	6.57E-01
185	B*51:01-KIR2DS1-m2	28	10	-0.19	0.83	0.32	-0.60	5.51E-01	-0.14	0.87	0.32	-0.45	6.54E-01

186	B*52:01-KIR2DS1-m1	5	1	-0.17	0.84	1.42	-0.12	9.05E-01	-0.18	0.83	1.42	-0.13	8.98E-01
187	B*53:01-KIR2DS1-m2	2	3	1.06	2.89	0.58	1.83	6.70E-02	0.66	1.93	0.58	1.13	2.58E-01
188	B*55:01-KIR2DS1-m1	14	3	-1.18	0.31	0.67	-1.77	7.63E-02	-1.34	0.26	0.67	-2.01	4.41E-02
189	B*56:01-KIR2DS1-m1	7	1	-1.41	0.25	1.16	-1.21	2.26E-01	-1.62	0.20	1.16	-1.39	1.63E-01
190	B*57:01-KIR2DS1-m1	7	2	-0.29	0.75	0.87	-0.34	7.36E-01	-0.42	0.66	0.87	-0.48	6.33E-01
191	B*58:01-KIR2DS1-m1	3	1	0.08	1.08	1.16	0.07	9.47E-01	-1.21	0.30	1.17	-1.03	3.01E-01
192	R10-KIR2DS1-m2	37	20	-0.15	0.86	0.23	-0.64	5.21E-01	-0.07	0.93	0.23	-0.31	7.53E-01
193	B*07:02-KIR2DS2-m2	44	23	-0.10	0.90	0.21	-0.47	6.36E-01	-0.22	0.80	0.22	-1.04	2.96E-01
194	B*08:01-KIR2DS2-m2	132	82	0.18	1.20	0.12	1.47	1.41E-01	0.09	1.10	0.12	0.77	4.44E-01
195	B*13:02-KIR2DS2-m1	9	2	-1.31	0.27	0.82	-1.59	1.13E-01	-1.20	0.30	0.82	-1.46	1.45E-01
196	B*14:01-KIR2DS2-m1	7	2	-1.81	0.16	0.92	-1.96	4.97E-02	-1.16	0.31	0.92	-1.26	2.08E-01
197	B*14:02-KIR2DS2-m1	17	4	-0.26	0.77	0.59	-0.43	6.66E-01	0.09	1.10	0.59	0.16	8.75E-01
198	B*15:01-KIR2DS2-m1	94	52	0.18	1.20	0.23	0.78	4.36E-01	0.23	1.26	0.23	1.00	3.18E-01
199	B*15:18-KIR2DS2-m1	7	1	-2.31	0.10	1.16	-1.99	4.66E-02	-1.58	0.21	1.16	-1.36	1.74E-01
200	B*18:01-KIR2DS2-m2	37	32	0.44	1.56	0.18	2.41	1.59E-02	0.14	1.15	0.18	0.74	4.59E-01
201	B*27:05-KIR2DS2-m1	29	9	-0.73	0.48	0.45	-1.64	1.02E-01	-0.65	0.52	0.45	-1.46	1.44E-01
202	B*35:01-KIR2DS2-m1	26	15	-0.28	0.75	0.38	-0.74	4.58E-01	-0.17	0.85	0.38	-0.44	6.60E-01
203	B*35:03-KIR2DS2-m2	8	10	0.41	1.51	0.32	1.29	1.97E-01	0.12	1.13	0.32	0.36	7.15E-01
204	B*35:08-KIR2DS2-m2	1	3	0.56	1.74	0.58	0.96	3.37E-01	0.56	1.76	0.58	0.97	3.30E-01
205	B*37:01-KIR2DS2-m2	11	1	-1.58	0.21	1.00	-1.58	1.14E-01	-1.62	0.20	1.00	-1.62	1.05E-01
206	B*38:01-KIR2DS2-m1	15	12	0.12	1.13	0.45	0.26	7.92E-01	0.17	1.18	0.46	0.37	7.11E-01
207	B*39:01-KIR2DS2-m2	10	6	0.34	1.40	0.41	0.82	4.10E-01	0.85	2.35	0.41	2.06	3.95E-02
208	B*39:06-KIR2DS2-m1	12	6	-0.56	0.57	0.55	-1.01	3.11E-01	-0.37	0.69	0.55	-0.67	5.05E-01
209	B*40:01-KIR2DS2-m2	56	28	-0.03	0.97	0.20	-0.13	8.93E-01	0.09	1.09	0.20	0.45	6.55E-01
210	B*40:02-KIR2DS2-m1	10	1	-0.71	0.49	1.16	-0.61	5.39E-01	-0.46	0.63	1.16	-0.40	6.90E-01
211	B*41:01-KIR2DS2-m1	6	1	-1.87	0.15	1.23	-1.52	1.28E-01	-1.22	0.29	1.23	-0.99	3.20E-01
212	B*44:02-KIR2DS2-m2	63	34	0.01	1.01	0.18	0.07	9.47E-01	0.07	1.07	0.18	0.40	6.89E-01
213	B*44:03-KIR2DS2-m1	19	12	0.71	2.04	0.49	1.46	1.43E-01	0.59	1.81	0.49	1.21	2.25E-01
214	B*45:01-KIR2DS2-m1	7	2	-0.41	0.67	1.01	-0.40	6.86E-01	-1.92	0.15	1.01	-1.91	5.64E-02
215	B*49:01-KIR2DS2-m2	8	3	0.72	2.06	0.58	1.24	2.15E-01	0.33	1.39	0.58	0.57	5.70E-01
216	B*50:01-KIR2DS2-m2	13	6	0.31	1.36	0.41	0.75	4.54E-01	0.05	1.05	0.41	0.11	9.09E-01
217	B*51:01-KIR2DS2-m2	34	13	-0.20	0.82	0.28	-0.72	4.71E-01	-0.19	0.83	0.28	-0.66	5.11E-01

218	B*52:01-KIR2DS2-m2	8	2	-0.33	0.72	0.71	-0.46	6.44E-01	0.42	1.53	0.71	0.59	5.53E-01
219	B*53:01-KIR2DS2-m2	5	3	0.34	1.41	0.58	0.59	5.54E-01	0.54	1.72	0.58	0.93	3.53E-01
220	B*55:01-KIR2DS2-m1	16	5	-0.84	0.43	0.58	-1.44	1.49E-01	-0.45	0.64	0.58	-0.78	4.36E-01
221	B*56:01-KIR2DS2-m2	5	1	-0.15	0.86	1.00	-0.15	8.82E-01	1.36	3.89	1.01	1.35	1.77E-01
222	B*57:01-KIR2DS2-m1	7	2	-0.77	0.46	0.87	-0.88	3.79E-01	-0.80	0.45	0.87	-0.92	3.59E-01
223	B*58:01-KIR2DS2-m1	6	2	-2.11	0.12	1.01	-2.10	3.61E-02	-3.78	0.02	1.02	-3.72	2.01E-04
224	R10-KIR2DS2-m2	48	20	-0.07	0.93	0.23	-0.29	7.68E-01	-0.16	0.85	0.23	-0.69	4.88E-01
225	B*07:02-KIR2DS3-m1	21	14	0.34	1.41	0.34	1.00	3.15E-01	0.43	1.54	0.34	1.25	2.10E-01
226	B*08:01-KIR2DS3-m2	63	40	0.21	1.23	0.17	1.23	2.18E-01	0.08	1.08	0.17	0.47	6.41E-01
227	B*13:02-KIR2DS3-m1	6	1	-1.31	0.27	1.08	-1.22	2.24E-01	-1.37	0.25	1.08	-1.27	2.03E-01
228	B*14:01-KIR2DS3-m2	6	0										
229	B*14:02-KIR2DS3-m1	8	3	0.48	1.62	0.66	0.73	4.63E-01	0.72	2.05	0.66	1.10	2.73E-01
230	B*15:01-KIR2DS3-m1	38	30	0.51	1.67	0.25	2.03	4.23E-02	0.36	1.43	0.25	1.42	1.55E-01
231	B*15:18-KIR2DS3-m2	3	0										
232	B*18:01-KIR2DS3-m2	17	15	0.29	1.33	0.26	1.09	2.78E-01	0.12	1.12	0.26	0.44	6.61E-01
233	B*27:05-KIR2DS3-m2	16	7	-0.33	0.72	0.38	-0.86	3.91E-01	-0.23	0.79	0.38	-0.61	5.45E-01
234	B*35:01-KIR2DS3-m1	15	7	-0.50	0.61	0.45	-1.12	2.64E-01	-0.24	0.79	0.45	-0.53	5.93E-01
235	B*35:03-KIR2DS3-m2	6	7	0.28	1.32	0.38	0.73	4.63E-01	0.40	1.49	0.38	1.05	2.93E-01
236	B*35:08-KIR2DS3-m2	1	0										
237	B*37:01-KIR2DS3-m2	6	0										
238	B*38:01-KIR2DS3-m1	9	5	-0.44	0.64	0.53	-0.84	4.01E-01	-0.74	0.48	0.53	-1.41	1.58E-01
239	B*39:01-KIR2DS3-m2	8	4	0.17	1.19	0.50	0.34	7.31E-01	0.64	1.90	0.50	1.28	2.02E-01
240	B*39:06-KIR2DS3-m1	7	4	-0.61	0.54	0.60	-1.01	3.13E-01	-0.72	0.49	0.60	-1.19	2.35E-01
241	B*40:01-KIR2DS3-m2	38	17	-0.08	0.92	0.25	-0.32	7.48E-01	0.08	1.08	0.25	0.31	7.55E-01
242	B*40:02-KIR2DS3-m2	6	1	-1.10	0.33	1.00	-1.09	2.74E-01	-0.17	0.84	1.00	-0.17	8.62E-01
243	B*41:01-KIR2DS3-m2	1	1	0.24	1.27	1.00	0.24	8.12E-01	1.46	4.31	1.01	1.45	1.47E-01
244	B*44:02-KIR2DS3-m1	32	14	-0.39	0.68	0.33	-1.20	2.31E-01	-0.39	0.68	0.33	-1.18	2.38E-01
245	B*44:03-KIR2DS3-m1	12	4	-0.30	0.74	0.57	-0.52	6.04E-01	-0.17	0.85	0.57	-0.29	7.71E-01
246	B*45:01-KIR2DS3-m2	1	1	0.90	2.46	1.00	0.90	3.69E-01	-0.45	0.63	1.00	-0.45	6.51E-01
247	B*49:01-KIR2DS3-m1	3	2	0.73	2.08	0.83	0.89	3.73E-01	-0.19	0.82	0.83	-0.23	8.16E-01
248	B*50:01-KIR2DS3-m2	5	2	0.19	1.21	0.71	0.27	7.91E-01	1.02	2.77	0.71	1.43	1.52E-01
249	B*51:01-KIR2DS3-m1	14	12	1.17	3.23	0.42	2.81	4.97E-03	0.84	2.32	0.42	2.02	4.33E-02

250	B*52:01-KIR2DS3-m2	4	2	-0.05	0.95	0.71	-0.08	9.39E-01	0.50	1.64	0.71	0.70	4.87E-01
251	B*53:01-KIR2DS3-m2	3	1	-0.25	0.78	1.00	-0.25	8.03E-01	0.66	1.93	1.00	0.66	5.12E-01
252	B*55:01-KIR2DS3-m1	10	4	-0.20	0.82	0.61	-0.33	7.43E-01	-0.39	0.68	0.61	-0.63	5.28E-01
253	B*56:01-KIR2DS3-m2	3	0										
254	B*57:01-KIR2DS3-m1	4	1	0.04	1.04	1.10	0.03	9.72E-01	-0.28	0.76	1.10	-0.25	8.01E-01
255	B*58:01-KIR2DS3-m2	3	0										
256	R10-KIR2DS3-m1	24	14	0.39	1.48	0.35	1.12	2.63E-01	0.50	1.64	0.35	1.41	1.58E-01
257	B*07:02-KIR2DS4-m2	98	42	-0.18	0.83	0.16	-1.13	2.56E-01	-0.25	0.78	0.16	-1.56	1.20E-01
258	B*08:01-KIR2DS4-m2	247	135	0.09	1.09	0.11	0.85	3.95E-01	0.04	1.04	0.11	0.34	7.36E-01
259	B*13:02-KIR2DS4-m2	20	8	-0.19	0.83	0.36	-0.52	6.00E-01	0.48	1.61	0.36	1.33	1.85E-01
260	B*14:01-KIR2DS4-m2	11	5	-0.09	0.92	0.45	-0.20	8.45E-01	-0.32	0.73	0.45	-0.70	4.82E-01
261	B*14:02-KIR2DS4-m2	34	15	0.06	1.06	0.26	0.21	8.31E-01	-0.04	0.96	0.26	-0.15	8.79E-01
262	B*15:01-KIR2DS4-m2	195	92	-0.10	0.90	0.12	-0.85	3.98E-01	-0.20	0.82	0.12	-1.72	8.51E-02
263	B*15:18-KIR2DS4-m2	8	4	0.45	1.57	0.50	0.89	3.73E-01	0.77	2.16	0.50	1.53	1.26E-01
264	B*18:01-KIR2DS4-m2	81	63	0.40	1.50	0.14	2.93	3.37E-03	0.19	1.21	0.14	1.40	1.61E-01
265	B*27:05-KIR2DS4-m2	46	21	-0.18	0.84	0.22	-0.80	4.26E-01	-0.13	0.88	0.22	-0.57	5.68E-01
266	B*35:01-KIR2DS4-m1	45	26	-0.53	0.59	0.60	-0.89	3.75E-01	0.08	1.09	0.60	0.14	8.91E-01
267	B*35:03-KIR2DS4-m1	14	13	1.32	3.76	1.06	1.24	2.14E-01	0.98	2.66	1.07	0.92	3.58E-01
268	B*35:08-KIR2DS4-m2	5	5	0.32	1.38	0.45	0.71	4.76E-01	0.52	1.69	0.45	1.16	2.48E-01
269	B*37:01-KIR2DS4-m2	19	3	-1.01	0.36	0.58	-1.74	8.13E-02	-1.25	0.29	0.58	-2.16	3.11E-02
270	B*38:01-KIR2DS4-m2	22	21	0.22	1.24	0.22	0.97	3.31E-01	0.00	1.00	0.23	0.00	9.98E-01
271	B*39:01-KIR2DS4-m2	19	9	-0.05	0.95	0.34	-0.15	8.78E-01	0.50	1.65	0.34	1.48	1.39E-01
272	B*39:06-KIR2DS4-m2	20	13	0.04	1.05	0.28	0.16	8.76E-01	0.16	1.17	0.28	0.56	5.78E-01
273	B*40:01-KIR2DS4-m1	106	61	1.23	3.41	0.76	1.62	1.06E-01	0.92	2.50	0.76	1.21	2.28E-01
274	B*40:02-KIR2DS4-m2	23	4	-0.76	0.47	0.50	-1.51	1.32E-01	-0.08	0.92	0.51	-0.16	8.76E-01
275	B*41:01-KIR2DS4-m2	7	2	-0.40	0.67	0.71	-0.56	5.76E-01	-0.06	0.94	0.71	-0.09	9.31E-01
276	B*44:02-KIR2DS4-m1	125	61	1.76	5.79	1.04	1.70	8.99E-02	1.23	3.41	1.04	1.18	2.37E-01
277	B*44:03-KIR2DS4-m2	37	18	0.01	1.01	0.24	0.06	9.51E-01	0.06	1.06	0.24	0.26	7.97E-01
278	B*45:01-KIR2DS4-m2	12	4	-0.06	0.94	0.50	-0.12	9.08E-01	-0.34	0.72	0.50	-0.67	5.06E-01
279	B*49:01-KIR2DS4-m2	19	7	0.30	1.36	0.38	0.79	4.27E-01	0.23	1.26	0.38	0.61	5.42E-01
280	B*50:01-KIR2DS4-m1	18	9	0.01	1.01	0.82	0.01	9.90E-01	-0.57	0.57	0.82	-0.70	4.87E-01
281	B*51:01-KIR2DS4-m2	72	25	-0.34	0.71	0.21	-1.64	1.02E-01	-0.30	0.74	0.21	-1.48	1.40E-01

282	B*52:01-KIR2DS4-m2	10	2	-0.50	0.61	0.71	-0.70	4.82E-01	0.11	1.12	0.71	0.16	8.73E-01
283	B*53:01-KIR2DS4-m2	8	4	0.31	1.36	0.50	0.61	5.39E-01	0.27	1.30	0.50	0.53	5.99E-01
284	B*55:01-KIR2DS4-m2	28	13	-0.02	0.98	0.28	-0.07	9.41E-01	0.03	1.03	0.28	0.11	9.16E-01
285	B*56:01-KIR2DS4-m2	10	3	-0.22	0.80	0.58	-0.39	7.00E-01	0.56	1.75	0.58	0.96	3.36E-01
286	B*57:01-KIR2DS4-m2	15	6	-0.11	0.89	0.41	-0.28	7.83E-01	0.05	1.05	0.41	0.12	9.03E-01
287	B*58:01-KIR2DS4-m2	6	4	0.17	1.19	0.50	0.34	7.33E-01	0.17	1.19	0.51	0.34	7.37E-01
288	R10-KIR2DS4-m1	87	36	-0.72	0.49	0.59	-1.23	2.19E-01	-0.95	0.38	0.59	-1.62	1.04E-01
289	B*07:02-KIR2DS5-m2	27	13	0.06	1.06	0.28	0.20	8.38E-01	-0.15	0.86	0.28	-0.53	5.95E-01
290	B*08:01-KIR2DS5-m2	87	54	0.24	1.27	0.15	1.65	9.84E-02	0.24	1.27	0.15	1.65	9.92E-02
291	B*13:02-KIR2DS5-m2	6	3	-0.20	0.82	0.58	-0.35	7.26E-01	0.46	1.59	0.58	0.79	4.27E-01
292	B*14:01-KIR2DS5-m1	1	2	0.62	1.85	0.92	0.67	5.03E-01	1.28	3.58	0.93	1.38	1.68E-01
293	B*14:02-KIR2DS5-m1	8	3	-0.13	0.88	0.65	-0.19	8.47E-01	-0.15	0.86	0.65	-0.23	8.17E-01
294	B*15:01-KIR2DS5-m1	60	35	0.20	1.22	0.24	0.82	4.13E-01	-0.06	0.95	0.24	-0.23	8.18E-01
295	B*15:18-KIR2DS5-m2	3	1	-0.06	0.94	1.00	-0.06	9.51E-01	0.70	2.01	1.00	0.70	4.87E-01
296	B*18:01-KIR2DS5-m2	25	21	0.56	1.75	0.22	2.48	1.30E-02	0.33	1.39	0.23	1.47	1.43E-01
297	B*27:05-KIR2DS5-m1	11	6	0.42	1.52	0.49	0.86	3.91E-01	0.32	1.38	0.49	0.66	5.11E-01
298	B*35:01-KIR2DS5-m2	13	14	0.24	1.27	0.27	0.86	3.87E-01	0.08	1.08	0.27	0.29	7.74E-01
299	B*35:03-KIR2DS5-m1	7	2	-1.20	0.30	0.77	-1.55	1.20E-01	-1.12	0.33	0.77	-1.44	1.48E-01
300	B*35:08-KIR2DS5-m1	4	1	-1.45	0.24	1.12	-1.29	1.98E-01	-0.68	0.51	1.12	-0.60	5.48E-01
301	B*37:01-KIR2DS5-m2	8	1	-1.13	0.32	1.00	-1.13	2.60E-01	-0.79	0.45	1.00	-0.79	4.27E-01
302	B*38:01-KIR2DS5-m1	6	6	-0.25	0.78	0.50	-0.50	6.18E-01	-0.41	0.67	0.50	-0.82	4.13E-01
303	B*39:01-KIR2DS5-m2	2	2	0.58	1.79	0.71	0.82	4.14E-01	0.88	2.42	0.71	1.24	2.13E-01
304	B*39:06-KIR2DS5-m2	4	6	0.60	1.81	0.41	1.44	1.49E-01	0.74	2.09	0.41	1.79	7.31E-02
305	B*40:01-KIR2DS5-m2	37	18	0.08	1.08	0.24	0.33	7.40E-01	0.18	1.20	0.24	0.76	4.49E-01
306	B*40:02-KIR2DS5-m1	5	2	1.31	3.72	1.01	1.31	1.92E-01	0.93	2.53	1.01	0.92	3.57E-01
307	B*41:01-KIR2DS5-m2	1	1	1.02	2.79	1.00	1.02	3.06E-01	0.28	1.32	1.00	0.28	7.82E-01
308	B*44:02-KIR2DS5-m1	45	16	-0.54	0.58	0.31	-1.74	8.24E-02	-0.19	0.82	0.31	-0.62	5.36E-01
309	B*44:03-KIR2DS5-m1	14	6	-0.54	0.58	0.51	-1.06	2.87E-01	-0.22	0.80	0.51	-0.44	6.59E-01
310	B*45:01-KIR2DS5-m2	5	0										
311	B*49:01-KIR2DS5-m1	7	2	-0.88	0.41	0.82	-1.07	2.83E-01	-0.48	0.62	0.83	-0.58	5.63E-01
312	B*50:01-KIR2DS5-m1	8	5	0.45	1.56	0.62	0.73	4.68E-01	-0.17	0.85	0.62	-0.27	7.88E-01
313	B*51:01-KIR2DS5-m1	23	7	-0.06	0.94	0.46	-0.14	8.92E-01	0.19	1.21	0.46	0.42	6.73E-01

314	B*52:01-KIR2DS5-m2	4	1	-0.29	0.75	1.00	-0.29	7.75E-01	0.15	1.16	1.00	0.15	8.79E-01
315	B*53:01-KIR2DS5-m2	2	3	1.06	2.89	0.58	1.83	6.70E-02	0.66	1.93	0.58	1.13	2.58E-01
316	B*55:01-KIR2DS5-m1	9	1	-1.66	0.19	1.05	-1.59	1.12E-01	-1.77	0.17	1.05	-1.69	9.06E-02
317	B*56:01-KIR2DS5-m1	6	1	-0.99	0.37	1.16	-0.86	3.92E-01	-1.42	0.24	1.16	-1.23	2.20E-01
318	B*57:01-KIR2DS5-m1	5	2	-0.19	0.83	0.87	-0.21	8.30E-01	-0.35	0.70	0.87	-0.40	6.87E-01
319	B*58:01-KIR2DS5-m1	4	1	-0.10	0.91	1.16	-0.09	9.32E-01	-1.25	0.29	1.17	-1.07	2.85E-01
320	R10-KIR2DS5-m1	34	17	-0.04	0.96	0.34	-0.12	9.02E-01	0.09	1.09	0.34	0.25	8.02E-01
321	B*07:02-KIR3DL1-m2	98	42	-0.18	0.83	0.16	-1.13	2.56E-01	-0.25	0.78	0.16	-1.56	1.20E-01
322	B*08:01-KIR3DL1-m2	247	135	0.09	1.09	0.11	0.85	3.95E-01	0.04	1.04	0.11	0.34	7.36E-01
323	B*13:02-KIR3DL1-m2	20	8	-0.19	0.83	0.36	-0.52	6.00E-01	0.48	1.61	0.36	1.33	1.85E-01
324	B*14:01-KIR3DL1-m2	11	5	-0.09	0.92	0.45	-0.20	8.45E-01	-0.32	0.73	0.45	-0.70	4.82E-01
325	B*14:02-KIR3DL1-m2	34	15	0.06	1.06	0.26	0.21	8.31E-01	-0.04	0.96	0.26	-0.15	8.79E-01
326	B*15:01-KIR3DL1-m2	195	92	-0.10	0.90	0.12	-0.85	3.98E-01	-0.20	0.82	0.12	-1.72	8.51E-02
327	B*15:18-KIR3DL1-m2	8	4	0.45	1.57	0.50	0.89	3.73E-01	0.77	2.16	0.50	1.53	1.26E-01
328	B*18:01-KIR3DL1-m2	81	63	0.40	1.50	0.14	2.93	3.37E-03	0.19	1.21	0.14	1.40	1.61E-01
329	B*27:05-KIR3DL1-m2	46	21	-0.18	0.84	0.22	-0.80	4.26E-01	-0.13	0.88	0.22	-0.57	5.68E-01
330	B*35:01-KIR3DL1-m1	45	26	-0.52	0.60	0.60	-0.87	3.87E-01	0.09	1.09	0.60	0.14	8.87E-01
331	B*35:03-KIR3DL1-m1	14	13	1.34	3.81	1.06	1.26	2.09E-01	0.98	2.67	1.07	0.92	3.57E-01
332	B*35:08-KIR3DL1-m2	6	5	0.27	1.31	0.45	0.60	5.47E-01	0.51	1.67	0.45	1.13	2.59E-01
333	B*37:01-KIR3DL1-m2	19	3	-1.01	0.36	0.58	-1.74	8.13E-02	-1.25	0.29	0.58	-2.16	3.11E-02
334	B*38:01-KIR3DL1-m2	22	21	0.22	1.24	0.22	0.97	3.31E-01	0.00	1.00	0.23	0.00	9.98E-01
335	B*39:01-KIR3DL1-m2	19	9	-0.05	0.95	0.34	-0.15	8.78E-01	0.50	1.65	0.34	1.48	1.39E-01
336	B*39:06-KIR3DL1-m2	20	13	0.04	1.05	0.28	0.16	8.76E-01	0.16	1.17	0.28	0.56	5.78E-01
337	B*40:01-KIR3DL1-m1	106	61	1.24	3.46	0.76	1.64	1.02E-01	0.92	2.51	0.76	1.21	2.26E-01
338	B*40:02-KIR3DL1-m2	23	4	-0.76	0.47	0.50	-1.51	1.32E-01	-0.08	0.92	0.51	-0.16	8.76E-01
339	B*41:01-KIR3DL1-m2	7	2	-0.40	0.67	0.71	-0.56	5.76E-01	-0.06	0.94	0.71	-0.09	9.31E-01
340	B*44:02-KIR3DL1-m1	125	61	1.77	5.88	1.04	1.71	8.72E-02	1.23	3.42	1.04	1.18	2.36E-01
341	B*44:03-KIR3DL1-m2	37	18	0.01	1.01	0.24	0.06	9.51E-01	0.06	1.06	0.24	0.26	7.97E-01
342	B*45:01-KIR3DL1-m2	12	4	-0.06	0.94	0.50	-0.12	9.08E-01	-0.34	0.72	0.50	-0.67	5.06E-01
343	B*49:01-KIR3DL1-m2	19	7	0.30	1.36	0.38	0.79	4.27E-01	0.23	1.26	0.38	0.61	5.42E-01
344	B*50:01-KIR3DL1-m1	18	9	0.02	1.02	0.82	0.03	9.78E-01	-0.57	0.57	0.82	-0.69	4.89E-01
345	B*51:01-KIR3DL1-m2	72	25	-0.34	0.71	0.21	-1.64	1.02E-01	-0.30	0.74	0.21	-1.48	1.40E-01

346	B*52:01-KIR3DL1-m2	10	2	-0.50	0.61	0.71	-0.70	4.82E-01	0.11	1.12	0.71	0.16	8.73E-01
347	B*53:01-KIR3DL1-m2	8	4	0.31	1.36	0.50	0.61	5.39E-01	0.27	1.30	0.50	0.53	5.99E-01
348	B*55:01-KIR3DL1-m2	28	13	-0.02	0.98	0.28	-0.07	9.41E-01	0.03	1.03	0.28	0.11	9.16E-01
349	B*56:01-KIR3DL1-m2	10	3	-0.22	0.80	0.58	-0.39	7.00E-01	0.56	1.75	0.58	0.96	3.36E-01
350	B*57:01-KIR3DL1-m2	15	6	-0.11	0.89	0.41	-0.28	7.83E-01	0.05	1.05	0.41	0.12	9.03E-01
351	B*58:01-KIR3DL1-m2	6	4	0.17	1.19	0.50	0.34	7.33E-01	0.17	1.19	0.51	0.34	7.37E-01
352	R10-KIR3DL1-m1	88	36	-0.81	0.44	0.59	-1.38	1.66E-01	-0.98	0.38	0.59	-1.66	9.65E-02
353	B*07:02-KIR3DL2-m2	103	46	-0.14	0.87	0.16	-0.87	3.85E-01	-0.21	0.81	0.16	-1.35	1.77E-01
354	B*08:01-KIR3DL2-m2	262	141	0.09	1.09	0.10	0.85	3.98E-01	0.05	1.05	0.10	0.46	6.43E-01
355	B*13:02-KIR3DL2-m2	21	8	-0.23	0.79	0.36	-0.65	5.18E-01	0.46	1.58	0.36	1.27	2.03E-01
356	B*14:01-KIR3DL2-m2	11	5	-0.09	0.92	0.45	-0.20	8.45E-01	-0.32	0.73	0.45	-0.70	4.82E-01
357	B*14:02-KIR3DL2-m2	34	15	0.06	1.06	0.26	0.21	8.31E-01	-0.04	0.96	0.26	-0.15	8.79E-01
358	B*15:01-KIR3DL2-m2	203	101	-0.02	0.98	0.11	-0.21	8.38E-01	-0.15	0.86	0.11	-1.28	2.02E-01
359	B*15:18-KIR3DL2-m2	8	4	0.45	1.57	0.50	0.89	3.73E-01	0.77	2.16	0.50	1.53	1.26E-01
360	B*18:01-KIR3DL2-m2	83	64	0.40	1.49	0.14	2.93	3.35E-03	0.16	1.18	0.14	1.20	2.30E-01
361	B*27:05-KIR3DL2-m2	47	22	-0.15	0.86	0.22	-0.68	4.99E-01	-0.10	0.91	0.22	-0.45	6.50E-01
362	B*35:01-KIR3DL2-m2	48	30	-0.06	0.94	0.19	-0.30	7.65E-01	0.04	1.04	0.19	0.22	8.25E-01
363	B*35:03-KIR3DL2-m2	17	14	0.14	1.15	0.27	0.52	6.06E-01	0.07	1.07	0.27	0.25	8.06E-01
364	B*35:08-KIR3DL2-m2	6	5	0.27	1.31	0.45	0.60	5.47E-01	0.51	1.67	0.45	1.13	2.59E-01
365	B*37:01-KIR3DL2-m2	19	3	-1.01	0.36	0.58	-1.74	8.13E-02	-1.25	0.29	0.58	-2.16	3.11E-02
366	B*38:01-KIR3DL2-m2	22	21	0.22	1.24	0.22	0.97	3.31E-01	0.00	1.00	0.23	0.00	9.98E-01
367	B*39:01-KIR3DL2-m2	19	9	-0.05	0.95	0.34	-0.15	8.78E-01	0.50	1.65	0.34	1.48	1.39E-01
368	B*39:06-KIR3DL2-m2	20	14	0.12	1.12	0.27	0.43	6.66E-01	0.22	1.25	0.27	0.81	4.20E-01
369	B*40:01-KIR3DL2-m2	114	63	0.02	1.02	0.14	0.12	9.05E-01	0.09	1.10	0.14	0.67	5.02E-01
370	B*40:02-KIR3DL2-m2	24	4	-0.78	0.46	0.50	-1.56	1.20E-01	-0.08	0.92	0.51	-0.16	8.71E-01
371	B*41:01-KIR3DL2-m2	8	3	-0.10	0.90	0.58	-0.18	8.58E-01	0.04	1.04	0.58	0.07	9.46E-01
372	B*44:02-KIR3DL2-m2	134	62	-0.12	0.89	0.14	-0.88	3.78E-01	0.06	1.06	0.14	0.42	6.77E-01
373	B*44:03-KIR3DL2-m2	37	19	0.03	1.03	0.24	0.13	8.98E-01	0.10	1.10	0.24	0.41	6.80E-01
374	B*45:01-KIR3DL2-m2	13	4	-0.14	0.87	0.50	-0.27	7.88E-01	-0.45	0.64	0.50	-0.90	3.68E-01
375	B*49:01-KIR3DL2-m2	19	8	0.32	1.38	0.36	0.89	3.73E-01	0.21	1.23	0.36	0.58	5.59E-01
376	B*50:01-KIR3DL2-m2	20	11	0.24	1.27	0.31	0.78	4.34E-01	0.03	1.03	0.31	0.11	9.12E-01
377	B*51:01-KIR3DL2-m2	75	25	-0.35	0.70	0.21	-1.72	8.59E-02	-0.31	0.73	0.21	-1.50	1.34E-01

378	B*52:01-KIR3DL2-m2	10	2	-0.50	0.61	0.71	-0.70	4.82E-01	0.11	1.12	0.71	0.16	8.73E-01
379	B*53:01-KIR3DL2-m2	8	4	0.31	1.36	0.50	0.61	5.39E-01	0.27	1.30	0.50	0.53	5.99E-01
380	B*55:01-KIR3DL2-m2	28	13	-0.02	0.98	0.28	-0.07	9.41E-01	0.03	1.03	0.28	0.11	9.16E-01
381	B*56:01-KIR3DL2-m2	12	4	-0.04	0.96	0.50	-0.07	9.43E-01	0.76	2.14	0.50	1.51	1.32E-01
382	B*57:01-KIR3DL2-m2	15	6	-0.11	0.89	0.41	-0.28	7.83E-01	0.05	1.05	0.41	0.12	9.03E-01
383	B*58:01-KIR3DL2-m2	7	4	0.15	1.16	0.50	0.29	7.69E-01	0.16	1.18	0.50	0.32	7.48E-01
384	R10-KIR3DL2-m2	92	40	-0.14	0.87	0.17	-0.84	4.02E-01	-0.14	0.87	0.17	-0.83	4.09E-01
385	B*07:02-KIR3DS1-m1	28	17	0.50	1.65	0.32	1.55	1.22E-01	0.22	1.25	0.33	0.68	5.00E-01
386	B*08:01-KIR3DS1-m2	104	57	0.08	1.08	0.14	0.57	5.72E-01	0.07	1.08	0.14	0.51	6.08E-01
387	B*13:02-KIR3DS1-m2	6	3	-0.20	0.82	0.58	-0.35	7.26E-01	0.46	1.59	0.58	0.79	4.27E-01
388	B*14:01-KIR3DS1-m1	3	2	0.20	1.22	0.92	0.22	8.29E-01	0.99	2.69	0.92	1.07	2.84E-01
389	B*14:02-KIR3DS1-m2	10	4	0.26	1.30	0.50	0.53	5.99E-01	0.10	1.10	0.50	0.19	8.49E-01
390	B*15:01-KIR3DS1-m2	69	43	0.23	1.26	0.16	1.42	1.56E-01	-0.05	0.96	0.16	-0.28	7.79E-01
391	B*15:18-KIR3DS1-m2	3	1	-0.06	0.94	1.00	-0.06	9.51E-01	0.70	2.01	1.00	0.70	4.87E-01
392	B*18:01-KIR3DS1-m2	27	23	0.48	1.62	0.22	2.24	2.54E-02	0.21	1.24	0.22	0.98	3.25E-01
393	B*27:05-KIR3DS1-m1	16	10	0.29	1.34	0.44	0.67	5.05E-01	0.11	1.11	0.44	0.24	8.07E-01
394	B*35:01-KIR3DS1-m2	17	15	0.10	1.11	0.26	0.39	6.98E-01	0.07	1.08	0.26	0.28	7.79E-01
395	B*35:03-KIR3DS1-m1	9	5	-0.64	0.53	0.57	-1.13	2.57E-01	-0.34	0.71	0.57	-0.59	5.52E-01
396	B*35:08-KIR3DS1-m1	4	1	-1.38	0.25	1.12	-1.23	2.18E-01	-0.62	0.54	1.12	-0.55	5.81E-01
397	B*37:01-KIR3DS1-m2	9	1	-1.22	0.30	1.00	-1.22	2.24E-01	-0.85	0.43	1.00	-0.85	3.97E-01
398	B*38:01-KIR3DS1-m1	8	6	-0.46	0.63	0.49	-0.93	3.52E-01	-0.49	0.61	0.50	-1.00	3.18E-01
399	B*39:01-KIR3DS1-m2	4	4	1.05	2.86	0.50	2.09	3.68E-02	1.37	3.92	0.50	2.71	6.75E-03
400	B*39:06-KIR3DS1-m2	7	6	0.11	1.12	0.41	0.27	7.86E-01	0.42	1.52	0.41	1.01	3.13E-01
401	B*40:01-KIR3DS1-m2	43	23	0.10	1.10	0.21	0.46	6.45E-01	0.22	1.24	0.22	1.00	3.17E-01
402	B*40:02-KIR3DS1-m1	5	2	1.47	4.37	1.01	1.47	1.43E-01	1.10	3.01	1.01	1.10	2.73E-01
403	B*41:01-KIR3DS1-m2	1	1	1.02	2.79	1.00	1.02	3.06E-01	0.28	1.32	1.00	0.28	7.82E-01
404	B*44:02-KIR3DS1-m1	55	21	-0.56	0.57	0.29	-1.91	5.56E-02	-0.11	0.89	0.29	-0.39	6.96E-01
405	B*44:03-KIR3DS1-m1	17	5	-0.92	0.40	0.53	-1.73	8.35E-02	-0.60	0.55	0.53	-1.12	2.62E-01
406	B*45:01-KIR3DS1-m2	4	0										
407	B*49:01-KIR3DS1-m1	7	3	-0.44	0.65	0.74	-0.59	5.53E-01	-0.56	0.57	0.74	-0.76	4.49E-01
408	B*50:01-KIR3DS1-m2	8	4	0.61	1.84	0.50	1.21	2.25E-01	0.97	2.63	0.50	1.92	5.48E-02
409	B*51:01-KIR3DS1-m1	23	10	0.43	1.54	0.42	1.02	3.07E-01	0.28	1.32	0.42	0.65	5.13E-01

410	B*52:01-KIR3DS1-m1	4	1	0.33	1.39	1.42	0.23	8.17E-01	-0.24	0.78	1.42	-0.17	8.64E-01
411	B*53:01-KIR3DS1-m2	2	2	1.02	2.76	0.71	1.43	1.52E-01	0.73	2.08	0.71	1.03	3.02E-01
412	B*55:01-KIR3DS1-m1	14	3	-1.11	0.33	0.67	-1.66	9.64E-02	-1.30	0.27	0.67	-1.95	5.14E-02
413	B*56:01-KIR3DS1-m1	7	1	-1.12	0.33	1.16	-0.97	3.34E-01	-1.40	0.25	1.16	-1.21	2.27E-01
414	B*57:01-KIR3DS1-m1	7	2	-0.24	0.79	0.87	-0.27	7.86E-01	-0.40	0.67	0.87	-0.46	6.47E-01
415	B*58:01-KIR3DS1-m2	3	0										
416	R10-KIR3DS1-m1	37	19	-0.17	0.84	0.33	-0.52	6.03E-01	0.07	1.07	0.34	0.20	8.39E-01
417	B*07:02-KIR2DP1-m2	102	46	-0.13	0.87	0.16	-0.86	3.91E-01	-0.21	0.81	0.16	-1.35	1.77E-01
418	B*08:01-KIR2DP1-m1	258	135	-0.39	0.68	0.65	-0.60	5.49E-01	-0.61	0.54	0.65	-0.94	3.47E-01
419	B*13:02-KIR2DP1-m2	21	8	-0.23	0.79	0.36	-0.65	5.18E-01	0.46	1.58	0.36	1.27	2.03E-01
420	B*14:01-KIR2DP1-m2	11	5	-0.09	0.92	0.45	-0.20	8.45E-01	-0.32	0.73	0.45	-0.70	4.82E-01
421	B*14:02-KIR2DP1-m2	30	15	0.10	1.10	0.26	0.38	7.05E-01	0.00	1.00	0.26	0.01	9.94E-01
422	B*15:01-KIR2DP1-m2	200	100	-0.02	0.98	0.12	-0.20	8.43E-01	-0.15	0.86	0.12	-1.29	1.96E-01
423	B*15:18-KIR2DP1-m2	7	4	0.52	1.69	0.50	1.04	2.99E-01	0.89	2.44	0.50	1.77	7.64E-02
424	B*18:01-KIR2DP1-m2	82	62	0.39	1.48	0.14	2.84	4.54E-03	0.15	1.16	0.14	1.09	2.76E-01
425	B*27:05-KIR2DP1-m2	47	21	-0.18	0.83	0.22	-0.81	4.16E-01	-0.13	0.88	0.22	-0.58	5.63E-01
426	B*35:01-KIR2DP1-m2	47	30	-0.05	0.96	0.19	-0.24	8.10E-01	0.06	1.06	0.19	0.32	7.52E-01
427	B*35:03-KIR2DP1-m2	17	14	0.14	1.15	0.27	0.52	6.06E-01	0.07	1.07	0.27	0.25	8.06E-01
428	B*35:08-KIR2DP1-m2	6	5	0.27	1.31	0.45	0.60	5.47E-01	0.51	1.67	0.45	1.13	2.59E-01
429	B*37:01-KIR2DP1-m2	18	3	-0.94	0.39	0.58	-1.62	1.04E-01	-1.17	0.31	0.58	-2.02	4.36E-02
430	B*38:01-KIR2DP1-m1	22	17	-0.96	0.38	0.69	-1.39	1.65E-01	-0.24	0.79	0.69	-0.34	7.31E-01
431	B*39:01-KIR2DP1-m2	19	9	-0.05	0.95	0.34	-0.15	8.78E-01	0.50	1.65	0.34	1.48	1.39E-01
432	B*39:06-KIR2DP1-m2	19	13	0.11	1.12	0.28	0.40	6.91E-01	0.16	1.18	0.28	0.58	5.61E-01
433	B*40:01-KIR2DP1-m2	114	63	0.02	1.02	0.14	0.12	9.05E-01	0.09	1.10	0.14	0.67	5.02E-01
434	B*40:02-KIR2DP1-m2	24	4	-0.78	0.46	0.50	-1.56	1.20E-01	-0.08	0.92	0.51	-0.16	8.71E-01
435	B*41:01-KIR2DP1-m2	6	3	0.17	1.18	0.58	0.29	7.71E-01	0.30	1.35	0.58	0.52	6.05E-01
436	B*44:02-KIR2DP1-m2	130	61	-0.12	0.89	0.14	-0.84	4.03E-01	0.05	1.06	0.14	0.39	6.94E-01
437	B*44:03-KIR2DP1-m1	34	18	0.51	1.66	1.08	0.47	6.40E-01	0.83	2.29	1.08	0.77	4.43E-01
438	B*45:01-KIR2DP1-m2	12	3	-0.34	0.71	0.58	-0.59	5.56E-01	-0.65	0.52	0.58	-1.12	2.64E-01
439	B*49:01-KIR2DP1-m2	18	8	0.34	1.40	0.36	0.95	3.42E-01	0.22	1.24	0.36	0.61	5.44E-01
440	B*50:01-KIR2DP1-m2	20	10	0.22	1.24	0.32	0.68	4.99E-01	0.09	1.10	0.32	0.28	7.78E-01
441	B*51:01-KIR2DP1-m2	71	25	-0.34	0.71	0.21	-1.63	1.03E-01	-0.30	0.74	0.21	-1.47	1.42E-01

442	B*52:01-KIR2DP1-m2	9	2	-0.39	0.68	0.71	-0.54	5.86E-01	0.15	1.16	0.71	0.21	8.31E-01
443	B*53:01-KIR2DP1-m2	8	4	0.31	1.36	0.50	0.61	5.39E-01	0.27	1.30	0.50	0.53	5.99E-01
444	B*55:01-KIR2DP1-m2	27	13	0.04	1.05	0.28	0.16	8.75E-01	0.09	1.09	0.28	0.30	7.61E-01
445	B*56:01-KIR2DP1-m2	12	4	-0.04	0.96	0.50	-0.07	9.43E-01	0.76	2.14	0.50	1.51	1.32E-01
446	B*57:01-KIR2DP1-m2	15	6	-0.11	0.89	0.41	-0.28	7.83E-01	0.05	1.05	0.41	0.12	9.03E-01
447	B*58:01-KIR2DP1-m2	7	4	0.15	1.16	0.50	0.29	7.69E-01	0.16	1.18	0.50	0.32	7.48E-01
448	R10-KIR2DP1-m2	91	40	-0.14	0.87	0.17	-0.83	4.07E-01	-0.14	0.87	0.17	-0.82	4.12E-01
449	B*07:02-KIR3DP1-m2	103	46	-0.14	0.87	0.16	-0.87	3.85E-01	-0.21	0.81	0.16	-1.35	1.77E-01
450	B*08:01-KIR3DP1-m2	262	141	0.09	1.09	0.10	0.85	3.98E-01	0.05	1.05	0.10	0.46	6.43E-01
451	B*13:02-KIR3DP1-m2	21	8	-0.23	0.79	0.36	-0.65	5.18E-01	0.46	1.58	0.36	1.27	2.03E-01
452	B*14:01-KIR3DP1-m2	11	5	-0.09	0.92	0.45	-0.20	8.45E-01	-0.32	0.73	0.45	-0.70	4.82E-01
453	B*14:02-KIR3DP1-m2	34	15	0.06	1.06	0.26	0.21	8.31E-01	-0.04	0.96	0.26	-0.15	8.79E-01
454	B*15:01-KIR3DP1-m2	203	101	-0.02	0.98	0.11	-0.21	8.38E-01	-0.15	0.86	0.11	-1.28	2.02E-01
455	B*15:18-KIR3DP1-m2	8	4	0.45	1.57	0.50	0.89	3.73E-01	0.77	2.16	0.50	1.53	1.26E-01
456	B*18:01-KIR3DP1-m2	83	64	0.40	1.49	0.14	2.93	3.35E-03	0.16	1.18	0.14	1.20	2.30E-01
457	B*27:05-KIR3DP1-m2	47	22	-0.15	0.86	0.22	-0.68	4.99E-01	-0.10	0.91	0.22	-0.45	6.50E-01
458	B*35:01-KIR3DP1-m2	48	30	-0.06	0.94	0.19	-0.30	7.65E-01	0.04	1.04	0.19	0.22	8.25E-01
459	B*35:03-KIR3DP1-m2	17	14	0.14	1.15	0.27	0.52	6.06E-01	0.07	1.07	0.27	0.25	8.06E-01
460	B*35:08-KIR3DP1-m2	6	5	0.27	1.31	0.45	0.60	5.47E-01	0.51	1.67	0.45	1.13	2.59E-01
461	B*37:01-KIR3DP1-m2	19	3	-1.01	0.36	0.58	-1.74	8.13E-02	-1.25	0.29	0.58	-2.16	3.11E-02
462	B*38:01-KIR3DP1-m2	22	21	0.22	1.24	0.22	0.97	3.31E-01	0.00	1.00	0.23	0.00	9.98E-01
463	B*39:01-KIR3DP1-m2	19	9	-0.05	0.95	0.34	-0.15	8.78E-01	0.50	1.65	0.34	1.48	1.39E-01
464	B*39:06-KIR3DP1-m2	20	14	0.12	1.12	0.27	0.43	6.66E-01	0.22	1.25	0.27	0.81	4.20E-01
465	B*40:01-KIR3DP1-m2	114	63	0.02	1.02	0.14	0.12	9.05E-01	0.09	1.10	0.14	0.67	5.02E-01
466	B*40:02-KIR3DP1-m2	24	4	-0.78	0.46	0.50	-1.56	1.20E-01	-0.08	0.92	0.51	-0.16	8.71E-01
467	B*41:01-KIR3DP1-m2	8	3	-0.10	0.90	0.58	-0.18	8.58E-01	0.04	1.04	0.58	0.07	9.46E-01
468	B*44:02-KIR3DP1-m2	134	62	-0.12	0.89	0.14	-0.88	3.78E-01	0.06	1.06	0.14	0.42	6.77E-01
469	B*44:03-KIR3DP1-m2	37	19	0.03	1.03	0.24	0.13	8.98E-01	0.10	1.10	0.24	0.41	6.80E-01
470	B*45:01-KIR3DP1-m2	13	4	-0.14	0.87	0.50	-0.27	7.88E-01	-0.45	0.64	0.50	-0.90	3.68E-01
471	B*49:01-KIR3DP1-m2	19	8	0.32	1.38	0.36	0.89	3.73E-01	0.21	1.23	0.36	0.58	5.59E-01
472	B*50:01-KIR3DP1-m2	20	11	0.24	1.27	0.31	0.78	4.34E-01	0.03	1.03	0.31	0.11	9.12E-01
473	B*51:01-KIR3DP1-m2	75	25	-0.35	0.70	0.21	-1.72	8.59E-02	-0.31	0.73	0.21	-1.50	1.34E-01

474	B*52:01-KIR3DP1-m2	10	2	-0.50	0.61	0.71	-0.70	4.82E-01	0.11	1.12	0.71	0.16	8.73E-01
475	B*53:01-KIR3DP1-m2	8	4	0.31	1.36	0.50	0.61	5.39E-01	0.27	1.30	0.50	0.53	5.99E-01
476	B*55:01-KIR3DP1-m2	28	13	-0.02	0.98	0.28	-0.07	9.41E-01	0.03	1.03	0.28	0.11	9.16E-01
477	B*56:01-KIR3DP1-m2	12	4	-0.04	0.96	0.50	-0.07	9.43E-01	0.76	2.14	0.50	1.51	1.32E-01
478	B*57:01-KIR3DP1-m2	15	6	-0.11	0.89	0.41	-0.28	7.83E-01	0.05	1.05	0.41	0.12	9.03E-01
479	B*58:01-KIR3DP1-m2	7	4	0.15	1.16	0.50	0.29	7.69E-01	0.16	1.18	0.50	0.32	7.48E-01
480	R10-KIR3DP1-m2	92	40	-0.14	0.87	0.17	-0.84	4.02E-01	-0.14	0.87	0.17	-0.83	4.09E-01

ESM Table 8. Interation of HLA-C with KIR ligand numbers with the disease progression without (left panel) and with (right panel) adjustment for confounding effects: allelic frequency (left panel) or interactive frequency (right panel), estimated coefficient, hazard ratio, standard error, Z-score and p-value.

seq	C-KIR-model	Eff. Size		Unadjusted					Adjusted				
		n0	n1	coef	HR	SE	z	p	coef	HR	SE	z	p
1	C*01:02-KIR2DL1-m2	50	21	-0.23	0.79	0.22	-1.05	2.95E-01	0.00	1.00	0.22	-0.01	9.90E-01
2	C*02:02-KIR2DL1-m2	62	18	-0.45	0.64	0.24	-1.85	6.43E-02	-0.35	0.70	0.24	-1.46	1.43E-01
3	C*03:03-KIR2DL1-m1	101	55	0.25	1.28	1.06	0.23	8.17E-01	-0.10	0.91	1.07	-0.09	9.28E-01
4	C*03:04-KIR2DL1-m2	237	122	0.00	1.00	0.11	0.00	9.96E-01	-0.05	0.95	0.11	-0.44	6.58E-01
5	C*04:01-KIR2DL1-m2	87	43	-0.14	0.87	0.16	-0.89	3.74E-01	-0.07	0.93	0.16	-0.42	6.73E-01
6	C*05:01-KIR2DL1-m1	154	86	-0.03	0.97	0.80	-0.03	9.73E-01	-0.37	0.69	0.80	-0.46	6.44E-01
7	C*06:02-KIR2DL1-m1	88	36	-0.27	0.76	0.81	-0.33	7.40E-01	0.43	1.54	0.81	0.53	5.94E-01
8	C*07:01-KIR2DL1-m2	300	157	0.14	1.15	0.10	1.35	1.78E-01	0.11	1.12	0.10	1.12	2.62E-01
9	C*07:02-KIR2DL1-m1	138	64	-0.12	0.88	1.06	-0.12	9.06E-01	-1.44	0.24	1.06	-1.35	1.76E-01
10	C*07:04-KIR2DL1-m2	24	11	-0.07	0.93	0.31	-0.24	8.12E-01	0.30	1.35	0.31	0.98	3.28E-01
11	C*08:02-KIR2DL1-m2	39	20	0.06	1.07	0.23	0.27	7.84E-01	-0.07	0.93	0.23	-0.31	7.56E-01
12	C*12:02-KIR2DL1-m2	9	2	-0.39	0.68	0.71	-0.54	5.86E-01	0.15	1.16	0.71	0.21	8.31E-01
13	C*12:03-KIR2DL1-m2	59	50	0.30	1.34	0.15	1.95	5.07E-02	0.20	1.22	0.15	1.31	1.91E-01
14	C*14:02-KIR2DL1-m2	15	9	0.18	1.20	0.34	0.55	5.85E-01	0.18	1.20	0.34	0.54	5.88E-01
15	C*15:02-KIR2DL1-m2	34	13	-0.46	0.63	0.28	-1.63	1.04E-01	-0.25	0.78	0.28	-0.88	3.79E-01
16	C*15:05-KIR2DL1-m2	9	4	-0.27	0.76	0.50	-0.53	5.94E-01	-0.69	0.50	0.50	-1.37	1.71E-01
17	C*16:01-KIR2DL1-m1	36	15	0.14	1.15	1.09	0.13	8.98E-01	0.55	1.74	1.09	0.51	6.10E-01
18	C*16:02-KIR2DL1-m2	4	6	0.64	1.89	0.41	1.55	1.21E-01	0.60	1.82	0.41	1.45	1.47E-01
19	R10-KIR2DL1-m2	36	20	0.16	1.17	0.23	0.69	4.92E-01	0.15	1.16	0.23	0.65	5.17E-01
20	C*01:02-KIR2DL2-m1	25	6	-0.74	0.48	0.49	-1.51	1.31E-01	-0.49	0.61	0.49	-1.00	3.16E-01
21	C*02:02-KIR2DL2-m2	28	9	-0.35	0.70	0.34	-1.05	2.93E-01	-0.25	0.78	0.34	-0.74	4.57E-01
22	C*03:03-KIR2DL2-m1	54	27	-0.40	0.67	0.29	-1.37	1.71E-01	-0.21	0.81	0.29	-0.72	4.71E-01
23	C*03:04-KIR2DL2-m1	111	61	0.25	1.28	0.22	1.15	2.50E-01	0.34	1.40	0.22	1.56	1.19E-01
24	C*04:01-KIR2DL2-m2	43	20	-0.16	0.85	0.23	-0.70	4.84E-01	-0.06	0.94	0.23	-0.25	7.99E-01

25	C*05:01-KIR2DL2-m2	71	46	0.15	1.16	0.16	0.93	3.54E-01	-0.04	0.96	0.16	-0.25	8.06E-01
26	C*06:02-KIR2DL2-m2	46	18	-0.14	0.87	0.24	-0.58	5.64E-01	-0.24	0.79	0.24	-0.98	3.28E-01
27	C*07:01-KIR2DL2-m2	154	88	0.16	1.17	0.12	1.31	1.89E-01	0.09	1.09	0.12	0.72	4.73E-01
28	C*07:02-KIR2DL2-m2	68	33	-0.10	0.90	0.18	-0.56	5.75E-01	-0.03	0.97	0.18	-0.16	8.71E-01
29	C*07:04-KIR2DL2-m1	14	4	-0.58	0.56	0.64	-0.91	3.60E-01	-0.55	0.58	0.64	-0.86	3.90E-01
30	C*08:02-KIR2DL2-m2	22	6	-0.34	0.71	0.41	-0.84	4.04E-01	-0.30	0.74	0.41	-0.73	4.66E-01
31	C*12:02-KIR2DL2-m2	7	2	-0.19	0.82	0.71	-0.27	7.85E-01	0.48	1.61	0.71	0.67	5.05E-01
32	C*12:03-KIR2DL2-m2	32	29	0.45	1.57	0.19	2.34	1.94E-02	0.18	1.20	0.19	0.95	3.44E-01
33	C*14:02-KIR2DL2-m2	11	6	0.05	1.05	0.41	0.11	9.11E-01	0.21	1.23	0.41	0.50	6.17E-01
34	C*15:02-KIR2DL2-m2	10	4	-0.36	0.70	0.50	-0.71	4.79E-01	-0.37	0.69	0.50	-0.74	4.58E-01
35	C*15:05-KIR2DL2-m2	7	3	-0.38	0.68	0.58	-0.65	5.14E-01	-0.84	0.43	0.58	-1.45	1.48E-01
36	C*16:01-KIR2DL2-m1	17	9	0.72	2.06	0.51	1.40	1.61E-01	0.59	1.81	0.51	1.15	2.48E-01
37	C*16:02-KIR2DL2-m1	4	3	-0.45	0.64	0.82	-0.54	5.89E-01	-0.79	0.45	0.82	-0.96	3.39E-01
38	R10-KIR2DL2-m1	30	10	-0.79	0.46	0.46	-1.72	8.62E-02	-1.29	0.27	0.46	-2.80	5.07E-03
39	C*01:02-KIR2DL3-m1	48	21	-0.18	0.84	1.04	-0.17	8.62E-01	-0.43	0.65	1.04	-0.41	6.82E-01
40	C*02:02-KIR2DL3-m2	59	17	-0.45	0.64	0.25	-1.82	6.93E-02	-0.38	0.69	0.25	-1.52	1.27E-01
41	C*03:03-KIR2DL3-m1	95	52	0.47	1.61	0.55	0.86	3.88E-01	0.34	1.41	0.55	0.62	5.34E-01
42	C*03:04-KIR2DL3-m2	227	111	-0.04	0.96	0.11	-0.40	6.91E-01	-0.08	0.92	0.11	-0.76	4.49E-01
43	C*04:01-KIR2DL3-m1	77	42	1.48	4.39	1.03	1.44	1.49E-01	1.57	4.83	1.03	1.53	1.25E-01
44	C*05:01-KIR2DL3-m1	144	82	0.44	1.56	0.46	0.96	3.39E-01	0.38	1.46	0.46	0.82	4.13E-01
45	C*06:02-KIR2DL3-m2	83	33	-0.06	0.94	0.18	-0.35	7.29E-01	-0.10	0.91	0.18	-0.54	5.90E-01
46	C*07:01-KIR2DL3-m2	286	148	0.10	1.11	0.10	0.99	3.20E-01	0.08	1.09	0.10	0.82	4.14E-01
47	C*07:02-KIR2DL3-m1	127	57	-0.27	0.76	0.42	-0.65	5.17E-01	-0.78	0.46	0.42	-1.85	6.41E-02
48	C*07:04-KIR2DL3-m2	22	11	0.02	1.02	0.31	0.06	9.53E-01	0.39	1.48	0.31	1.28	2.02E-01
49	C*08:02-KIR2DL3-m2	37	18	0.01	1.01	0.24	0.03	9.76E-01	-0.14	0.87	0.24	-0.59	5.54E-01
50	C*12:02-KIR2DL3-m2	9	1	-1.08	0.34	1.00	-1.08	2.82E-01	-0.51	0.60	1.00	-0.51	6.11E-01
51	C*12:03-KIR2DL3-m2	50	44	0.33	1.39	0.16	2.04	4.09E-02	0.21	1.24	0.16	1.32	1.88E-01
52	C*14:02-KIR2DL3-m1	13	8	0.08	1.09	1.08	0.08	9.39E-01	-0.67	0.51	1.08	-0.62	5.32E-01
53	C*15:02-KIR2DL3-m2	33	12	-0.46	0.63	0.29	-1.57	1.16E-01	-0.19	0.83	0.29	-0.64	5.25E-01
54	C*15:05-KIR2DL3-m2	9	3	-0.41	0.66	0.58	-0.71	4.75E-01	-0.84	0.43	0.58	-1.45	1.46E-01
55	C*16:01-KIR2DL3-m1	35	15	0.19	1.21	1.05	0.18	8.58E-01	0.66	1.94	1.05	0.63	5.26E-01
56	C*16:02-KIR2DL3-m2	4	5	0.48	1.61	0.45	1.06	2.91E-01	0.43	1.53	0.45	0.95	3.44E-01

57	R10-KIR2DL3-m2	29	18	0.29	1.34	0.24	1.21	2.27E-01	0.14	1.15	0.24	0.59	5.58E-01
58	C*01:02-KIR2DL4-m2	50	22	-0.20	0.82	0.22	-0.91	3.62E-01	0.03	1.03	0.22	0.11	9.09E-01
59	C*02:02-KIR2DL4-m2	62	18	-0.45	0.64	0.24	-1.85	6.43E-02	-0.35	0.70	0.24	-1.46	1.43E-01
60	C*03:03-KIR2DL4-m2	103	56	0.06	1.06	0.14	0.39	6.94E-01	-0.01	0.99	0.14	-0.07	9.45E-01
61	C*03:04-KIR2DL4-m2	241	122	-0.02	0.98	0.11	-0.17	8.63E-01	-0.06	0.95	0.11	-0.52	6.04E-01
62	C*04:01-KIR2DL4-m2	89	43	-0.16	0.86	0.16	-0.96	3.36E-01	-0.08	0.92	0.16	-0.53	5.99E-01
63	C*05:01-KIR2DL4-m2	158	88	0.07	1.07	0.12	0.58	5.63E-01	-0.02	0.98	0.12	-0.17	8.62E-01
64	C*06:02-KIR2DL4-m2	91	38	-0.05	0.95	0.17	-0.28	7.80E-01	-0.09	0.91	0.17	-0.54	5.88E-01
65	C*07:01-KIR2DL4-m2	309	163	0.13	1.14	0.10	1.31	1.90E-01	0.12	1.13	0.10	1.20	2.30E-01
66	C*07:02-KIR2DL4-m2	141	65	-0.11	0.90	0.14	-0.79	4.31E-01	-0.04	0.96	0.14	-0.32	7.52E-01
67	C*07:04-KIR2DL4-m2	25	11	-0.09	0.92	0.31	-0.29	7.72E-01	0.27	1.32	0.31	0.89	3.72E-01
68	C*08:02-KIR2DL4-m2	43	20	0.03	1.03	0.23	0.14	8.92E-01	-0.10	0.90	0.23	-0.44	6.59E-01
69	C*12:02-KIR2DL4-m2	9	2	-0.39	0.68	0.71	-0.54	5.86E-01	0.15	1.16	0.71	0.21	8.31E-01
70	C*12:03-KIR2DL4-m2	59	54	0.34	1.40	0.15	2.30	2.15E-02	0.20	1.22	0.15	1.35	1.76E-01
71	C*14:02-KIR2DL4-m2	17	9	0.14	1.15	0.34	0.41	6.83E-01	0.17	1.18	0.34	0.49	6.23E-01
72	C*15:02-KIR2DL4-m2	34	13	-0.46	0.63	0.28	-1.63	1.04E-01	-0.25	0.78	0.28	-0.88	3.79E-01
73	C*15:05-KIR2DL4-m2	9	4	-0.27	0.76	0.50	-0.53	5.94E-01	-0.69	0.50	0.50	-1.37	1.71E-01
74	C*16:01-KIR2DL4-m2	38	16	-0.05	0.95	0.26	-0.19	8.52E-01	0.00	1.00	0.26	0.02	9.87E-01
75	C*16:02-KIR2DL4-m2	5	6	0.61	1.83	0.41	1.47	1.41E-01	0.59	1.81	0.41	1.44	1.50E-01
76	R10-KIR2DL4-m2	38	20	0.13	1.14	0.23	0.57	5.72E-01	0.14	1.15	0.23	0.62	5.37E-01
77	C*01:02-KIR2DL5-m1	26	8	-0.40	0.67	0.45	-0.88	3.76E-01	-0.70	0.50	0.46	-1.54	1.24E-01
78	C*02:02-KIR2DL5-m2	29	10	-0.34	0.71	0.32	-1.07	2.84E-01	-0.18	0.83	0.32	-0.57	5.68E-01
79	C*03:03-KIR2DL5-m2	47	33	0.21	1.23	0.18	1.13	2.58E-01	0.01	1.01	0.18	0.07	9.41E-01
80	C*03:04-KIR2DL5-m1	116	56	-0.12	0.89	0.22	-0.54	5.88E-01	-0.15	0.86	0.22	-0.67	5.02E-01
81	C*04:01-KIR2DL5-m2	47	25	-0.09	0.92	0.21	-0.43	6.71E-01	-0.06	0.94	0.21	-0.28	7.78E-01
82	C*05:01-KIR2DL5-m1	74	40	-0.29	0.75	0.24	-1.18	2.38E-01	-0.17	0.84	0.24	-0.72	4.73E-01
83	C*06:02-KIR2DL5-m1	44	18	-0.11	0.90	0.34	-0.32	7.51E-01	0.05	1.05	0.34	0.15	8.85E-01
84	C*07:01-KIR2DL5-m2	147	88	0.26	1.29	0.12	2.13	3.36E-02	0.23	1.26	0.12	1.94	5.29E-02
85	C*07:02-KIR2DL5-m1	68	31	-0.14	0.87	0.27	-0.53	5.94E-01	-0.06	0.94	0.27	-0.22	8.24E-01
86	C*07:04-KIR2DL5-m1	15	5	-0.78	0.46	0.61	-1.27	2.06E-01	-0.38	0.69	0.61	-0.61	5.39E-01
87	C*08:02-KIR2DL5-m2	19	7	-0.06	0.94	0.38	-0.15	8.80E-01	-0.14	0.87	0.38	-0.36	7.20E-01
88	C*12:02-KIR2DL5-m2	6	2	-0.23	0.80	0.71	-0.32	7.50E-01	0.18	1.20	0.71	0.25	8.01E-01

89	C*12:03-KIR2DL5-m2	31	26	0.29	1.34	0.20	1.43	1.54E-01	0.14	1.15	0.20	0.70	4.86E-01
90	C*14:02-KIR2DL5-m2	9	7	0.32	1.37	0.38	0.83	4.08E-01	0.37	1.45	0.38	0.98	3.29E-01
91	C*15:02-KIR2DL5-m1	12	10	1.51	4.53	0.67	2.27	2.33E-02	1.17	3.23	0.67	1.76	7.80E-02
92	C*15:05-KIR2DL5-m2	6	3	-0.19	0.83	0.58	-0.33	7.41E-01	-0.64	0.53	0.58	-1.10	2.70E-01
93	C*16:01-KIR2DL5-m1	17	6	-0.46	0.63	0.53	-0.87	3.83E-01	-0.10	0.91	0.53	-0.19	8.52E-01
94	C*16:02-KIR2DL5-m1	3	3	-0.14	0.87	0.82	-0.17	8.67E-01	-0.67	0.51	0.82	-0.81	4.17E-01
95	R10-KIR2DL5-m1	23	10	-0.62	0.54	0.46	-1.36	1.74E-01	-0.40	0.67	0.46	-0.88	3.78E-01
96	C*01:02-KIR2DS1-m1	21	6	-0.65	0.52	0.49	-1.33	1.84E-01	-0.75	0.47	0.49	-1.52	1.28E-01
97	C*02:02-KIR2DS1-m2	20	7	-0.31	0.73	0.38	-0.81	4.16E-01	-0.23	0.79	0.38	-0.61	5.44E-01
98	C*03:03-KIR2DS1-m1	35	24	0.22	1.25	0.29	0.76	4.46E-01	-0.10	0.90	0.29	-0.36	7.20E-01
99	C*03:04-KIR2DS1-m1	91	46	-0.07	0.93	0.22	-0.31	7.55E-01	-0.22	0.81	0.22	-0.97	3.32E-01
100	C*04:01-KIR2DS1-m1	39	21	0.00	1.00	0.32	-0.01	9.90E-01	-0.19	0.83	0.32	-0.59	5.57E-01
101	C*05:01-KIR2DS1-m2	54	34	0.12	1.13	0.18	0.68	4.99E-01	0.13	1.14	0.18	0.72	4.72E-01
102	C*06:02-KIR2DS1-m1	36	14	-0.13	0.88	0.35	-0.37	7.13E-01	0.10	1.10	0.35	0.28	7.81E-01
103	C*07:01-KIR2DS1-m2	119	71	0.19	1.21	0.13	1.48	1.39E-01	0.21	1.23	0.13	1.57	1.17E-01
104	C*07:02-KIR2DS1-m2	51	24	-0.03	0.97	0.21	-0.16	8.75E-01	0.03	1.03	0.21	0.14	8.87E-01
105	C*07:04-KIR2DS1-m1	11	4	-0.57	0.57	0.64	-0.89	3.72E-01	-0.25	0.78	0.64	-0.39	6.94E-01
106	C*08:02-KIR2DS1-m1	12	7	0.37	1.44	0.48	0.76	4.45E-01	0.62	1.85	0.48	1.28	2.02E-01
107	C*12:02-KIR2DS1-m2	4	1	-0.29	0.75	1.00	-0.29	7.75E-01	0.15	1.16	1.00	0.15	8.79E-01
108	C*12:03-KIR2DS1-m2	20	21	0.38	1.46	0.22	1.70	8.94E-02	0.17	1.19	0.22	0.76	4.49E-01
109	C*14:02-KIR2DS1-m1	7	4	-0.24	0.79	0.68	-0.35	7.24E-01	-0.36	0.70	0.68	-0.53	5.98E-01
110	C*15:02-KIR2DS1-m1	10	8	1.24	3.44	0.58	2.13	3.31E-02	0.95	2.59	0.58	1.64	1.00E-01
111	C*15:05-KIR2DS1-m1	3	3	1.10	3.01	1.16	0.95	3.41E-01	0.97	2.63	1.16	0.83	4.05E-01
112	C*16:01-KIR2DS1-m1	14	3	-1.19	0.30	0.65	-1.83	6.73E-02	-0.63	0.53	0.65	-0.97	3.33E-01
113	C*16:02-KIR2DS1-m2	2	2	0.59	1.80	0.71	0.83	4.08E-01	0.78	2.18	0.71	1.10	2.73E-01
114	R10-KIR2DS1-m1	14	8	-0.11	0.90	0.47	-0.23	8.17E-01	-0.17	0.84	0.47	-0.36	7.19E-01
115	C*01:02-KIR2DS2-m1	25	6	-0.76	0.47	0.49	-1.56	1.20E-01	-0.52	0.59	0.49	-1.07	2.86E-01
116	C*02:02-KIR2DS2-m2	30	9	-0.42	0.65	0.34	-1.26	2.09E-01	-0.30	0.74	0.34	-0.89	3.76E-01
117	C*03:03-KIR2DS2-m1	54	27	-0.42	0.66	0.29	-1.46	1.44E-01	-0.24	0.79	0.29	-0.84	4.01E-01
118	C*03:04-KIR2DS2-m1	111	62	0.25	1.29	0.22	1.17	2.43E-01	0.34	1.40	0.22	1.55	1.20E-01
119	C*04:01-KIR2DS2-m1	44	21	-0.06	0.94	0.32	-0.20	8.41E-01	0.06	1.07	0.32	0.20	8.44E-01
120	C*05:01-KIR2DS2-m2	72	47	0.15	1.16	0.16	0.95	3.40E-01	-0.03	0.97	0.16	-0.16	8.71E-01

121	C*06:02-KIR2DS2-m2	46	18	-0.14	0.87	0.24	-0.58	5.64E-01	-0.24	0.79	0.24	-0.98	3.28E-01
122	C*07:01-KIR2DS2-m2	156	91	0.19	1.21	0.12	1.57	1.15E-01	0.12	1.13	0.12	0.99	3.20E-01
123	C*07:02-KIR2DS2-m2	68	33	-0.10	0.90	0.18	-0.56	5.75E-01	-0.03	0.97	0.18	-0.16	8.71E-01
124	C*07:04-KIR2DS2-m1	14	4	-0.60	0.55	0.64	-0.95	3.42E-01	-0.58	0.56	0.64	-0.91	3.64E-01
125	C*08:02-KIR2DS2-m2	22	6	-0.34	0.71	0.41	-0.84	4.04E-01	-0.30	0.74	0.41	-0.73	4.66E-01
126	C*12:02-KIR2DS2-m2	7	2	-0.19	0.82	0.71	-0.27	7.85E-01	0.48	1.61	0.71	0.67	5.05E-01
127	C*12:03-KIR2DS2-m2	34	30	0.45	1.57	0.19	2.39	1.67E-02	0.20	1.22	0.19	1.05	2.93E-01
128	C*14:02-KIR2DS2-m2	11	6	0.05	1.05	0.41	0.11	9.11E-01	0.21	1.23	0.41	0.50	6.17E-01
129	C*15:02-KIR2DS2-m2	10	4	-0.36	0.70	0.50	-0.71	4.79E-01	-0.37	0.69	0.50	-0.74	4.58E-01
130	C*15:05-KIR2DS2-m2	7	3	-0.40	0.67	0.58	-0.68	4.95E-01	-0.83	0.44	0.58	-1.43	1.53E-01
131	C*16:01-KIR2DS2-m1	16	9	0.82	2.28	0.51	1.60	1.10E-01	0.71	2.03	0.51	1.38	1.69E-01
132	C*16:02-KIR2DS2-m1	4	3	-0.47	0.63	0.82	-0.57	5.70E-01	-0.82	0.44	0.82	-0.99	3.22E-01
133	R10-KIR2DS2-m1	30	10	-0.81	0.44	0.46	-1.77	7.72E-02	-1.32	0.27	0.46	-2.87	4.11E-03
134	C*01:02-KIR2DS3-m1	13	4	-0.39	0.68	0.56	-0.69	4.89E-01	-0.46	0.63	0.57	-0.82	4.11E-01
135	C*02:02-KIR2DS3-m2	19	5	-0.53	0.59	0.45	-1.19	2.35E-01	-0.34	0.71	0.45	-0.75	4.54E-01
136	C*03:03-KIR2DS3-m1	29	21	0.06	1.06	0.30	0.20	8.39E-01	-0.14	0.87	0.30	-0.48	6.33E-01
137	C*03:04-KIR2DS3-m1	57	36	0.37	1.45	0.24	1.54	1.23E-01	0.37	1.45	0.24	1.55	1.22E-01
138	C*04:01-KIR2DS3-m2	26	11	-0.32	0.72	0.31	-1.06	2.89E-01	-0.13	0.88	0.31	-0.42	6.75E-01
139	C*05:01-KIR2DS3-m1	34	19	-0.37	0.69	0.29	-1.29	1.96E-01	-0.35	0.70	0.29	-1.23	2.19E-01
140	C*06:02-KIR2DS3-m2	23	7	-0.30	0.74	0.38	-0.79	4.27E-01	-0.18	0.84	0.38	-0.47	6.42E-01
141	C*07:01-KIR2DS3-m2	69	46	0.28	1.33	0.16	1.81	7.05E-02	0.17	1.19	0.16	1.10	2.73E-01
142	C*07:02-KIR2DS3-m2	39	17	-0.24	0.79	0.25	-0.96	3.39E-01	0.01	1.01	0.25	0.05	9.64E-01
143	C*07:04-KIR2DS3-m1	8	3	-0.60	0.55	0.69	-0.88	3.78E-01	-0.23	0.80	0.69	-0.33	7.40E-01
144	C*08:02-KIR2DS3-m1	14	3	-0.83	0.44	0.64	-1.30	1.94E-01	-0.76	0.47	0.64	-1.20	2.30E-01
145	C*12:02-KIR2DS3-m2	3	2	0.13	1.13	0.71	0.18	8.59E-01	0.55	1.74	0.71	0.77	4.39E-01
146	C*12:03-KIR2DS3-m1	20	16	0.09	1.09	0.32	0.26	7.91E-01	-0.16	0.86	0.32	-0.49	6.28E-01
147	C*14:02-KIR2DS3-m2	5	6	0.64	1.90	0.41	1.56	1.19E-01	0.63	1.89	0.41	1.54	1.23E-01
148	C*15:02-KIR2DS3-m1	5	5	1.05	2.86	0.58	1.81	7.03E-02	0.82	2.27	0.58	1.41	1.59E-01
149	C*15:05-KIR2DS3-m2	3	1	-0.63	0.53	1.00	-0.63	5.30E-01	-0.97	0.38	1.00	-0.97	3.31E-01
150	C*16:01-KIR2DS3-m2	7	4	0.19	1.21	0.50	0.38	7.04E-01	0.39	1.47	0.50	0.77	4.44E-01
151	C*16:02-KIR2DS3-m1	2	1	-0.31	0.74	1.10	-0.28	7.80E-01	-1.30	0.27	1.10	-1.18	2.38E-01
152	R10-KIR2DS3-m1	16	4	-0.95	0.39	0.57	-1.67	9.57E-02	-0.42	0.66	0.57	-0.74	4.62E-01

153	C*01:02-KIR2DS4-m1	46	21	-0.30	0.74	1.05	-0.28	7.79E-01	-1.35	0.26	1.05	-1.28	2.01E-01
154	C*02:02-KIR2DS4-m2	61	18	-0.44	0.65	0.24	-1.81	6.98E-02	-0.35	0.71	0.24	-1.44	1.49E-01
155	C*03:03-KIR2DS4-m1	100	52	-0.99	0.37	0.58	-1.72	8.56E-02	-0.65	0.52	0.58	-1.12	2.62E-01
156	C*03:04-KIR2DS4-m2	230	115	-0.04	0.97	0.11	-0.32	7.47E-01	-0.07	0.93	0.11	-0.65	5.16E-01
157	C*04:01-KIR2DS4-m2	82	39	-0.19	0.82	0.17	-1.15	2.51E-01	-0.10	0.91	0.17	-0.58	5.65E-01
158	C*05:01-KIR2DS4-m1	149	86	1.24	3.44	0.76	1.64	1.02E-01	1.14	3.11	0.76	1.50	1.33E-01
159	C*06:02-KIR2DS4-m2	87	35	-0.08	0.93	0.18	-0.42	6.71E-01	-0.13	0.87	0.18	-0.75	4.53E-01
160	C*07:01-KIR2DS4-m2	292	155	0.13	1.13	0.10	1.22	2.22E-01	0.10	1.11	0.10	1.00	3.19E-01
161	C*07:02-KIR2DS4-m2	134	62	-0.12	0.89	0.14	-0.83	4.04E-01	-0.06	0.94	0.14	-0.45	6.56E-01
162	C*07:04-KIR2DS4-m2	24	11	-0.05	0.95	0.31	-0.16	8.72E-01	0.31	1.37	0.31	1.01	3.10E-01
163	C*08:02-KIR2DS4-m2	43	20	0.03	1.03	0.23	0.14	8.92E-01	-0.10	0.90	0.23	-0.44	6.59E-01
164	C*12:02-KIR2DS4-m2	9	2	-0.39	0.68	0.71	-0.54	5.86E-01	0.15	1.16	0.71	0.21	8.31E-01
165	C*12:03-KIR2DS4-m2	58	52	0.32	1.38	0.15	2.15	3.12E-02	0.20	1.22	0.15	1.35	1.79E-01
166	C*14:02-KIR2DS4-m2	17	9	0.14	1.15	0.34	0.41	6.83E-01	0.17	1.18	0.34	0.49	6.23E-01
167	C*15:02-KIR2DS4-m2	32	12	-0.49	0.61	0.29	-1.66	9.71E-02	-0.30	0.74	0.29	-1.02	3.09E-01
168	C*15:05-KIR2DS4-m2	9	3	-0.41	0.66	0.58	-0.71	4.75E-01	-0.84	0.43	0.58	-1.45	1.46E-01
169	C*16:01-KIR2DS4-m2	38	15	-0.07	0.93	0.26	-0.26	7.92E-01	-0.04	0.96	0.26	-0.15	8.81E-01
170	C*16:02-KIR2DS4-m2	4	6	0.63	1.88	0.41	1.54	1.24E-01	0.63	1.88	0.41	1.53	1.26E-01
171	R10-KIR2DS4-m2	35	18	0.09	1.10	0.24	0.39	6.94E-01	0.12	1.13	0.24	0.49	6.23E-01
172	C*01:02-KIR2DS5-m1	17	4	-0.52	0.60	0.56	-0.92	3.59E-01	-0.68	0.51	0.56	-1.21	2.26E-01
173	C*02:02-KIR2DS5-m1	15	6	0.31	1.37	0.51	0.61	5.40E-01	0.35	1.41	0.51	0.68	4.98E-01
174	C*03:03-KIR2DS5-m2	28	16	0.23	1.26	0.26	0.91	3.61E-01	0.09	1.10	0.26	0.37	7.13E-01
175	C*03:04-KIR2DS5-m1	75	36	-0.14	0.87	0.23	-0.60	5.47E-01	-0.23	0.79	0.23	-1.00	3.18E-01
176	C*04:01-KIR2DS5-m1	33	17	-0.03	0.97	0.33	-0.09	9.28E-01	-0.22	0.80	0.33	-0.68	4.99E-01
177	C*05:01-KIR2DS5-m2	46	23	0.04	1.04	0.21	0.17	8.68E-01	0.06	1.06	0.22	0.28	7.82E-01
178	C*06:02-KIR2DS5-m2	30	14	0.05	1.05	0.27	0.18	8.61E-01	0.06	1.07	0.27	0.23	8.15E-01
179	C*07:01-KIR2DS5-m2	104	62	0.27	1.31	0.14	1.93	5.41E-02	0.30	1.35	0.14	2.15	3.18E-02
180	C*07:02-KIR2DS5-m2	43	20	0.03	1.03	0.23	0.12	9.04E-01	0.07	1.07	0.23	0.30	7.65E-01
181	C*07:04-KIR2DS5-m1	10	3	-0.73	0.48	0.69	-1.06	2.89E-01	-0.60	0.55	0.69	-0.88	3.81E-01
182	C*08:02-KIR2DS5-m1	8	5	0.13	1.14	0.53	0.24	8.09E-01	0.36	1.44	0.53	0.69	4.93E-01
183	C*12:02-KIR2DS5-m2	4	1	-0.29	0.75	1.00	-0.29	7.75E-01	0.15	1.16	1.00	0.15	8.79E-01
184	C*12:03-KIR2DS5-m1	16	16	-0.15	0.86	0.32	-0.46	6.43E-01	-0.23	0.79	0.32	-0.73	4.65E-01

185	C*14:02-KIR2DS5-m2	5	2	-0.12	0.88	0.71	-0.17	8.62E-01	0.17	1.19	0.71	0.24	8.11E-01
186	C*15:02-KIR2DS5-m1	9	7	0.97	2.63	0.57	1.71	8.75E-02	0.62	1.85	0.57	1.08	2.78E-01
187	C*15:05-KIR2DS5-m1	3	3	1.10	3.00	1.16	0.95	3.44E-01	0.92	2.52	1.16	0.80	4.26E-01
188	C*16:01-KIR2DS5-m1	12	3	-0.85	0.43	0.65	-1.31	1.91E-01	-0.48	0.62	0.65	-0.74	4.62E-01
189	C*16:02-KIR2DS5-m2	2	2	0.59	1.80	0.71	0.83	4.08E-01	0.78	2.18	0.71	1.10	2.73E-01
190	R10-KIR2DS5-m2	13	8	0.16	1.18	0.36	0.46	6.47E-01	0.14	1.14	0.36	0.38	7.06E-01
191	C*01:02-KIR3DL1-m1	47	21	-0.57	0.57	1.05	-0.54	5.88E-01	-1.53	0.22	1.05	-1.45	1.47E-01
192	C*02:02-KIR3DL1-m2	61	18	-0.44	0.65	0.24	-1.81	6.98E-02	-0.35	0.71	0.24	-1.44	1.49E-01
193	C*03:03-KIR3DL1-m1	100	52	-0.98	0.37	0.58	-1.70	8.96E-02	-0.65	0.52	0.58	-1.12	2.64E-01
194	C*03:04-KIR3DL1-m2	230	115	-0.04	0.97	0.11	-0.32	7.47E-01	-0.07	0.93	0.11	-0.65	5.16E-01
195	C*04:01-KIR3DL1-m2	83	39	-0.20	0.82	0.17	-1.17	2.40E-01	-0.10	0.91	0.17	-0.58	5.61E-01
196	C*05:01-KIR3DL1-m1	149	86	1.25	3.50	0.76	1.66	9.76E-02	1.14	3.12	0.76	1.51	1.32E-01
197	C*06:02-KIR3DL1-m2	87	35	-0.08	0.93	0.18	-0.42	6.71E-01	-0.13	0.87	0.18	-0.75	4.53E-01
198	C*07:01-KIR3DL1-m2	292	155	0.13	1.13	0.10	1.22	2.22E-01	0.10	1.11	0.10	1.00	3.19E-01
199	C*07:02-KIR3DL1-m2	134	62	-0.12	0.89	0.14	-0.83	4.04E-01	-0.06	0.94	0.14	-0.45	6.56E-01
200	C*07:04-KIR3DL1-m2	24	11	-0.05	0.95	0.31	-0.16	8.72E-01	0.31	1.37	0.31	1.01	3.10E-01
201	C*08:02-KIR3DL1-m2	43	20	0.03	1.03	0.23	0.14	8.92E-01	-0.10	0.90	0.23	-0.44	6.59E-01
202	C*12:02-KIR3DL1-m2	9	2	-0.39	0.68	0.71	-0.54	5.86E-01	0.15	1.16	0.71	0.21	8.31E-01
203	C*12:03-KIR3DL1-m2	58	52	0.32	1.38	0.15	2.15	3.12E-02	0.20	1.22	0.15	1.35	1.79E-01
204	C*14:02-KIR3DL1-m2	17	9	0.14	1.15	0.34	0.41	6.83E-01	0.17	1.18	0.34	0.49	6.23E-01
205	C*15:02-KIR3DL1-m2	32	12	-0.49	0.61	0.29	-1.66	9.71E-02	-0.30	0.74	0.29	-1.02	3.09E-01
206	C*15:05-KIR3DL1-m2	9	3	-0.41	0.66	0.58	-0.71	4.75E-01	-0.84	0.43	0.58	-1.45	1.46E-01
207	C*16:01-KIR3DL1-m2	38	15	-0.07	0.93	0.26	-0.26	7.92E-01	-0.04	0.96	0.26	-0.15	8.81E-01
208	C*16:02-KIR3DL1-m2	4	6	0.63	1.88	0.41	1.54	1.24E-01	0.63	1.88	0.41	1.53	1.26E-01
209	R10-KIR3DL1-m2	35	18	0.09	1.10	0.24	0.39	6.94E-01	0.12	1.13	0.24	0.49	6.23E-01
210	C*01:02-KIR3DL2-m2	50	22	-0.20	0.82	0.22	-0.91	3.62E-01	0.03	1.03	0.22	0.11	9.09E-01
211	C*02:02-KIR3DL2-m2	62	18	-0.45	0.64	0.24	-1.85	6.43E-02	-0.35	0.70	0.24	-1.46	1.43E-01
212	C*03:03-KIR3DL2-m2	103	56	0.06	1.06	0.14	0.39	6.94E-01	-0.01	0.99	0.14	-0.07	9.45E-01
213	C*03:04-KIR3DL2-m2	241	122	-0.02	0.98	0.11	-0.17	8.63E-01	-0.06	0.95	0.11	-0.52	6.04E-01
214	C*04:01-KIR3DL2-m2	89	43	-0.16	0.86	0.16	-0.96	3.36E-01	-0.08	0.92	0.16	-0.53	5.99E-01
215	C*05:01-KIR3DL2-m2	158	88	0.07	1.07	0.12	0.58	5.63E-01	-0.02	0.98	0.12	-0.17	8.62E-01
216	C*06:02-KIR3DL2-m2	91	38	-0.05	0.95	0.17	-0.28	7.80E-01	-0.09	0.91	0.17	-0.54	5.88E-01

217	C*07:01-KIR3DL2-m2	309	163	0.13	1.14	0.10	1.31	1.90E-01	0.12	1.13	0.10	1.20	2.30E-01
218	C*07:02-KIR3DL2-m2	141	65	-0.11	0.90	0.14	-0.79	4.31E-01	-0.04	0.96	0.14	-0.32	7.52E-01
219	C*07:04-KIR3DL2-m2	25	11	-0.09	0.92	0.31	-0.29	7.72E-01	0.27	1.32	0.31	0.89	3.72E-01
220	C*08:02-KIR3DL2-m2	43	20	0.03	1.03	0.23	0.14	8.92E-01	-0.10	0.90	0.23	-0.44	6.59E-01
221	C*12:02-KIR3DL2-m2	9	2	-0.39	0.68	0.71	-0.54	5.86E-01	0.15	1.16	0.71	0.21	8.31E-01
222	C*12:03-KIR3DL2-m2	59	54	0.34	1.40	0.15	2.30	2.15E-02	0.20	1.22	0.15	1.35	1.76E-01
223	C*14:02-KIR3DL2-m2	17	9	0.14	1.15	0.34	0.41	6.83E-01	0.17	1.18	0.34	0.49	6.23E-01
224	C*15:02-KIR3DL2-m2	34	13	-0.46	0.63	0.28	-1.63	1.04E-01	-0.25	0.78	0.28	-0.88	3.79E-01
225	C*15:05-KIR3DL2-m2	9	4	-0.27	0.76	0.50	-0.53	5.94E-01	-0.69	0.50	0.50	-1.37	1.71E-01
226	C*16:01-KIR3DL2-m2	38	16	-0.05	0.95	0.26	-0.19	8.52E-01	0.00	1.00	0.26	0.02	9.87E-01
227	C*16:02-KIR3DL2-m2	5	6	0.61	1.83	0.41	1.47	1.41E-01	0.59	1.81	0.41	1.44	1.50E-01
228	R10-KIR3DL2-m2	38	20	0.13	1.14	0.23	0.57	5.72E-01	0.14	1.15	0.23	0.62	5.37E-01
229	C*01:02-KIR3DS1-m1	23	7	-0.49	0.61	0.47	-1.04	2.98E-01	-0.81	0.44	0.47	-1.72	8.56E-02
230	C*02:02-KIR3DS1-m2	20	8	-0.22	0.80	0.36	-0.62	5.36E-01	-0.25	0.78	0.36	-0.70	4.86E-01
231	C*03:03-KIR3DS1-m1	35	23	0.23	1.26	0.29	0.79	4.30E-01	-0.09	0.91	0.29	-0.32	7.48E-01
232	C*03:04-KIR3DS1-m1	88	45	0.01	1.01	0.22	0.03	9.78E-01	-0.18	0.84	0.22	-0.80	4.24E-01
233	C*04:01-KIR3DS1-m2	39	20	-0.11	0.90	0.23	-0.48	6.31E-01	-0.07	0.93	0.23	-0.29	7.69E-01
234	C*05:01-KIR3DS1-m2	56	30	0.00	1.00	0.19	-0.03	9.79E-01	0.04	1.05	0.19	0.23	8.15E-01
235	C*06:02-KIR3DS1-m1	32	11	-0.18	0.83	0.37	-0.49	6.21E-01	0.39	1.47	0.38	1.03	3.05E-01
236	C*07:01-KIR3DS1-m2	120	66	0.11	1.12	0.13	0.84	3.98E-01	0.15	1.16	0.13	1.12	2.65E-01
237	C*07:02-KIR3DS1-m2	51	24	-0.03	0.97	0.21	-0.16	8.75E-01	0.03	1.03	0.21	0.14	8.87E-01
238	C*07:04-KIR3DS1-m1	11	4	-0.51	0.60	0.64	-0.80	4.22E-01	-0.23	0.79	0.64	-0.37	7.13E-01
239	C*08:02-KIR3DS1-m1	12	6	0.21	1.24	0.50	0.43	6.70E-01	0.43	1.53	0.50	0.85	3.96E-01
240	C*12:02-KIR3DS1-m1	4	1	0.13	1.14	1.42	0.09	9.26E-01	-0.33	0.72	1.42	-0.23	8.17E-01
241	C*12:03-KIR3DS1-m2	18	19	0.42	1.52	0.24	1.77	7.64E-02	0.26	1.29	0.24	1.10	2.73E-01
242	C*14:02-KIR3DS1-m1	6	4	0.17	1.19	0.68	0.26	7.98E-01	-0.28	0.75	0.68	-0.42	6.77E-01
243	C*15:02-KIR3DS1-m1	10	8	1.29	3.65	0.58	2.23	2.57E-02	0.97	2.64	0.58	1.67	9.51E-02
244	C*15:05-KIR3DS1-m1	2	3	1.53	4.64	1.16	1.32	1.86E-01	1.26	3.52	1.16	1.09	2.78E-01
245	C*16:01-KIR3DS1-m1	14	2	-1.50	0.22	0.76	-1.97	4.91E-02	-1.09	0.34	0.76	-1.43	1.53E-01
246	C*16:02-KIR3DS1-m2	1	2	0.69	1.99	0.71	0.97	3.33E-01	0.80	2.22	0.71	1.12	2.62E-01
247	R10-KIR3DS1-m2	12	6	0.03	1.04	0.41	0.08	9.33E-01	0.44	1.55	0.41	1.06	2.91E-01
248	C*01:02-KIR2DP1-m2	50	21	-0.23	0.79	0.22	-1.05	2.95E-01	0.00	1.00	0.22	-0.01	9.90E-01

249	C*02:02-KIR2DP1-m2	62	18	-0.45	0.64	0.24	-1.85	6.43E-02	-0.35	0.70	0.24	-1.46	1.43E-01
250	C*03:03-KIR2DP1-m2	101	55	0.06	1.06	0.15	0.42	6.73E-01	-0.01	0.99	0.15	-0.08	9.34E-01
251	C*03:04-KIR2DP1-m2	239	122	-0.01	0.99	0.11	-0.10	9.24E-01	-0.05	0.95	0.11	-0.45	6.51E-01
252	C*04:01-KIR2DP1-m2	87	43	-0.14	0.87	0.16	-0.89	3.74E-01	-0.07	0.93	0.16	-0.42	6.73E-01
253	C*05:01-KIR2DP1-m1	154	86	0.09	1.09	0.80	0.11	9.14E-01	-0.36	0.70	0.80	-0.45	6.54E-01
254	C*06:02-KIR2DP1-m1	89	36	-0.27	0.76	0.81	-0.34	7.35E-01	0.42	1.52	0.81	0.52	6.06E-01
255	C*07:01-KIR2DP1-m2	301	157	0.13	1.14	0.10	1.27	2.02E-01	0.11	1.12	0.10	1.12	2.64E-01
256	C*07:02-KIR2DP1-m1	138	64	-0.02	0.98	1.06	-0.02	9.82E-01	-1.43	0.24	1.06	-1.34	1.79E-01
257	C*07:04-KIR2DP1-m2	24	11	-0.07	0.93	0.31	-0.24	8.12E-01	0.30	1.35	0.31	0.98	3.28E-01
258	C*08:02-KIR2DP1-m2	39	20	0.06	1.07	0.23	0.27	7.84E-01	-0.07	0.93	0.23	-0.31	7.56E-01
259	C*12:02-KIR2DP1-m2	9	2	-0.39	0.68	0.71	-0.54	5.86E-01	0.15	1.16	0.71	0.21	8.31E-01
260	C*12:03-KIR2DP1-m2	59	50	0.30	1.34	0.15	1.95	5.07E-02	0.20	1.22	0.15	1.31	1.91E-01
261	C*14:02-KIR2DP1-m2	15	9	0.18	1.20	0.34	0.55	5.85E-01	0.18	1.20	0.34	0.54	5.88E-01
262	C*15:02-KIR2DP1-m2	34	13	-0.46	0.63	0.28	-1.63	1.04E-01	-0.25	0.78	0.28	-0.88	3.79E-01
263	C*15:05-KIR2DP1-m2	9	4	-0.27	0.76	0.50	-0.53	5.94E-01	-0.69	0.50	0.50	-1.37	1.71E-01
264	C*16:01-KIR2DP1-m1	36	15	0.24	1.27	1.09	0.22	8.23E-01	0.57	1.76	1.09	0.52	6.03E-01
265	C*16:02-KIR2DP1-m2	4	6	0.64	1.89	0.41	1.55	1.21E-01	0.60	1.82	0.41	1.45	1.47E-01
266	R10-KIR2DP1-m2	36	20	0.16	1.17	0.23	0.69	4.92E-01	0.15	1.16	0.23	0.65	5.17E-01
267	C*01:02-KIR3DP1-m2	50	22	-0.20	0.82	0.22	-0.91	3.62E-01	0.03	1.03	0.22	0.11	9.09E-01
268	C*02:02-KIR3DP1-m2	62	18	-0.45	0.64	0.24	-1.85	6.43E-02	-0.35	0.70	0.24	-1.46	1.43E-01
269	C*03:03-KIR3DP1-m2	103	56	0.06	1.06	0.14	0.39	6.94E-01	-0.01	0.99	0.14	-0.07	9.45E-01
270	C*03:04-KIR3DP1-m2	241	122	-0.02	0.98	0.11	-0.17	8.63E-01	-0.06	0.95	0.11	-0.52	6.04E-01
271	C*04:01-KIR3DP1-m2	89	43	-0.16	0.86	0.16	-0.96	3.36E-01	-0.08	0.92	0.16	-0.53	5.99E-01
272	C*05:01-KIR3DP1-m2	158	88	0.07	1.07	0.12	0.58	5.63E-01	-0.02	0.98	0.12	-0.17	8.62E-01
273	C*06:02-KIR3DP1-m2	91	38	-0.05	0.95	0.17	-0.28	7.80E-01	-0.09	0.91	0.17	-0.54	5.88E-01
274	C*07:01-KIR3DP1-m2	309	163	0.13	1.14	0.10	1.31	1.90E-01	0.12	1.13	0.10	1.20	2.30E-01
275	C*07:02-KIR3DP1-m2	141	65	-0.11	0.90	0.14	-0.79	4.31E-01	-0.04	0.96	0.14	-0.32	7.52E-01
276	C*07:04-KIR3DP1-m2	25	11	-0.09	0.92	0.31	-0.29	7.72E-01	0.27	1.32	0.31	0.89	3.72E-01
277	C*08:02-KIR3DP1-m2	43	20	0.03	1.03	0.23	0.14	8.92E-01	-0.10	0.90	0.23	-0.44	6.59E-01
278	C*12:02-KIR3DP1-m2	9	2	-0.39	0.68	0.71	-0.54	5.86E-01	0.15	1.16	0.71	0.21	8.31E-01
279	C*12:03-KIR3DP1-m2	59	54	0.34	1.40	0.15	2.30	2.15E-02	0.20	1.22	0.15	1.35	1.76E-01
280	C*14:02-KIR3DP1-m2	17	9	0.14	1.15	0.34	0.41	6.83E-01	0.17	1.18	0.34	0.49	6.23E-01

281	C*15:02-KIR3DP1-m2	34	13	-0.46	0.63	0.28	-1.63	1.04E-01	-0.25	0.78	0.28	-0.88	3.79E-01
282	C*15:05-KIR3DP1-m2	9	4	-0.27	0.76	0.50	-0.53	5.94E-01	-0.69	0.50	0.50	-1.37	1.71E-01
283	C*16:01-KIR3DP1-m2	38	16	-0.05	0.95	0.26	-0.19	8.52E-01	0.00	1.00	0.26	0.02	9.87E-01
284	C*16:02-KIR3DP1-m2	5	6	0.61	1.83	0.41	1.47	1.41E-01	0.59	1.81	0.41	1.44	1.50E-01
285	R10-KIR3DP1-m2	38	20	0.13	1.14	0.23	0.57	5.72E-01	0.14	1.15	0.23	0.62	5.37E-01

ESM Table 9. HLA-DQ residues associated with progression to T1D (listed in ESM Table 5) and residues in perfect LD to them within the HLA-DQ allelic molecules of the study

Key residue identified by HOH analysis	Residue(s) in perfect LD
–16α	α66
α22	α49, α51
α23	α53, α73, α184
α31*	α37, α47, α48, α50, α212
α44	–––
α72*	α104, α153, α158, α160, α172
α76	–––
α157	–––
–18β	β188
–10β	β28, β30, β37, β38, β46, β47, β52, β77, β140, β185
β9	β56, β70
β13	β45, β167
β23	–––
β26	–––
β55	β74
β57	–––
β66	β67, β75
β71	–––
β135	–––

ESM Table 10. KIR-HLAI cross tabulation and related definition of interactions

**HLAI**	**KIR receptors**				
**G**	0	1	2	3	4
O/O	c00	c01	c02	c03	c04
A/O	c10	c11	c12	c13	c14
A/A	c20	c21	c22	c23	c24

ref c00+c01+c02+c03+c04+c10+c20

int c11+c12+c13+c14+c21+c22+c23+c24

ESM Figure 1. Up to four different HLAI molecules from heterozygous genotype with and without a KIR receptor.

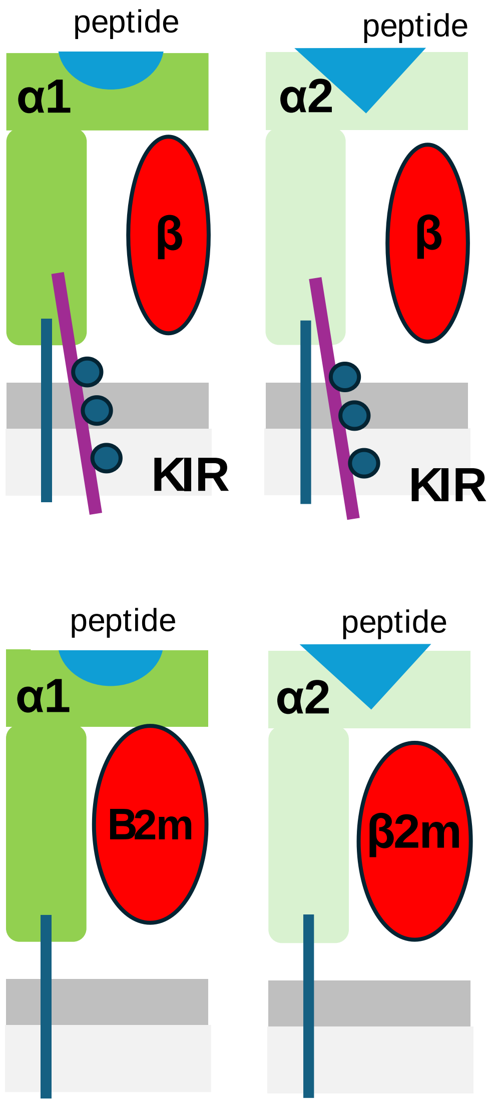

ESM Figure 2.

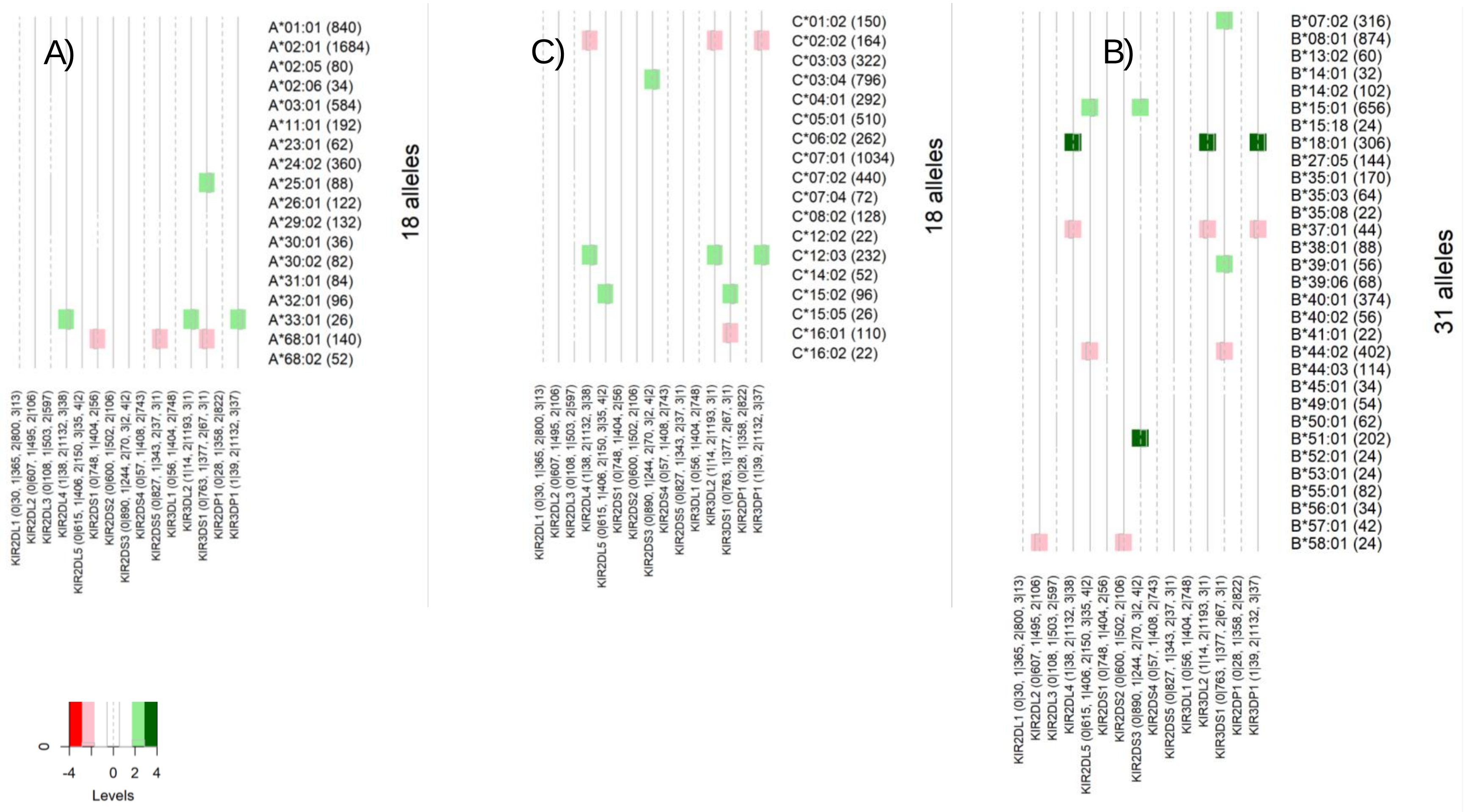

A)
A*01:01 (840)
A*02:01 (1684)
A*02:05 (80)
A*02:06 (34)
A*03:01 (584)
A*11:01 (192)
A*23:01 (62)
A*24:02 (360)
A*25:01 (88)
A*26:01 (122)
A*29:02 (132)
A*30:01 (36)
A*30:02 (82)
A*31:01 (84)
A*32:01 (96)
A*33:01 (26)
A*68:01 (140)
A*68:02 (52)
18 alleles
C)
C*01:02 (150)
C*02:02 (164)
C*03:03 (322)
C*03:04 (796)
C*04:01 (292)
C*05:01 (510)
C*06:02 (262)
C*07:01 (1034)
C*07:02 (440)
C*07:04 (72)
C*08:02 (128)
C*12:02 (22)
C*12:03 (232)
C*14:02 (52)
C*15:02 (96)
C*15:05 (26)
C*16:01 (110)
C*16:02 (22)
18 alleles
B)
B*07:02 (316)
B*08:01 (874)
B*13:02 (60)
B*14:01 (32)
B*14:02 (102)
B*15:01 (656)
B*15:18 (24)
B*18:01 (306)
B*27:05 (144)
B*35:01 (170)
B*35:03 (64)
B*35:08 (22)
B*37:01 (44)
B*38:01 (88)
B*39:01 (56)
B*39:06 (68)
B*40:01 (374)
B*40:02 (56)
B*41:01 (22)
B*44:02 (402)
B*44:03 (114)
B*45:01 (34)
B*49:01 (54)
B*50:01 (62)
B*51:01 (202)
B*52:01 (24)
B*53:01 (24)
B*55:01 (82)
B*56:01 (34)
B*57:01 (42)
B*58:01 (24)
31 alleles
KIR2DL1 (0|30, 1|365, 2|800, 3|13)
KIR2DL2 (0|607, 1|495, 2|106)
KIR2DL3 (0|108, 1|503, 2|597)
KIR2DL4 (1|38, 2|1132, 3|38)
KIR2DL5 (0|615, 1|406, 2|150, 3|35, 4|2)
KIR2DS1 (0|748, 1|404, 2|56)
KIR2DS2 (0|600, 1|502, 2|106)
KIR2DS3 (0|890, 1|244, 2|70, 3|2, 4|2)
KIR2DS4 (0|57, 1|408, 2|743)
KIR2DS5 (0|827, 1|343, 2|37, 3|1)
KIR3DL1 (0|56, 1|404, 2|748)
KIR3DL2 (1|14, 2|1193, 3|1)
KIR3DS1 (0|763, 1|377, 2|67, 3|1)
KIR2DP1 (0|28, 1|358, 2|822)
KIR3DP1 (1|39, 2|1132, 3|37)
0
-4
0
2
4
Levels
